# C3 and C6 Modification‐Specific OYE Biotransformations of Synthetic Carvones and Sequential BVMO Chemoenzymatic Synthesis of Chiral Caprolactones†

**DOI:** 10.1002/chem.201805219

**Published:** 2019-01-15

**Authors:** Issa S. Issa, Helen S. Toogood, Linus O. Johannissen, James Raftery, Nigel S. Scrutton, John M. Gardiner

**Affiliations:** ^1^ Manchester Institute of Biotechnology and the School of Chemistry The University of Manchester 131 Princess Street Manchester M1 7DN UK; ^2^ BBSRC/EPSRC Manchester Synthetic Biology Research Centre, for Fine and Specialty Chemicals (SYNBIOCHEM), Manchester Institute of Biotechnology and the School of Chemistry The University of Manchester 131 Princess Street Manchester M1 7DN UK

**Keywords:** biocatalysis, Baeyer–Villiger monooxygenases, carvone derivatives, enzyme catalysis, lactones, OYEs

## Abstract

The scope for biocatalytic modification of non‐native carvone derivatives for speciality intermediates has hitherto been limited. Additionally, caprolactones are important feedstocks with diverse applications in the polymer industry and new non‐native terpenone‐derived biocatalytic caprolactone syntheses are thus of potential value for industrial biocatalytic materials applications. Biocatalytic reduction of synthetic analogues of *R*‐(−)‐carvone with additional substituents at C3 or C6, or both C3 and C6, using three types of OYEs (OYE2, PETNR and OYE3) shows significant impact of both regio‐substitution and the substrate diastereomer. Bioreduction of (−)‐carvone derivatives substituted with a Me and/or OH group at C6 is highly dependent on the diastereomer of the substrate. Derivatives bearing C6 substituents larger than methyl moieties are not substrates. Computer docking studies of PETNR with both (6*S*)‐Me and (6*R*)‐Me substituted (−)‐carvone provides a model consistent with the outcomes of bioconversion. The products of bioreduction were efficiently biotransformed by the Baeyer–Villiger monooxygenase (BVase) CHMO_Phi1 to afford novel trisubstituted lactones with complete regioselectivity to provide a new biocatalytic entry to these chiral caprolactones. This provides both new non‐native polymerization feedstock chemicals, but also with enhanced efficiency and selectivity over native (+)‐dihydrocarvone Baeyer–Villigerase expansion. Optimum enzymatic reactions were scaled up to 60–100 mg, demonstrating the utility for preparative biocatalytic synthesis of both new synthetic scaffold‐modified dihydrocarvones and efficient biocatalytic entry to new chiral caprolactones, which are potential single‐isomer chiral polymer feedstocks.

Diastereoisomers of (*R*)‐(−)‐carvone and (+)‐dihydrocarvone are sources of crucial building blocks as chiral precursors in the synthesis of many natural and non‐natural organic compounds.^1^ Dihydrocarvone‐derived caprolactones (by Baeyer–Villiger ring expansion) have also seen applications to ring‐opening polymerizations (ROP).

Conversion of (*R*)‐(−)‐carvone to (+)‐dihydrocarvone isomers has been widely reported using chemical catalysis^2^ and biocatalysis using isolated enzymes^3^ or whole cells.^4^ Several members of the Old Yellow Enzyme (OYE) family have been shown to catalyse ene‐reduction of (−)‐ or (+)‐carvone in good yields and with high diastereoselectivity in favour of the (2*R*)‐isomer.5a This includes pentaerythritol tetranitrate reductase (PETNR) from *Enterobacter cloacae*,5b OYE1 from *Saccharomyces pastorianus* 5c and thermostable Old Yellow Enzyme (TOYE) from *Thermoanaerobacter pseudethanolicus*.5d Models accounting for the stereochemical outcomes, and mutants reversing selectivity have been reported,5a,5b,5e and approaches to process improvements are described.5f

Carvone derivatives with additional alkyl or heteroatom substituents (e.g., hydroxyl) have also been useful synthetic chirons,1c,^6^ and new variants offer value as new precursors. However, there are no reports of bioreduction of (−)‐carvone substituted at C6 or with additional scaffold changes including substitution at the β‐alkene carbon (C3). Furthermore, native terpenone Baeyer–Villigerase‐derived caprolactones, including (+)‐dihydrocarvone, have recently been reported providing biocatalytic access to substrates for polymerizations,^7^ but synthetically‐modified terpenones have not been previously evaluated.

This paper reports the evaluation of biocatalytic enone reductions of a matrix of synthetic carvone derivatives, encompassing diasteromerically pure 6‐methyl‐(−)‐carvones and 6‐hydroxy‐carvones. It describes the impact of the configuration (6*R* or 6*S*), substituent types, and also assesses the effect on bioreductions of locating an additional methyl at C3 (regioisomeric with C6 methyl‐substituted (−)‐carvones) (Figure 1).

**Figure 1:**
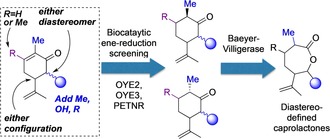
Scope for synthetically modified carvone skeleton: evaluating substituent and diastereoisomer effects on ene‐reductase biocatalysis, and potential for chem‐biocat‐biocat route to caprolactones.

The ene‐reductases OYE2 and OYE3 from *Saccharomyces cerevisiae* 8 and PETNR^9^ were screened for activity against substituted carvone derivatives. These results define how the diastereo‐structure of these substrates determines optimum enzymes for several new highly selective biotransformations. Furthermore, the products from bioreduction of C6‐methyl‐ and C3‐methyl carvones are shown to be bio‐oxidized by a second enzymatic step using the Baeyer–Villigerase CHMO_Phi1 (from *Rhodococcus* sp. Phi1^10^). This affords the first examples of substituted dihydrocarvone biocatalytic ring expansion to synthetically valuable caprolactones (Table 1) indicating that these offer enhanced efficiency and stereochemical selectivity over native carvone. Additionally, preparative scale synthesis of enantiopure lactones by means of this chemical‐biocat‐biocat sequence is demonstrated (Figure 1). This expands the potential scope of such materials precursors and a viable entry to single‐isomer ROP components directly.

Synthesis of both 6‐methyl carvone diastereomers (6*S*)‐**2** and (6*R*)‐**3** was effected through methylation of the lithium enolate prepared from (−)‐carvone (**1**), with a final epimer equilibration, both diastereomers being isolated through chromatography (Scheme 1; SI).6c, 11 An X‐ray crystal structure of (5*R*, 6*S*)‐6‐methylcarvone diastereomer **2**, further confirmed structural assignments (Figure S16).^12^ The pure 6‐hydroxy carvone diastereomers were also prepared from (−)‐ and (+)‐carvone enolates through Rubottom oxidation6b, 13 and also isolated through chromatography (Supporting Information), affording compounds **4** and **5** from (−)‐carvone, and **7** and **8** from (+)‐carvone, respectively. This provided a set of six C6‐substituted (−)‐ and (+)‐carvone derivatives. The 3‐methyl substituted analogue **10** was prepared from (+)‐carvone through methyl Grignard addition followed by a 1,3‐oxidative transposition using PCC,^14^ affording 3‐methyl‐(−)‐carvone **10** in over 80 % isolated yield (Scheme 1).

**Scheme 1:**
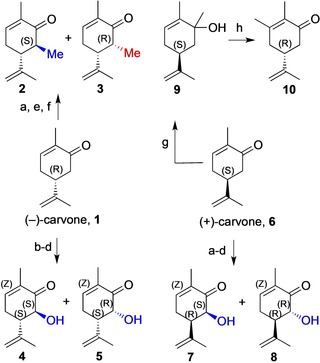
Synthesis of 3‐ and 6‐modified carvone substrates. a) LDA, THF, −78 °C; b) TMSCl; c) MCPBA, CH_2_Cl_2_; d) HCl (1.5 m); e) MeI; f) DBU; g) MeMgBr, h) PCC, CH_2_Cl_2_. Yields (**2**+**3**)=85 %, (**4**+**5**)=58 %, (**7**+**8**)=43 %, (**10**)=84 %.

We previously reported 24 h biotransformations of PETNR (2 μm) with (−)‐carvone **1**.^5^ Here, whilst finding that OYE2‐catalysed reduction of **1** was similarly effective over 24 h, a significantly shorter reaction time of 2 h provided (2*R*)‐(−)‐dihydrocarvone in 95 % yield and 96 % *de* (Scheme 2, Table 1 Entries 1, 2, Table S4). We established that the 24 h reaction time using PETNR^5^ can also be reduced to afford similar outcome after 2 h, indeed with enhanced yield (Table 1, Entry 1). Both these reaction times (2 h or 24 h) were also evaluated for OYE3‐catalysed reduction of **1**, with the same short reaction time affording yields of 80 % and *de* of 95 % (Table S5). With highly efficient, selective and short biocatalytic reaction times for **1** using these three ene‐reductases, optimal conditions were determined with seven synthetically modified carvone derivatives (Scheme 1).

**Scheme 2:**
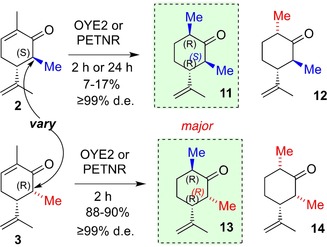
Bioreduction of 6‐Me‐(−)‐carvones.

**Table 1:** Biocatalytic reduction of 6‐substituted carvones by PETNR/ OYE2.

Entry	Substrate	Major	Time	OYE2		PETNR	
		Product	[h]	Yield [%]	*de* [%]	Yield [%]	*de* [%]
1	**1**	2*R*‐DHC^[a]^	2	**84**	**93**	**95**	**96**
2	**1**	2*R*‐DHC^[a]^	24	**82**	**85**	**78**	**95**
3	**2**	**11**	2	17	≥99	7	≥99
4	**2**	**11**	24	15	≥99	17	≥99
5	**3**	**13**	2	**90**	**≥99**	**88**	**≥99**
6	**3**	**13**	24	53	≥99	43	≥99
7	**4**	**15**	2	28	≥99	**66**	**≥99**
8	**4**	**15**	24	40	≥99	**70**	**≥99**
9	**5**	**17**	2	11	≥99	**80**	**≥99**
10	**5**	**17**	24	30	16	**70**	**≥99**
11	**8**	**19**	2	7	57	95	28
12	**8**	**19**	24	6	57	85	28

[a] 6*R*‐dihydroxycarvone. General reaction conditions: enzyme (2–10 μm), substrate (5 mm), 50 mm KP buffer solution (pH 7.0), NADP^+^ (15 μm), GDH (10 U), glucose or glucose‐6‐phosphate (15 mm), 30 °C at 130 rpm.

Biotransformation of the two C6‐Me diastereomers, **2** and **3**, with OYE2 and PETNR at 2 and 24 h, showed that the yield from the (5*R*, 6*S*) diastereomer **2** was low with both enzymes, although in both cases the product was formed with very high diastereocontrol (Scheme 2, Table 1, Entries 3 and 4) in favour of (2*R*, 5*R*, 6*S*)‐**11**. However, the (5*R*, 6*R*) diastereomer **3** was converted to (2*R*, 5*R*, 6*R*)‐**13** within 2 h in 88–90 % yields (PETNR; OYE2) and ≥99 % diastereoselectivity (Scheme 2; Table 1, Entry 5). Thus, both 6‐Me diastereomers undergo bioreduction with high diastereofacial control, introducing *R*‐configuration at the new chiral centre.^5^ We also evaluated chemical dithionite reduction of **2** and **3**, (also to provide reference samples) and observed that this provides preference for the same diastereomer as biocatalysis but with much lower diastereomer ratios of 4:1 to 8:1. Notably, OYE3 was a very poor enzyme for this biotransformation. Bioreduction of synthetic (−)‐carvones substituted with C6 groups larger than Me such as C_2_H_5_, CHOHCH_3_ and CH_2_Ph^15^ showed no observable product formation using up to 10 μm of biocatalysts OYE2 or PETNR.

These data therefore indicate that the configuration at C6 does not affect the binding and/or orientation of the substrate with respect to diastereofacial selectivity. Additionally, the substitutions show higher stereoselectivity than the parent compound **1**, but the stereo‐configuration of the methyl at C6 does significantly impact the rate of conversion [(6*S*)‐**2** is slow (6*R*)‐**3** is fast] and also the yield. This reduction in conversions was also demonstrated using a mixture of diastereomers **2** and **3** (Figure S3), where a more rapid depletion of **3** and formation of **13** was observed.

To examine whether the bound conformation of the substrate explains the major product enantiomers, DFT models^16^ were created from a crystal structure of PETNR with bound 2‐cyclohexanone (PDB ID 1GVQ), using first‐shell amino acids truncated at the C_β_, a truncated FMN and the 6*S*‐ and 6*R*‐Me carvones (Figure 2 and Figure S15) built using the 2‐cyclohexanone structure.

**Figure 2:**
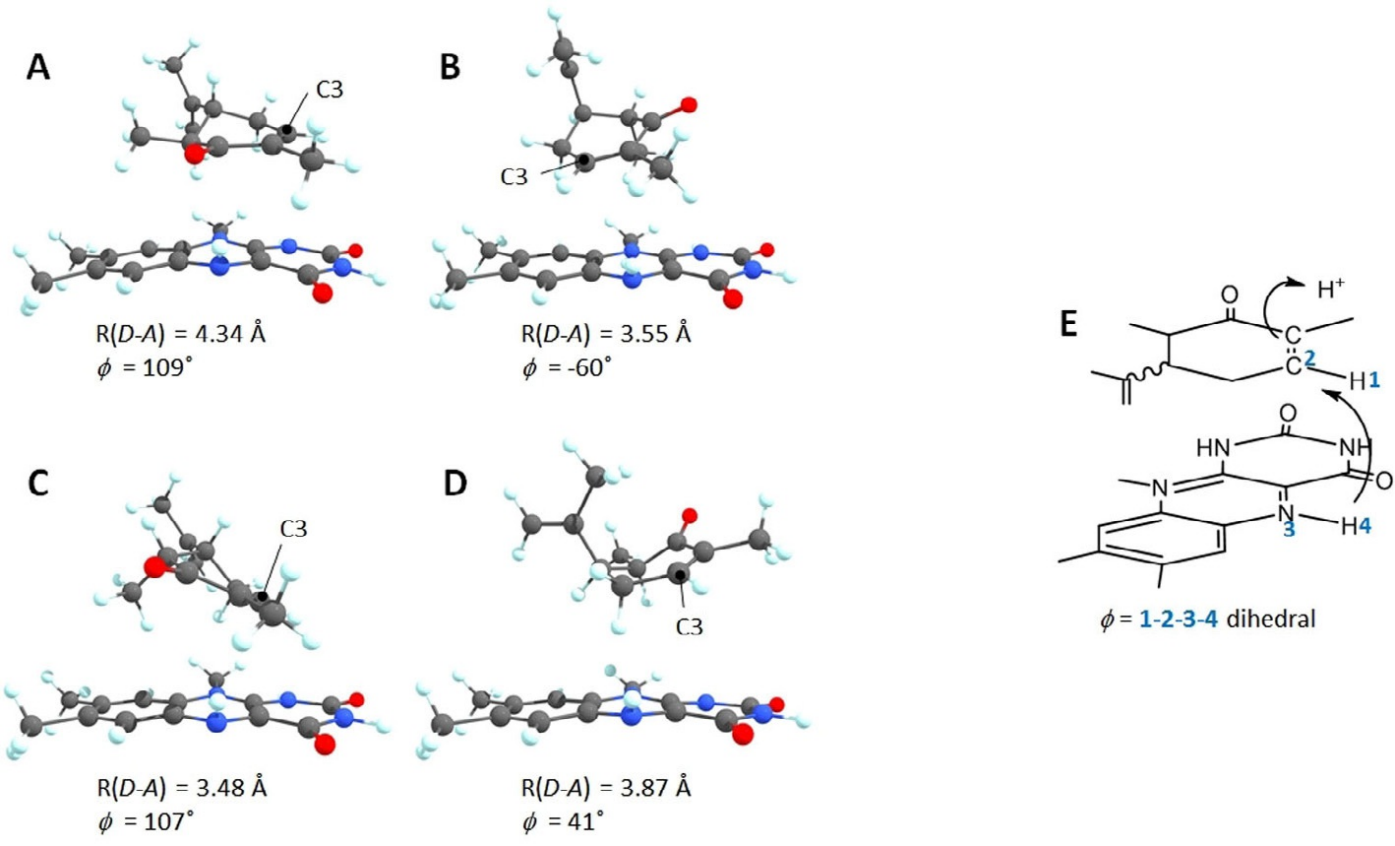
FMNH_2_ and Me carvone from the optimised PETNR active‐site models with (**A**,**B**) 6*S*‐Me carvone and (**C**,**D**) 6*S*‐Me carvone in two possible bound conformations; the donor–acceptor distance for hydride transfer from FMNH_2_ N5 to carvone C3 are listed, as well as the dihedral angle *φ*, which measures how far the transferring hydride sits from the plane ideal for hydride transfer. (**E**) Schematic of the sequential hydride and proton transfers, with definition of the dihedral angle *φ*.

For each substrate, two orientations were modelled, either with the C5 propenyl group facing the flavin or pointing away from it. Because hydride transfer requires that transferring H is in‐plane with the donor and acceptor atoms as well as the accepting *p*‐orbital, we can estimate the degree of rearrangement required by the dihedral angle *φ* in Figure 2 E; thus, conformations A and C in Figure 2 require a significant amount of substrate reorientation for hydride transfer to occur, and we can infer that hydride transfer from FMNH_2_ is more likely for the conformations where the C5‐propenyl group points away from the Flavin (Figure 1 B and D), with proton transfer (either from a water molecule or active site Tyr) to the opposite face of the substrate, which leads to the major observed product. This supports a mechanistic rationale for the conserved selectivity of diastereofacial reduction for the different substrate diastereomeric C6 configurations.

Having identified the diastereo‐differentiated reactivity for C6‐Me carvones, the effect of heteroatom substitution at C6 (rather than Me), while preserving comparable steric effects was explored. This was performed by using both C6 diastereomers with 6‐OH substitution in place of 6‐Me for both (−)‐ and (+)‐carvone backbones (**4** and **5**, and **7** and **8**, respectively; syntheses from Scheme 1). The two (−)‐carvone derived 6‐hydroxyl diastereomers, **4** and **5**, were converted to the corresponding 6‐hydroxydihydrocarvones, **15** and **17**, by OYE2 with moderate yields (Table 1, entries 7–10), but with very high diastereoselectivity in all cases (Scheme 3). PETNR proved a significantly better biocatalyst, affording 66 and 80 % yields of **15** and **17**, respectively, after 2 h, with very high diastereoselectivities. As with the 6‐Me substrates, these were poorer substrates for OYE3 under the same conditions, with trace conversion of **5,** but up to 17 % yield with **4** (Table S7).

**Scheme 3:**
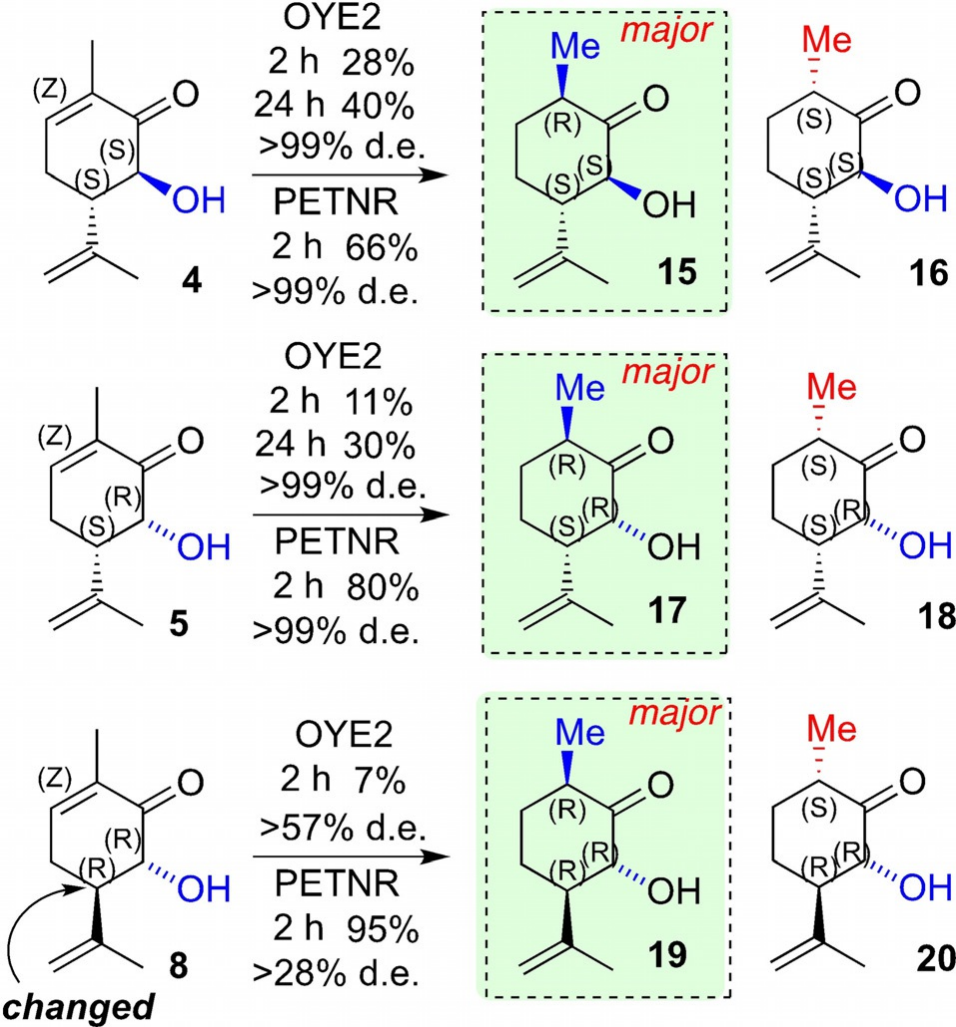
OYE2 and PETNR bioreduction of 6‐OH (−)‐ and (+)‐carvones.

The corresponding 6‐hydroxycarvone diastereoisomers derived from *S*‐(+)‐carvone (**7** and **8**) were poor substrates for OYE2, affording <10 % yields at 2–24 h (Scheme 4). This was similar to PETNR with the (6*S*)‐**7** (5–10 % maximum yields across both enzymes); however, PETNR showed excellent yields of **19** (85–95 %) from (6*R*)‐**8**, but with much reduced *de* (<30 %). (Table 1, Entries 11 and 12).

**Scheme 4:**
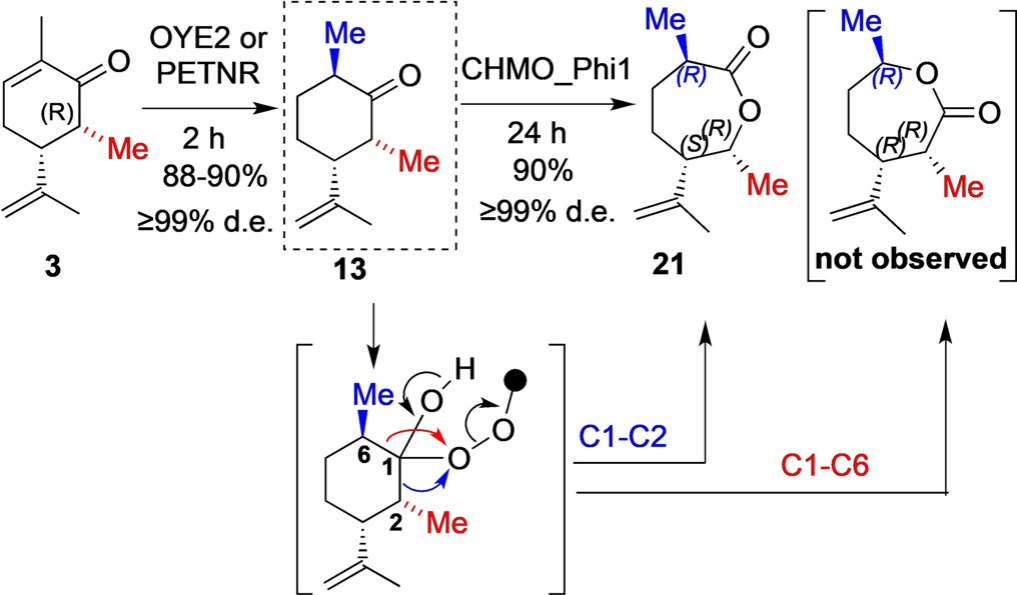
Sequential biocatalysis ene‐reductase‐regiospecific biocatalytic conversion of (−)‐carvone to enantiopure caprolactone derivative **21**.

The synthetic C3‐Me‐(−)‐carvone analogue **10** is a regioisomer of the 6‐Me substrates **2** and **3**, but introducing a methyl at the site of enzymatic conjugate reductive attack. Bioreductions with OYE2 and PETNR led to poor yields (≤10 %), although with high *de* (≥99) in favour of the (2*S*, 3*R*, 6*R*)‐3‐methyldihydrocarvone product.

This suggests that the nature and/or location of an additional substituent on the carvone scaffold has significant impact on bioconversion and selectivity with OYEs. Amongst the (−)‐ and (+)‐6‐OH‐hydroxycarvones **4**, **5**, **7** and **8**, PETNR is the biocatalyst of choice for high yielding and highly diastereoselective bioreductions of either (6*R*)‐ or (6*S*)‐**4** and **5**, providing a practicable biocatalytic route to novel 6‐OH carvones **15** and **17**.

With efficient biosynthesis demonstrated for (2*R*, 5*R*, 6*S*) and (2*R*, 5*R*, 6*R*)‐6‐methyldihydrocarvone isomers **11** and **13** (Scheme 2, Table 1), we sought to evaluate these products as non‐native substrates for Baeyer–Villiger monooxygenase (BVMO) ring expansion reactions. Applications of BVMOs have been attracting attention as an alternative to chemical syntheses, for potentially delivering lactones with improved or changed regioselectivity.^17^

The (2*R*, 5*R*, 6*R*)‐6‐methyldihyrocarvone isomer **13** was completely converted to lactone **21** with apparently complete regioselectivity (≥99 %, SI, GC, Figure S12). However, there was no observed lactone produced from (2*R*, 5*R*, 6*S*)‐6‐methylcarvone **11** (total substrate recovery). This indicates a remarkable diastereoisomer‐selectivity whereby a change of 6‐methyl configuration can largely preclude enzymatic transformation.

Conversion of **13** to **21** provides a highly efficient dual‐biocatalyst process in which a synthetic diastereopure carvone analogue(s) are the best substrate(s) for the ene‐reductase (OYE2 or PETNR) and highly effective substrate for single isomer lactone formation with CHMO. As the carvone derivatives described here contain two similar groups alpha to the carbonyl (compared to one methylene for the natural terpenoids), there are two migration pathways that may compete for any Baeyer–Villiger reaction,^18^ with O‐insertion into either C1−C2 or C1−C6 (see Scheme 4). We investigated whether the substrate diastereo‐configuration would impact efficacy and regiocontrol of the subsequent BVMO reaction by evaluating both 6‐Me dihydrocarvone diastereomers **11** and **13**. Both substrates were therefore screened against CHMO_Phi1 from *Rhodococcus* sp. Phi1.^19^ To further demonstrate the synthetic utility of this dual biocatalytic route, bio‐expansion of (2*R*, 5*R*, 6*R*)‐6‐methyldihydrocarvone **13** was scaled up using 50 mg of substrate. Analytical TLC showed no starting material or any evidence of by‐products after 24 h, and the pure lactone product **21** was obtained with 90 % yield, completing an efficient laboratory scale sequential ene‐reduction‐BV expansion process with complete diastero‐ and regiocontrol across both steps (Scheme 4).

With such highly regioselective expansions and diastereomer‐sensitivity, we wished to evaluate the regio‐isomeric 3‐methyl modified dihydrocarvones through a similar sequential biocatalytic process. However, the poor ene‐reductase outcomes using OYE2 and PETNR for synthetic substrate **10** (vide supra) led us to assess a *chemically reduced* mixture of such 3‐methyldihydrocarvones for enzymatic BV conversion. Chemical reduction with Cu^I^‐catalysed trimethylaluminium provided a mixture of four isomers with a diastereomeric ratio of 1.5:1:1:0.1,^20^ The two 3*R* diastereomers **22** and **23** (about 70–75 % of total) were separated from the two (3*S*)‐**24** and **25**. This allowed evaluation of all four isomers, and of the separate pairs of C3 diastereomers with CHMO_Phi1. Biotransformations were run at 25 °C for 24 h, with NADP^+^/GDH employed as the hydride donor. Product analysis by GC showed complete conversion of (2*S*, 3*R*, 5*R*)‐ and (2*R*, 3*R*, 5*R*)‐**22** and **23** to their corresponding lactones, **26** and **27**, in 98 % yield, and ≥99.9 % *ee* (Scheme 5, Figures S13 and S14) However, no lactones were observed from biooxidation of (2*R*, 3*S*, 5*R*)‐**24** and (2*S*, 3*S*, 5*R*)‐**25** diastereomers. Noting this remarkable diastereomer‐specific (3*S* inactive) behaviour, the biocatalytic oxidation of **22**/**23** was scaled up to 50 mg under the same conditions. No starting materials were evident by TLC after 24 h, and after organic extraction and isolation, the two lactones **26** and **27** were obtained in high yield. Future applications as ROP components may be addressable, comparable to the regioisomeric lactone mixtures obtained from BVase conversion of (+)‐dihydrocarvone.^10^

**Scheme 5:**
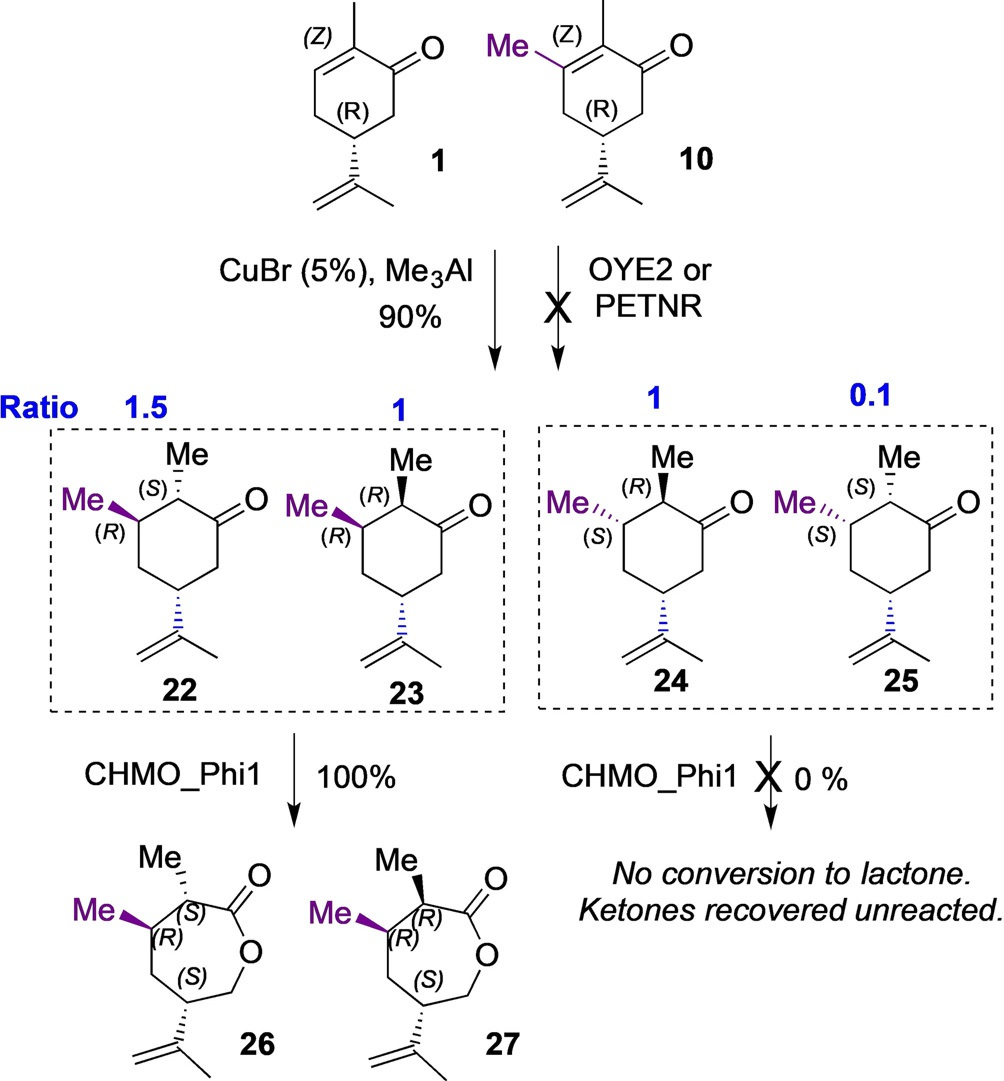
Biocatalytic diastereomer‐discriminating BV of 3‐methyldrohydrocarvones. 25 °C for 24 h, Enzyme (2 μm), Substrate (5 mm) pH 7.0 of 50 mm Tris⋅HCl buffer solution, NADP^+^ (15 μm), GDH (10 U), Glucose (15 mm), 25 °C at 130 rpm for 24 h.

In conclusion, evaluation of synthetically modified carvone scaffolds (6‐Me, 6‐OH or 3‐Me) using three OYEs (PETNR, OYE2 and OYE3) have identified that OYE2 and PETNR are efficient ene‐reductases of 6‐Me substituted carvones, with the configuration at C6 a major determinant of substrate conversion. The 6‐OH substituted substrates **4** and **5** showed significant differences between OYE2 and PETNR, unlike their C6‐methyl analogues, with PETNR being a significantly better biocatalyst. The best substrates for overall yield and high *de* were (6*R*)‐Me‐(−)‐carvone **3** and either diastereoisomer of 6‐OH‐(−)‐carvone, **4** and **5** and this work provides a viable biocatalyst route to enantiopure 6‐substituted dihydrocarvones **13**, **15** and **17**.

Furthermore, homochiral intermediate **13** undergoes a highly efficient biocatalytic Baeyer–Villiger reaction with essentially complete regiocontrol to afford chiral lactone **23**. Whilst 3‐methylcarvone is shown to be a poor substrate for ene‐reductase, chemically synthesised 3‐methylated dihydrocarvones are shown to be excellent substrates for BVMOs, identifying a near complete selectivity based on the configuration of the additional methyl not present in natural dihydrocarvone. The (3*R*)‐diastereomers **22** and **23** are completely converted into new chiral lactones, **26** and **27**, whilst the (3*S*)‐diastereomers **24** and **25** are not enzyme substrates. Biocatalytic routes were also shown to be viable on a preparative synthetic scale. These enzymatic reactions provide insight defining scope of diastereomer control of enzyme selectivity for new synthetic substrates, both with respect to selectivity by ene‐reductases for modified carvones, but also importantly for the selectivity of enzymatic BVMO ring expansions. This provides a practical route to several chiral derivatives through synthetic‐enzymatic processes, and a convenient chem‐enz‐enz route to enantiopure new caprolactone **21**, and to 6*R* configuration‐specific diastereomeric mixture of the caprolactones **26** and **27**, regioisomeric with **21**, which may all be of value as ROP components.

## Conflict of interest

The authors declare no conflict of interest.

## Supporting information

As a service to our authors and readers, this journal provides supporting information supplied by the authors. Such materials are peer reviewed and may be re‐organized for online delivery, but are not copy‐edited or typeset. Technical support issues arising from supporting information (other than missing files) should be addressed to the authors.

Supplementary
