# Supplementary material for: C3 and C6 Modification‐Specific OYE Biotransformations of Synthetic Carvones and Sequential BVMO Chemoenzymatic Synthesis of Chiral Caprolactones
Source: Chemistry. 2019 Jan 15;25(12):2983–8. doi: 10.1002/chem.201805219 (PMC6468273; doi:10.1002/chem.201805219)
Supplement: Supplementary file 1 — Supplementary [file CHEM-25-2983-s001.pdf]

CHEMISTRY
A European Journal

Supporting Information

C3 and C6 Modification-Specific OYE Biotransformations of Synthetic Carvones and Sequential BVMO Chemoenzymatic Synthesis of Chiral Caprolactones**

Issa S. Issa,[a] Helen S. Toogood,[b] Linus O. Johannissen,[b] James Raftery,[a] Nigel S. Scrutton,[b] and John M. Gardiner*[a]

chem_201805219_sm_miscellaneous_information.pdf

Author Contributions

I.I. Data curation: Lead; Formal analysis: Lead; Investigation: Equal; Methodology: Equal; Project administration: Supporting; Validation: Lead; Writing – original draft: Supporting; Writing – review & editing: Supporting
H.T. Supervision: Supporting; Validation: Supporting; Writing – review & editing: Supporting; advise and training: Supporting
L.J. Data curation: Supporting; Investigation: Supporting; Visualization: Supporting; Writing – original draft: Supporting; Writing – review & editing: Supporting; Modelling: Lead
J.R. X-ray data: Lead
N.S. Conceptualization: Equal; Funding acquisition: Equal; Supervision: Supporting; Writing – review & editing: Supporting; PI for biocat funding: Lead
J.G. Conceptualization: Lead; Funding acquisition: Equal; Methodology: Equal; Project administration: Lead; Resources: Lead; Supervision: Lead; Validation: Lead; Writing – original draft: Equal; Writing – review & editing: Lead.

Supporting Information

C3 and C6 Modification-specific OYE biotransformations of synthetic carvones and sequential BVO chemoenzymatic synthesis of chiral caprolactones

Issa S. Issa,[a] Helen S. Toogood,[b] Linus O. Johannissen,[b] James Raftery,[a] Nigel S. Scrutton[b] and John M. Gardiner[a]*

[a]Manchester Institute of Biotechnology and School of Chemistry, 131 Princess Street, The University of Manchester, Manchester M1 7DN, UK

[b]BBSRC/EPSRC Manchester Synthetic Biology Research Centre for Fine and Specialty Chemicals (SYNBIOCHEM), Manchester Institute of Biotechnology, School of Chemistry, Faculty of Science and Engineering, University of Manchester, 131 Princess Street, Manchester M1 7DN, U.K.

KEYWORDS (Word Style "BG_Keywords"). OYEs; PETNR; Baeyer-Villagerase; Carvone

gardiner@manchester.ac.uk

1 Experimental methods

(5*R*,6*S*)-(+)-5-Isopropenyl-2,6-dimethylcyclohexane-2-enone, **2**, and (5*R*,6*R*)-(+)-5-Isopropenyl-2, 6-dimethylcyclohexane-2-enone, **3**.............p3-4

5*S*,6*R*)-6-Hydroxy-2-methyl-5- (prop-1-en-2-yl) cyclohex-2-en-1-one, **4**, and (5*S*,6*S*)-6-Hydroxy-2-methyl-5- (prop-1-en-2-yl) cyclohex-2-en-1-one, **5**..p5-6

(5*R*,6*R*)-6-Hydroxy-2-methyl-5-(prop-1-en-2-yl)cyclohex-2-en-1-one, **7**, and (5*R*,6*S*)-6-Hydroxy-2-methyl-5-(prop-1-en-2-yl)cyclohex-2-en-1-one, **8**.........p7

(*R*)-2,3-Dimethyl-5-(prop-1-en-2-yl)cyclohex-2-en-1-one, **10**..........................p8

(2*S*,3*R*,6*R*)-2,6-Dimethyl-3- (prop-1-en-2-yl)cyclohexan-1-one, **11**
(2*S*,3*R*,6*S*)-2,6-Dimethyl-3-(prop-1-en-2-yl)cyclohexan-1-one, **12**
(2*R*,3*R*,6*R*)-2,6-Dimethyl-3-(prop-1-en-2-yl)cyclohexan-1-one, **13**, and
(2*R*,3*R*,6*S*)-2,6-Dimethyl-3-(prop-1-en-2-yl)cyclohexan-1-one, **14**..................p9-11

Scale up bioreduction of (5*R*,6*R*)-6-methylcarvone **3** via PETNR to **13**............p11

(2S,3S,6R)-2-Hydroxy-6-methyl-3-(prop-1-en-2-yl)cyclohexan-1-one, **15**, and (2S,3S,6S)-2-Hydroxy-6-methyl-3-(prop-1-en-2-yl)cyclohexan-1-one, **16** (mixture of isomers)... ....p11-12

(2R,3S,6R)-2-Hydroxy-6-methyl-3-(prop-1-en-2-yl)cyclohexan-1-one, **17**, and (2R,3S,6S)-2-Hydroxy-6-methyl-3-(prop-1-en-2-yl)cyclohexan-1-one, **18** (mixture of isomers)... ....p12-13

Scale up bioreduction of (5S,6R)-6-hydroxycarvone p13

(2R,3R,6S)-2-Hydroxy-6-methyl-3-(prop-1-en-2-yl)cyclohexan-1-one, **20**, and (2R,3R,6R)-2-Hydroxy-6-methyl-3-(prop-1-en-2-yl)cyclohexan-1-one, **19** (mixture of isomers) p14

(2S,3R,5R)-2,3-Dimethyl-5-(prop-1-en-2-yl)cyclohexan-1-one, **22**

(2R,3R,5R)-2,3-Dimethyl-5-(prop-1-en-2-yl)cyclohexan-1-one, **23**
(2R,3S,5R)-2,3-Dimethyl-5-(prop-1-en-2-yl)cyclohexan-1-one, **24**, and
(2S,3S,5R)-2,3-Dimethyl-5-(prop-1-en-2-yl)cyclohexan-1-one, **25** (mixture).p15-16

General BVMO protocol and Scale up of BV oxidation of (2R,3R,6R)-2,6-dimethyl-3-(prop-1-en-2-yl)cyclohexan-1-one 21 by CHMOs and (3R,6S,7R)-3,7-Dimethyl-4-(prop-1-en-2-yl)oxepan-2-one, **21**………………………………………………………p16-17

Scale up of BV oxidation of two isomers mixture of 3-methyldihydrocarvone 22 and 23 by CHMOs: (3R,4R,6S)-3,4-Dimethyl-6-(prop-1-en-2-yl)oxepan-2-one, **26**, and (3S,4R,6S)-3,4-Dimethyl-6-(prop-1-en-2-yl)oxepan-2-one, **27** (mixture of normal and abnormal lactones) ………………………………………………………… p17-18

2 Spectral data (^{1}H / ^{13}C NMR / COSY)

(5*R*,6*S*)-(+)-5-Isopropenyl-2,6-dimethylcyclohexane-2-enone, 2………………….p19-21

(5*R*,6*R*)-(+)-5-Isopropenyl-2, 6-dimethylcyclohexane-2-enone, 3 ……………..p22-24

(5*S*,6*R*)-6-Hydroxy-2-methyl-5- (prop-1-en-2-yl) cyclohex-2-en-1-one, **4**…..p25-27

(5*S*,6*S*)-6-Hydroxy-2-methyl-5- (prop-1-en-2-yl) cyclohex-2-en-1-one, **5** ….p28-30

(2*S*,3*R*,6*R*)-2,6-Dimethyl-3- (prop-1-en-2-yl)cyclohexan-1-one, **11**……………p31-33

(2*S*,3*R*,6*S*)-2,6-Dimethyl-3-(prop-1-en-2-yl)cyclohexan-1-one, **12** ……………..p34-36

(2*R*,3*R*,6*R*)-2,6-Dimethyl-3-(prop-1-en-2-yl)cyclohexan-1-one, **13**……………p37-39

(2*R*,3*R*,6*S*)-2,6-Dimethyl-3-(prop-1-en-2-yl)cyclohexan-1-one, **14**……………..p40-42

(2*S*,3*S*,6*R*)-2-Hydroxy-6-methyl-3-(prop-1-en-2-yl)cyclohexan-1-one, **15**………...p43-45

(2*R*,3*S*,6*R*)-2-Hydroxy-6-methyl-3-(prop-1-en-2-yl)cyclohexan-1-one, **17**………..p46-48

(2*R*,3*R*,6*S*)-2-Hydroxy-6-methyl-3-(prop-1-en-2-yl)cyclohexan-1-one, **20**…………p49-51

(*R*)-2,3-Dimethyl-5-(prop-1-en-2-yl)cyclohex-2-en-1-one, **10**……………………..p53-54

(2*S*,3*R*,5*R*)-2,3-Dimethyl-5-(prop-1-en-2-yl)cyclohexan-1-one, **22**, (2*R*,3*R*,5*R*)-2,3-Dimethyl-5-(prop-1-en-2-yl)cyclohexan-1-one, **23**, (2*R*,3*S*,5*R*)-2,3-Dimethyl-5-(prop-1-en-2-yl)cyclohexan-1-one, **24**, and (2S,3S,5R)-2,3-Dimethyl-5-(prop-1-en-2-yl)cyclohexan-1-one, **25** (mixture) ………………………………………………………p55-57

(3*R*,6*S*,7*R*)-3,7-Dimethyl-4-(prop-1-en-2-yl)oxepan-2-one, **21** ……………………….p58-60

(3*R*,4*R*,6*S*)-3,4-Dimethyl-6-(prop-1-en-2-yl)oxepan-2-one, **26**, and (3S,4R,6S)-3,4-Dimethyl-6-(prop-1-en-2-yl)oxepan-2-one, 27 (mixture of lactones) ………………..p61-63

(5*R*,6*R*)-6-Hydroxy-2-methyl-5-(prop-1-en-2-yl)cyclohex-2-en-1-one, **7**……………p64-66

(5*R*,6*S*)-6-Hydroxy-2-methyl-5-(prop-1-en-2-yl)cyclohex-2-en-1-one, **8**……………p67-29

3 Enzymology p70-90

4 Modelling p92

5 X-ray structure p92

6 References p93

Supporting Information

1 Experimental methods,

(5*R*,6*S*)-(+)-5-Isopropenyl-2,6-dimethylcyclohexane-2-enone, 2, and (5*R*,6*R*)-(+)-5-Isopropenyl-2, 6-dimethylcyclohexane-2-enone, 3 [1]

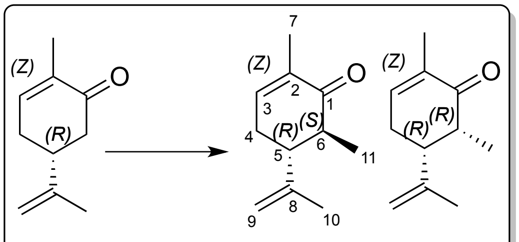

1 2 3

A hexane solution of ^nBuLi (59.44 mL, 83.20 mmol, 1.4 M) was added dropwise to a cold solution (−10 °C under nitrogen gas) of DIPA (11.7 mL, 83.20 mmol) in anhydrous THF (60 mL) over a period of 10 min. The mixture was continuously stirred for 10 min to allow LDA formation. (−)-Carvone (9.6 g, 63.8 mmol) in anhydrous THF (90 mL) was slowly added and the mixture stirred for 45 min at the same temperature. The lithium enolate was then reacted with an excess of methyl iodide CH_3I (20 mL, 321.3 mmol), and the reaction mixture allowed stirring overnight at RT. It was extracted with diethyl ether (3 x 30 mL), acidified with 3M aq. HCl and then washed with $NaHCO_3$ and brine. The combined organic extracts were dried ($MgSO_4$), filtered, and solvent removed *in vacuo*. The residue was chromatographed (eluent: EtOAc:hexane 40:1–20:1) to give a 3:2 epimeric mixture of 6-methyl carvone (9.63 g, 85% yield) as a pale yellow oil.

To a diastereomeric mixture of 6-methylcarvone (1 g, 6.09 mmol) in CH_2Cl_2 (10 mL), was added DBU (0.9 mL, 6.09 mmol) and the reaction mixture was allowed to stir for 24 h at room temperature. The reaction mixture was washed with 1 M HCl (1 x 30 mL), water (1 x 30 mL) and brine (1 x 30 mL). The organic layer was dried ($MgSO_4$), solvents removed *in vacuo* and column chromatography eluting with hexane:EtOAc (40:1–50:1) afforded a 3:1 epimeric mixture of 6-methylcarvone. Partial separation of diastereomers (250 mg), providing major diastereomer **2** (80 mg, 32%, R_f 0.54) as white crystals, and, minor diastereomer **3**, (20 mg, 8%, R_f 0.52) as a colourless oil, along with 150 mg of a diastereomeric mixture (60%).

The mixture of isomers (200 mg) was also recrystallized using hexane (−10 °C) to yield white crystals of **2** (76 mg, 38%).

(6*S*)-Diastereomer (2): ^{1}H NMR (400 MHz, $CDCl_3$) δ 6.69–6.66 (m, 1H, $C\underline{H}=CCH_3$, H-3), 4.81–4.79 (m, 2H, $C\underline{H_2}=C$, H-9), 2.50–2.39 (m, 2H, $CH_2CHC\underline{H}$ and $CH_2C\underline{H}CH$, H-6 and H-5), 2.35 (dt, *J* = 13.0, 6.5 Hz, 1H, $C\underline{H_2}CHCH$, H-4), 2.29–2.21 (m, 1H, $C\underline{H_2}CHCH$, H-4), 1.76 (dt, *J* = 2.5, 1.3 Hz, 3H, $CH_2=CC\underline{H_3}$), 1.69 (dd, *J* = 1.4, 0.8 Hz, 3H, $COCC\underline{H_3}$), 1.03 (d, *J* = 6.6 Hz, 3H, $C\underline{H_3}CHC=O$).

^{13}C NMR (100 MHz, $CDCl_3$) δ 201.9 ($\underline{C}=O$), 145.8 ($CH_3\underline{C}=CH_2$, C-8), 143.5 ($CH_3C=\underline{C}H$, C-3), 134.9 ($CH_3\underline{C}=CH$, C-2), 113.3 ($CH_3C=\underline{C}H_2$, C-9), 50.7 ($CH_2\underline{C}HCH$, C-5), 44.4 ($CH_2CH\underline{C}H$, C-6), 31.3 ($\underline{C}H_2CHCH$, C-4), 18.3 ($CH_2=C\underline{C}H_3$, C-10), 16.3 ($COC\underline{C}H_3$, C-7), 12.7 ($\underline{C}H_3CHC=O$, C-11).

MS (ES) *m/z* 165 (MH^+, 100%); oil; Anal. Calc. for $C_{11}H_{16}O$, Expected C, 80.4; H, 9.8; Found C, 80.1; H, 10.0; IR ν_{max} 2986, 2971, 2924, 1664 (C=O), 1444, 1365, 1290, 1134, 1045. 951, 846 cm^{-1}; ; $[\alpha]_D$ +12.9° (c 1.7, CH_2Cl_2, 24 °C); m.p 37–38 °C.

(6*R*)-Diastereomer (3): ^{1}H NMR (400 MHz, $CDCl_3$) δ 6.72 (ddt, *J* = 5.7, 2.7, 1.4 Hz, 1H, $C\underline{H}=CCH_3$, H-3), 4.91 (dq, *J* = 2.7, 1.4 Hz, 1H, $C\underline{H_2}=C$, H-9), 4.72 (s, 1H, $C\underline{H_2}=C$, H-9), 2.73–2.63 (m, 2H, $CH_2CHC\underline{H}$ and $CH_2C\underline{H}CH$, H-6 and H-5), 2.50–2.40 (m, 1H, $C\underline{H_2}CHCH$, H-4), 2.32–2.24 (m, 1H, $C\underline{H_2}CHCH$, H-4), 1.77 (dt, *J* = 2.5, 1.3 Hz, 3H, $CH_2=CC\underline{H_3}$), 1.69 (dt, *J* = 1.3, 0.7 Hz 3H, $COCC\underline{H_3}$), 0.90 (d, *J* = 7.2 Hz, 3H, $C\underline{H_3}CHC=O$).

^{13}C NMR (100 MHz; $CDCl_3$) δ 203.7 (C=O), 145.0 ($CH_3\underline{C}=CH_2$, C-8), 144.2 ($CH_3C=\underline{C}H$, C-3), 133.8 ($CH_3\underline{C}=CH$, C-2), 111.6 ($CH_3C=\underline{C}H_2$, C-9), 44.9 ($CH_2\underline{C}HCH$, C-5), 43.1 ($CH_2CH\underline{C}H$, C-6), 26.4 ($\underline{C}H_2CHCH$, C-4), 22.0 ($CH_2=C\underline{C}H_3$, C-10), 16.1 ($COC\underline{C}H_3$, C-7), 10.6 ($\underline{C}H_3CHC=O$, C-11).

MS (ES) *m/z* 165 (MH^+, 80%); IR ν_{max} 2969, 2923, 1667 (C=O), 1450, 1365, 1249, 1130, 1061, 989, 888 cm^{-1}; oil; IR ν_{max} 2969, 2923, 1667 (C=O), 1450, 1365, 1249, 1130, 1061, 989, 888 cm^{-1}; $[\alpha]_D$ +28.5° (c 0.7, CH_2Cl_2, 24 °C).

5*S*,6*R*)-6-Hydroxy-2-methyl-5- (prop-1-en-2-yl) cyclohex-2-en-1-one, 4, and (5*S*,6*S*)-6-Hydroxy-2-methyl-5- (prop-1-en-2-yl) cyclohex-2-en-1-one, 5 [2]

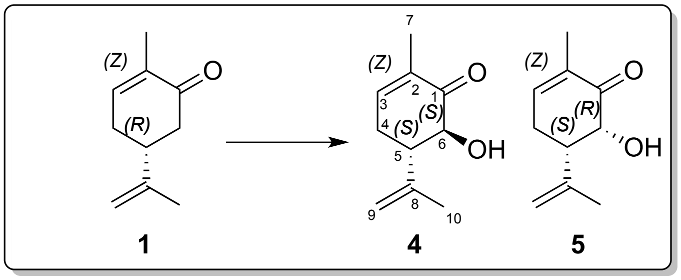

DIPA (5.7 mL, 40.4 mmol) was purged to dry THF (50 mL) under N_2 gas, and a hexane solution of *n*-BuLi (19.4 mL, 40.6 mmol, 2.09 M) was slowly dropped at 0 °C. The reaction mixture was allowed to stir for further 2 h, and the vessel was cooled to −78 °C. Thence, *R*-(−)-carvone (5 g, 33.3 mmol) in fresh THF (50 mL) was dropwise added via cannula, and the reaction aged for 30 min. The reaction mixture was quenched with TMSCl (8.3 mL, 65.7 mmol in 50 mL THF), and the ice-acetone removed to allow the reaction to stir for an additional 60 min at ambient temperature. The solvent was removed *in vacuo*, and the concentrated organic layer was added to Celite®. The slurry was taken up with pentane (200 mL), and more pentane was added to wash the precipitate. The organic layer was washed with $Na_2S_2O_3$ (2 x 30 mL), saturated $NaHCO_3$ (2 x 30 mL) and water (2 x 50 mL), dried over ($MgSO_4$) and concentrated to give silylenol ether (6.80 g, 92%) as a yellow oil.

Dry DCM was added to a round bottomed flask (70 mL) containing silylenol ether (6.50 g, 27.3 mmol) under nitrogen atmosphere, and *m*-CPBA (7 g, 40.6 mmol) in dry DCM (150 mL) was dropwise transferred from second round flask at 0 °C. The reaction mixture was allowed to warm at RT, and stirred for further 60 min. Then, the mixture was washed with NaS_2O_3 (1 x 30 mL), and neutralised to pH = 7.0 via adding saturated $NaHCO_3$. The solid was filtered off by through a pad of Celite®, and the filtrate was concentrated under vacuum to yield a mixture of silylenol ether diastereomers (6.05 g, 76%) as a yellowish-green oil. HCl (50 mL, 1.5 M) was added to a mixture of silylenol ether (25.38 mmol), and allowed to stir overnight at RT. The mixture was neutralised with NaOH (0.1 M), and washed with water (3 x 20 mL), brine (3 x 20 mL), dried over ($MgSO_4$), and solvent removed *in vacuo*. The mixture was purified with flash column chromatography using

hex:EtOAc (4:1) to afford separable diastereomers of *anti* isomer of 6-hydroxycarvone **4** (2.49 g, 45%) and *syn* **5** (0.7 g, 13%) as a yellow oil.

(6*S*)-Diastereomer (4): ^{1}H NMR (400 MHz, $CDCl_3$) δ 6.75–6.71 (m, 1H, C=C$\underline{H}$, H-3), 4.92–4.89 (m, 2H, C$\underline{H}_2$=C, 2 x H-9), 4.13 (d, 1H, *J* = 12.7 Hz, C$\underline{H}$OH, H-6), 3.78 (s, 1H, O$\underline{H}$), 2.67 (ddd, *J* = 12.7, 11.0, 5.1 Hz, 1H, CH_2C$\underline{H}$CH, H-5), 2.51–2.41 (m, 1H, C$\underline{H}_2$CHCH, H-4) 2.39–2.31 (m, 1H, C$\underline{H}_2$CHCH, H-4), 1.82–1.81 (m, 6H, 2 x CH_3).

^{13}C NMR (100 MHz, $CDCl_3$) δ 200.7 (C=O), 145.8 ($\underline{C}$=CH_2, C-8), 144.3 (C=$\underline{C}$H, C-3), 133.1 ($\underline{C}$=CH, C-2), 113.7 (C=$\underline{C}H_2$, C-9), 74.5 ($\underline{C}$HOH, C-9), 51.2 ($CH_2$$\underline{C}$HCH, C-5), 30.8 ($\underline{C}H_2$CHCH, C-4), 18.9 ($\underline{C}H_3$), 15.5 (C$\underline{C}H_3$).

HRMS (ES) *m/z* for $C_{10}H_{14}O_2Na$, $[MNa]^+$, (Calc.: 189.0891, Found: 189.0882); oil; IR ν_{max} 3476 OH), 2971, 2922, 2892, 1670 (C=O), 1646, 1434, 1371, 1286, 1136, 931 cm^{-1}, $[\alpha]_D$ −33° (c 1.4, CH_2Cl_2, 24 °C).

(6*R*)-Diastereomer (5): ^{1}H NMR (400 MHz, $CDCl_3$) δ 6.65 (ddt, *J* = 4.1, 2.7, 1.4 Hz, 1H, C=C$\underline{H}$, H-3), 4.83 (quint, *J* = 1.5 Hz, 1H, C$\underline{H}_2$=C, H-9), 4.69–4.67 (m, 1H, C$\underline{H}_2$=C, H-9), 4.40 (d, 1H, *J* = 5.9 Hz, C$\underline{H}$OH, H-6), 3.60 (s, 1H, O$\underline{H}$), 3.17-3.13 (m, 1H, C$\underline{H}$CHOH, H-5), 2.74–2.65 (m, Hz, 1H, C$\underline{H}_2$CHCH, H-4), 2.55–2.47 (m, 1H, C$\underline{H}_2$CHCH, H-4) 1.80 (dt, *J* = 2.7, 1.4 Hz, 3H, COCC$\underline{H}_3$), 1.66–1.65 (m, 3H, CH_2=CC$\underline{H}_3$).

^{13}C NMR (100 MHz, $CDCl_3$) δ 199.9 (C=O), 144.2 ($\underline{C}$=CH_2, C-8), 143.1 (C=$\underline{C}$H, C-3), 133.8 ($\underline{C}$=CH, C-2), 113.9 (C=$\underline{C}H_2$, C-9), 74.7 ($\underline{C}$HOH, C-6), 47.1 ($CH_2$$\underline{C}$HCH, C-5), 29.8 ($\underline{C}H_2$CHCH, C-4), 23.2 ($\underline{C}H_3$C=$CH_2$, C-10), 15.4 (COC$\underline{C}H_3$, C-7).

HRMS (ES) *m/z* for $C_{10}H_{14}O_2Na$, $[MNa]^+$, (Calc.: 189.0891, Found: 189.0885); oil; IR ν_{max} 3463 OH), 2964, 2922, 1711(C=O), 1670, 1433, 1360, 1256, 1132, 870 cm^{-1}, $[\alpha]_D$ −10.0° (c 0.88, CH_2Cl_2, 24 °C).

(5*R*,6*R*)-6-Hydroxy-2-methyl-5-(prop-1-en-2-yl)cyclohex-2-en-1-one, 7, and (5*R*,6*S*)-6-Hydroxy-2-methyl-5-(prop-1-en-2-yl)cyclohex-2-en-1-one, 8 [3]

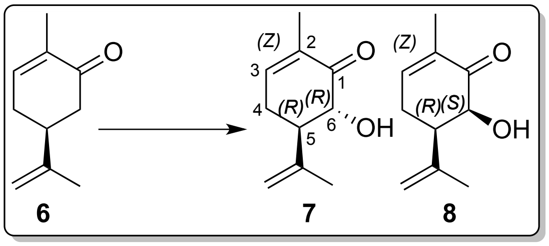

The title compounds were prepared from *S*-(+)-carvone **6** according to the procedure used for **4** and **5**, to afford *anti* **7** (1.88 g, 34%) and *syn* **8** (0.51 g, 9.2%) isomers as a colourless oil.

(6*R*)-Diastereomer (7): ^{1}H NMR (400 MHz*;* $CDCl_3$) δ 6.76–6.74 (m, 1H, C=CH, H-3), 4.94–4.91 m*,* 2H, CH_2=C, 2 x H-9), 4.15 (dd, *J* = 12.7, 1.9 Hz*,* 1H, CHOH, H-6), 3.79 (d, *J* = 1.8 Hz*,* 1H, OH), 2.69 (ddd, *J* = 12.7, 11.0, 5.1 Hz*,* 1H, CH_2CHCH, H-5), 2.52–2.43 (m, 1H, CH_2CHCH, H-4), 2.41–2.33 (m, 1H, CH_2CHCH, H-4), 1.84–1.83 (m, 6H, 2 x CH_3).

^{13}C NMR (100 MHz*,* $CDCl_3$) δ 200.7 (C=O), 145.8 (C=CH_2, C-8), 144.3 (C=CH, C-3), 133.2 (C=CH_2, C-2), 113.7 (C=CH_2, C-9), 74.5 (CHOH, C-6), 51.2 (CH_2CHCH, C-5), 30.8 (CH_2CHCH, C-4), 18.9 (CH_3), 15.5 (CH_3).

MS (ES) *m/z* 189 (MNa^+, 90%), 167 (MH^+, 70%)**;** oil; IR ν_{max} 3477 OH), 2971, 2892, 1724 (C=O), 1670, 1434, 1371, 1242, 1136, 891 cm^{-1}; $[\alpha]_D$ +28 ° (c 0.50, CH_2Cl_2, 24 °C).

(6*S*)-Diastereomer (8): ^{1}H NMR (400 MHz*;* $CDCl_3$) δ 6.67–6.66 (m*,* 1H, 1H, C=CH, H-3), 4.86 (quint, *J* = 1.5 Hz*,* 1H, CH_2=C, H-9), 4.71–4.70 (m, 1H, CH_2=C, H-9), 4.43 (dd, *J* = 6.0, 2.5 Hz*,* 1H, CHOH, H-6), 3.60 (d, *J* = 2.5 Hz*,* 1H, OH), 3.21–3.18 (m, 1H, CH_2CHCH, H-5), 2.78–2.69 (m, 1H, CH_2CHCH, H-4), 2.57–2.50 (m, 1H, CH_2CHCH, H-4), 1.83 (dt, *J* = 2.7, 1.4 Hz*,* 3H, COCCH_3), 1.69 (dd, *J* = 1.3, 0.7 Hz*,* 3H, CH_3C=CH_2).

^{13}C NMR (100 MHz*,* $CDCl_3$) δ 200.0 (C=O), 144.2 (C=CH_2, C-8), 143.2 (C=CH, C-3), 133.9 (C=CH, C-2), 114.0 (C=CH_2, C-9), 74.8 (CHOH, C-6), 47.1 (CH_2CHCH, C-5), 29.9 (CH_2CHCH, C-4), 23.2 (CH_3C=CH_2, C-10), 15.4 (COCCH_3, C-7).

MS (ES) *m/z* 189 (MNa^+, 80%), 167 (MH^+, 60%); oil; IR ν_{max} 3477 (OH), 2971, 2892, 1724 (C=O), 1670, 1434, 1371, 1242, 1136, 891 cm^{-1}; $[\alpha]_D$ −90 ° (c 0.2, CH_2Cl_2, 24 °C).

(*R*)-2,3-Dimethyl-5-(prop-1-en-2-yl)cyclohex-2-en-1-one, 10 [4]

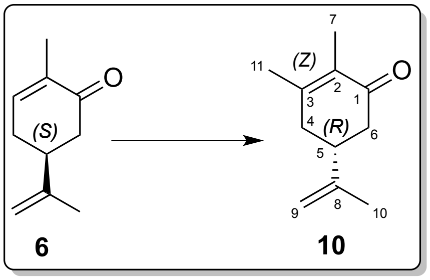

Methylmagnesium iodide (2.9 mL, 3.0 M, 1.3 eq.) was added to diethyl ether (10 mL) in round bottomed flask (250 mL) under N_2 atmosphere. Then, *S*-(+)-carvone **6** (1 g, 6.65 mmol) in dry ether (7 mL) was slowly added over 30 min at −5 °C, and the colour of mixture changed from brown to grey. The reaction was allowed to warm gradually, and stirred for further 3 h at room temperature. The mixture was quenched with saturated NH_4Cl (20 mL), extracted with diethyl ether (100 mL), and dried over ($MgSO_4$). The solvent was removed under reduced pressure to afford alcohol intermediate product. A DCM solution of the alcohol was added to an orange suspension of PCC (2.5 g, 11.7 mmol, 1.8 eq.) and silica gel (2.5 g) in dry DCM (10 mL). The colour of the reaction mixture was altered to dark brown. The reaction mixture was allowed to stir for 3 h at ambient temperature, then, filtered off via silica gel using DCM as eluent, and solvents removed *in vacuo*. The product was purified via flash column chromatography using hex:EtOAc (40:1), to give 3-methylcarvone (1.01 g, 84%) as a yellow oil.

^{1}H NMR (400 MHz, $CDCl_3$) δ 4.79– 4.77 (m, 1H, C=C$\underline{H}_2$, H-9), 4.74–4.73 (m, 1H, C=C$\underline{H}_2$, H-9), 2.66–2.58 (m, 1H, CH_2CHC$\underline{H}_2$, H-6), 2.56–2.51 (m, 1H, CH_2C$\underline{H}$$CH_2$, H-5), 2.37–2.32 (m, 2H, C$\underline{H}_2$CHCH_2, 2 x H-4), 2.31–2.24 (m, 1H, CH_2CHC$\underline{H}_2$, H-6), 1.95 (q, *J* = 0.8 Hz, 3H, 3 x H-7, C$\underline{H}_3$C=CCH_3), 1.76–1.75 (m, 3H, 3 x H-11, CH_3C=C$C\underline{H}_3$), 1.74–1.73 (m, 3H, CH_2=C$C\underline{H}_3$).

^{13}C NMR (100 MHz; $CDCl_3$) δ 199.1 (C=O), 154.3 ($CH_3$$\underline{C}$=C$CH_3$, C-3), 146.9 ($CH_3$C=$\underline{C}$$CH_3$, C-2), 130.8 ($\underline{C}$=$CH_2$, C-8), 110.3 (C=$\underline{C}H_2$, C-9), 42.5 ($CH_2CH\underline{C}H_2$, C-6), 41.4 ($CH_2$$\underline{C}HCH_2$, C-5), 38.1 ($\underline{C}H_2CHCH_2$, C-4), 21.7 ($\underline{C}H_3$C=C$CH_3$, C-7), 20.6 ($CH_3$, CH_3C=C$\underline{C}H_3$, C-11), 10.9 ($\underline{C}H_3$C=CH_2, C-10).

MS (ES) *m/z* 165 (MH^+, 100%); colourless oil; Anal. Calc. for $C_{11}H_{16}O$, Expected C, 80.4; H, 9.8; Found C, 79.3; H, 10.0; IR ν_{max} 2955, 2932, 2917, 1661 (C=O), 1433, 1376, 1257, 1142, 108, 889 cm^{-1}; lit $[\alpha]_D$ −100.6 °, $[\alpha]_D$ −100.4 ° (c 0.56, CH_2Cl_2, 24 °C).

(2*S*,3*R*,6*R*)-2,6-Dimethyl-3- (prop-1-en-2-yl)cyclohexan-1-one, 11
(2*S*,3*R*,6*S*)-2,6-Dimethyl-3-(prop-1-en-2-yl)cyclohexan-1-one, 12
(2*R*,3*R*,6*R*)-2,6-Dimethyl-3-(prop-1-en-2-yl)cyclohexan-1-one, 13, and
(2*R*,3*R*,6*S*)-2,6-Dimethyl-3-(prop-1-en-2-yl)cyclohexan-1-one, 14 [5]

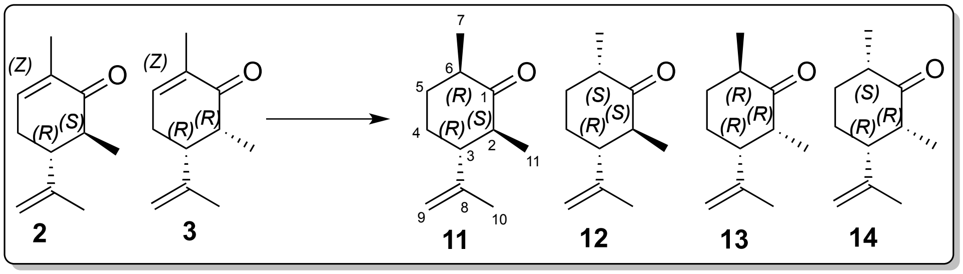

Sodium dithionite $Na_2S_2O_4$ (5.05 g, 29 mmol, 9 eq.) was added in one-portion to a two-necked round-bottomed flask containing 6-methylcarvone (**2** and **3**) (530 mg, 3.22 mmol, 1 eq.), sodium bicarbonate (4.87 g, 59.96 mmol, 18 eq.) and phase transfer catalyst (adogen, 175 mg, 0.97 mmol, 0.3 eq.) in toluene-water solvent (40:40 mL). The mixture was gently heated and refluxed for 1.5 h under nitrogen atmosphere. Then, the reaction was cooled, the organic layer was separated, and the aqueous phase was washed with diethyl ether (2 x 25 mL). The combined organic layer was washed with water (1 x 25 mL), dried over ($MgSO_4$) and the solvent was removed *in vacuo*. The yield was purified with flash column chromatography (hexane:Et_2O 20:1), and further separation was with normal phase HPLC (hexane:Et_2O 97:3) to furnish four diastereomers of product with combined yield% (76%) and ratio (48%, 12%, 36%, 4% **11**:**12**:**13**:**14**) as colorless oil.

(2*S*,3*R*,6*R*)-Diastereomer (11): ^{1}H NMR (600 MHz, $CDCl_3$) δ 4.77 (s, 1H, C=C$\underline{H}_2$, H-9), 4.73 (s, 1H, C=C$\underline{H}_2$, H-9), 2.42 (dq, *J* = 12.7, 6.3 Hz, 1H, CH$_2$CH$_2$C$\underline{H}$, H-6), 2.36 (dq, *J* = 12.2, 6.0 Hz, 1H, CHC$\underline{H}$CH$_2$, H-2), 2.10–2.04 (m, 2H, C$\underline{H}_2$CH$_2$CH and CHC$\underline{H}$ CH$_2$, H-5 and H-3), 1.85 (qd, *J* = 12.9, 3.6 Hz, 1H, CHC$\underline{H}_2$CH$_2$, H-5), 1.76–1.72 (m, 1H, CHC$\underline{H}_2$CH$_2$, H-4), 1.70 (s, 3H, C$\underline{H}_3$C=CH$_2$), 1.37 (qd, *J* = 13.2, 3.6 Hz, 1H, CHC$\underline{H}_2$CH$_2$, H-4), 1.03 (d, *J* = 6.4 Hz, 3H, C$\underline{H}_3$CHCH$_2$), 0.91 (d, *J* = 6.4 Hz, 3H, C$\underline{H}_3$CHC=O).

^{13}C NMR (100 MHz; $CDCl_3$) δ 214.1 (C=O), 146.3 ($\underline{C}$=CH$_2$, C-8), 112.3 (C=$\underline{C}$H$_2$, C-9), 55.8 (CH$\underline{C}$HCH$_2$, C-3), 47.6 (CH$_2$CH$_2$$\underline{C}$H, C-6), 45.2 ($\underline{C}$HCHCH$_2$, C-2), 35.7 (CH$_2$$\underline{C}H_2$CH, C-5), 31.5 ($\underline{C}H_2CH_2$CH, C-4), 18.1 ($\underline{C}H_3$C=CH$_2$, C-10), 14.8($\underline{C}H_3$CHC=O, C-7), 12.1 ($\underline{C}H_3$CHCH$_2$, C-11).

HRMS (APCI) *m/z* for $C_{11}H_{18}O$, $[M]^+$, (Calc.: 166.1358, Found: 166.1354); oil; Anal. Calc. for $C_{11}H_{18}O$, Expected C, 79.5; H, 10.9; Found C, 79.4; H, 11.1; IR ν_{max} 2969, 2930, 2859, 1709 (C=O), 1645, 1450, 1375, 1177, 979, 865 cm^{-1}; $[\alpha]_D$ +27 ° (c 0.22, CH_2Cl_2, 24 °C).

(2*S*,3*R*,6*S*)-Diastereomer (12):

^{1}H NMR (600 MHz; $CDCl_3$) δ 4.78–4.76 (m, 1H, $C{=}C\underline{H}_2$, H-9), 4.72 (s, 1H, $C{=}C\underline{H}_2$, H-9), 2.60–2.52 (m, 2H, $C\underline{H}CH_2CH_2$ and $CH_2CHC\underline{H}$, H-2 and H-6), 2.16–2.10 (m, 1H, $CH_2CH_2C\underline{H}$, H-3), 1.92–1.84 (m, 2H, $CHCH_2C\underline{H}_2$ and $CHC\underline{H}_2CH_2$, H-4 and H-5), 1.73–1.70 (m, 1H, $CHC\underline{H}_2CH_2$, H-5), 1.71 (s, 3H, $C\underline{H}_3C{=}CH_2$), 1.64 (td, *J* = 8.4, 4.0 Hz, 1H, $CHCH_2C\underline{H}_2$, H-4), 1.17 (d, *J* = 7.2 Hz, 3H, $C\underline{H}_3CHCH$), 0.96 (d, *J* = 6.7 Hz, 3H, $C\underline{H}_3CHC{=}O$).

^{13}C NMR (100 MHz; $CDCl_3$) δ 216.9 (C=O), 146.4 ($\underline{C}{=}CH_2$, C-8), 112.3 ($C{=}\underline{C}H_2$, C-9), 53.6 ($CH\underline{C}HCH_2$, C-3), 43.9 ($CH_2CH_2\underline{C}H$, C-6), 43.6 ($\underline{C}HCHCH_2$, C-2), 31.6 ($CH\ \underline{C}H_2CH_2$, C-5), 25.6 ($CHCH_2\underline{C}H_2$, C-4), 18.9 ($\underline{C}H_3C{=}CH_2$, C-10), 16.9 ($\underline{C}H_3CHC{=}O$, C-7), 13.2 ($\underline{C}H_3CHCH$, C-11).

MS (ES) *m/z* 167 (MH^+, 70%), 189 (MNa^+, 60%); oil; Anal. Calc. for $C_{11}H_{18}O$, Expected C, 79.5; H, 10.9; Found C, 79.5; H, 10.9; IR ν_{max} 2963, 2929, 2858, 1708 (C=O), 1644, 1456, 1376, 1133, 896 cm^{-1}; $[\alpha]_D$ +18 ° (c 0.15, CH_2Cl_2, 24 °C).

(2*R*,3*R*,6*R*)-Diastereomer (13): ^{1}H NMR (600 MHz; $CDCl_3$) δ 4.89–4.88 (m, 1H, $C{=}C\underline{H}_2$, H-9), 4.73 (s, 1H, $C{=}C\underline{H}_2$, H-9), 2.72-2.68 (m, 1H, $CHC\underline{H}CH_2$, H-2), 2.59 (dq, *J* = 12.8, 6.4 Hz, 1H, $CH_2CH_2C\underline{H}$, H-6), 2.36 (dt, *J* = 12.4, 4.0 Hz, 1H, $CHC\underline{H}CH_2$, H-3), 2.06 (ddt, *J* = 13.2, 6.0, 3.4 Hz, 1H, $CHCH_2C\underline{H}_2$, H-5), 1.90 (qd, *J* = 13.0, 3.7 Hz, 1H, $CHCH_2C\underline{H}_2$, H-5), 1.70–1.68 (m, 1H, $CHC\underline{H}_2CH_2$, H-4), 1.67 (s, 3H, $C\underline{H}_3C{=}CH_2$), 1.29 (qd, *J* = 13.0, 3.7 Hz, 1H, $CHC\underline{H}_2CH_2$, H-4), 1.01 (d, *J* = 6.4 Hz, 3H, $C\underline{H}_3CHCH$), 0.94 (d, *J* = 7.4 Hz, 3H, $C\underline{H}_3CHC{=}O$).

^{13}C NMR (100 MHz; $CDCl_3$) δ 216.8 (C=O), 145.2 ($\underline{C}{=}CH_2$, C-8), 111.3 ($C{=}\underline{C}H_2$, C-9), 48.4 ($CH\underline{C}HCH_2$, C-3), 46.8 ($\underline{C}HCHCH_2$, C-2), 40.0 ($CH_2CH_2\underline{C}H$, C-6), 34.4 ($CH\underline{C}H_2CH_2$, C-4), 23.8 ($CHCH_2\underline{C}H_2$, C-5), 22.1 ($\underline{C}H_3C{=}CH_2$, C-10), 14.6 ($\underline{C}H_3CHC{=}O$, C-7), 11.9 (CH_3, CH_3CHCH, C-11).

HRMS (APCI) *m/z* for $C_{11}H_{18}O$, $[M]^+$, (Calc.: 166.1358, Found: 166.1353); Anal. Calc. for $C_{11}H_{18}O$, Expected C, 79.5; H, 10.9; Found C, 78.8; H, 11.2; IR ν_{max} 2969, 2931, 2869,1706 (C=O), 1645, 1451, 1375, 1129, 888 cm^{-1}; $[\alpha]_D$ **−**46 ° (c 0.26, CH_2Cl_2, 24 °C).

(2*R*,3*R*,6*S*)-Diastereomer (14): ^{1}H NMR (600 MHz; $CDCl_3$) δ 4.88 (quintet, *J* = 1.3 Hz 1H, C=C<u>H</u>$_2$, H-9), 4.38 (dq, *J* = 1.3, 0.9 Hz 1H, C=C<u>H</u>$_2$, H-9), 2.78–2.75 (m 1H, CH_2CH_2C<u>H</u>, H-6), 2.61 (qdd, *J* = 6.8, 5.6, 1.8 Hz, 1H, C<u>H</u>$CHCH_2$, H-2), 2.36 (dquintet, *J* = 12.5, 6.3, 1.2 Hz, 1H, CHC<u>H</u>CH_2, H-3), 2.01–1.93 (m, 1H, $CHCH_2C$<u>H</u>$_2$, H-5), 1.92–1.86 (m, 1H, CHC<u>H</u>$_2CH_2$, H-4), 1.83 (dq, *J* = 13.6, 3.4 Hz, 1H, $CHCH_2C$<u>H</u>$_2$, H-5), 1.70–1.60 (m, 4H, C<u>H</u>$_3C=CH_2$ and CHC<u>H</u>$_2CH_2$, H-4), 1.06 (d, *J* = 7.0 Hz, 3H, C<u>H</u>$_3$CHCH), 1.03 (d, *J* = 6.6 Hz, 3H, C<u>H</u>$_3$CHC=O).
^{13}C NMR (100 MHz; $CDCl_3$) δ 215.2 (C=O), 143.9 (<u>C</u>=CH_2, C-8), 113.1 (C=<u>C</u>H_2, C-9), 50.0 (CH_2CH_2<u>C</u>H, C-6), 47.4 (<u>C</u>H$CHCH_2$, C-2), 44.9 (CH<u>C</u>HCH_2, C-3), 31.1 ($CHCH_2$<u>C</u>H_2, C-5), 28.7 (CH<u>C</u>H_2CH_2, C-4), 24.2 (<u>C</u>$H_3C=CH_2$, C-10), 15.0 (<u>C</u>H_3CHC=O, C-7), 12.4 (<u>C</u>H_3CHCH, C-11). HRMS (APCI) *m/z* for $C_{11}H_{18}O$, $[M]^+$, (Calc.: 166.1358, Found: 166.1355); oil; Anal. Calc. for $C_{11}H_{18}O$, Expected C, 79.5; H, 10.9; Found C, 79.0; H, 11.0; IR ν_{max} 2970, 2934, 2873, 1708 (C=O), 1645, 1453, 1375, 1132, 892 cm^{-1}; $[\alpha]_D$ +14 ° (c 0.10, CH_2Cl_2, 24 °C).

Scale up bioreduction of (5*R*,6*R*)-6-methylcarvone 3 via PETNR

The enzyme (1mL of 150 μM stock, diluted to 2 μM in reaction), substrate (63 mg dissolved in 1.5 mL EtOH), $NADP^+$ (15 μM), G-6-PDH (750 U stock diluted to 10U in reaction), glucose (216 mg, final dilution reaction employed 15 mM) were added to 75 mL buffer solution of KP (pH 7.0, 50 mM), and the mixture incubated for 2.5 h at 30 °C at 135 rpm. TLC plate showed no starting material was detected, and the organic layer extracted and dried to afford pure isomer of (2*R*,3*R*,6*R*)-2-methyldihydrocarvone **13** (53 mg, 84%). The product was characterised with ^{1}H NMR spectroscopy, and showed identical spectrum to **13.** It was run on GC using DB-Wax column to yield one peak at retention times 20.97 min.

(2*S*,3*S*,6*R*)-2-Hydroxy-6-methyl-3-(prop-1-en-2-yl)cyclohexan-1-one, 15, and (2*S*,3*S*,6*S*)-2-Hydroxy-6-methyl-3-(prop-1-en-2-yl)cyclohexan-1-one, 16 (mixture of isomers)

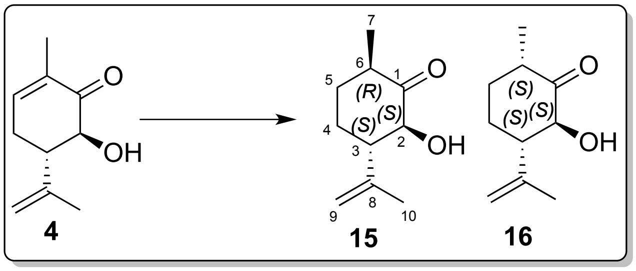

To a two-necked round bottomed flask containing *anti* 6-hydroxycarvone **4** (300 mg, 1.8 mmol, 1 eq.), sodium bicarbonate (42.63 g, 32.4 mmol, 18 eq.) and phase transfer catalyst (adogen, 94.5 mg, 0.51 mmol, 0.3 eq.) in toluene-water solvent system (20:20 mL), sodium dithionite (2.73 g, 15.6 mmol, 9 eq.) were added. The mixture was gently heated and reflexed in oil–bath for 1 h under nitrogen atmosphere. Then, the reaction was cooled, and the organic layer was separated, and the aqueous phase was washed with diethyl ether (1 x 20 mL). The combined organic layer was washed with water (1 x 10 mL), dried over ($MgSO_4$) and solvent removed *in vacuo*. The product was purified by flash column chromatography (hexane:EtOAc 7:1) to furnish (96:4) diastereomers (212 mg, 70%, with R_f 0.16 in hex: EtOAc 3:1) as a colourless oil.

(6*R*)-Diastereomer (15): ^{1}H NMR (400 MHz; $CDCl_3$) δ 4.89 (quint, *J* = 1.6, 1H, C=C<u>H</u>$_2$, H-9), 4.86–4.85 (m, 1H, C=C<u>H</u>$_2$, H-9), 4.09 (ddd, *J* = 11.4, 3.8, 1.6 Hz, 1H, C<u>H</u>OH, H-2), 3.67 (d, *J* = 3.9 Hz, 1H, OH), 2.50 (dqd, *J* = 12.8, 6.4, 1.6 Hz, 1H, CH_2CH_2C<u>H</u>, H-6), 2.25–2.18 (m, 1H, C<u>H</u>CH_2CH_2, H-3), 2.10 (ddt, *J* = 13.2, 6.3, 3.2 Hz, 1H, $CHCH_2$C<u>H</u>$_2$, H-5) 1.81 (dd, *J* = 1.5, 0.8 Hz, 3H, C<u>H</u>$_3$=C-CH_2), 1.79–1.78 (m, 1H, $CHCH_2$C<u>H</u>$_2$, H-5), 1.76 (d, *J* = 3.3 Hz, 1H, CHC<u>H</u>$_2CH_2$, H-4), 1.43–1.32 (m, 1H, CHC<u>H</u>$_2CH_2$, H-4), 1.11 (d, *J* = 6.5 Hz, 3H, C<u>H</u>$_3$CHC=O).

^{13}C NMR (100 MHz; $CDCl_3$) δ 212.3 (C=O), 145.1 (<u>C</u>=CH_2, C-8), 112.6 (C=<u>C</u>H$_2$, C-9), 76.8 (<u>C</u>HOH, C-2), 56.1 (CH_2CH_2<u>C</u>H, C-6), 43.2 (<u>C</u>HCH_2CH_2, C-3), 34.9 (CH<u>C</u>H$_2CH_2$, C-4), 29.0 ($CHCH_2$<u>C</u>H$_2$, C-5), 19.3 (<u>C</u>H$_3$C=CH_2, C-10), 14.0 (<u>C</u>H$_3$CH, C-7).

(248 and 249 mixture): HRMS (APCI) *m/z* for $C_{10}H_{16}O_2$, $[M]^+$ (Calc.: 168.1145, Found: 168.1148); oil; IR ν_{max} 3473 (OH), 2969, 2932, 2854, 1708 (C=O), 1666, 1448, 1375, 1233, 1126, 890 cm^{-1}.

(2*R*,3*S*,6*R*)-2-Hydroxy-6-methyl-3-(prop-1-en-2-yl)cyclohexan-1-one, 17, and (2*R*,3*S*,6*S*)-2-Hydroxy-6-methyl-3-(prop-1-en-2-yl)cyclohexan-1-one, 18 (mixture of isomers)

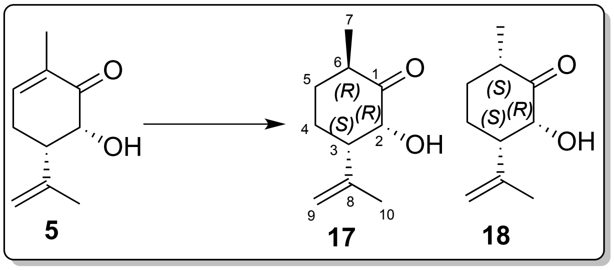

The title compounds were prepared from *syn* 6-hydroxycarvone **5** according to the procedure used for **15** and **16**. The product (170.0 mg, 56% with R_f 0.13 in hex; EtOAc 3:1, oil) was obtained as a colourless mixture of diastereomers (d.r. 95:5).

(6*R*)-Diastereomer (17): ^{1}H NMR (400 MHz; $CDCl_3$) δ 5.03–5.01 (m, 1H, C=C$\underline{H}_2$, H-9), 4.84–4.83 (m, 1H, C=C$\underline{H}_2$, H-9), 4.11 (d, J = 3.1 Hz, 1H, C$\underline{H}$OH, H-2), 3.02–2.93 (m, 1H, CHC$\underline{H}_2$CH_2, H-3), 2.46 (dt, J = 10.7, 3.3 Hz, 1H, CH_2CH_2C$\underline{H}$, H-6), 2.3 (bro s, IH, OH), 2.01–1.98 (m, 2H, CHCH_2C$\underline{H}_2$, and CHC$\underline{H}$CH_2, H-4 and H-5), 1.76 (dt, J = 1.3, 0.7 Hz, 3H, C$\underline{H}_3$=C-CH_2), 1.68–1.58 (m, 1H, CHCH_2C$\underline{H}_2$, H-5), 1.36–1.26 (m, 1H, CHC$\underline{H}_2$CH_2, H-4) 1.06 (d, J = 6.7 Hz, 3H, C$\underline{H}_3$CHC=O). ^{13}C NMR (100 MHz; $CDCl_3$) δ 213.7 (C=O), 143.9 ($\underline{C}$=CH_2, C-8), 113.3 (C=$\underline{C}H_2$, C-9), 75.1 ($\underline{C}$HOH, C-2), 51.2 (CH_2C$H_2$$\underline{C}$H, C-6), 40.3 (CH$\underline{C}HCH_2$, C-3), 33.6 (CH$\underline{C}H_2CH_2$, C-4), 23.1 (CHC$H_2$$\underline{C}H_2$, C-5), 22.7 ($\underline{C}H_3$=C-C$H_2$, C-10), 14.8 ($\underline{C}H_3$CHC=O, C-7).

(17 and 18 mixture): HRMS (APCI) *m/z* for $C_{10}H_{16}O_2$, $[M]^+$ (Calc.: 168.1145, Found: 168.1147); oil, IR ν_{max} 3452 (OH), 2968, 2932, 2855, 1709 (C=O), 1646, 1449, 1376, 1231, 1180, 855 cm^{-1}.

Scale up bioreduction of (5*S*,6*R*)-6-hydroxycarvone 5

An absolute EtOH solution of substrate **5** (93 mg in 2.5 mL; dilution to 5 mM in reaction) was added to buffer solution of KP (PH 7.0, 50 mM), followed by addition of of OYE2 (2 mL of 112 μM stock to 115 mL total, 2 μM), $NADP^+$ (15 μM), G-6-PDH (1200 U), glucose (320 mg total, 15 mM in final dilution) and the volume was completed to 115 mL with buffer solution. The reaction was incubated for 2.5 h at 30 °C, and extracted with ethyl acetate (3 x 50 mL). The organic layer was dried, concentrated, and purified via flash column chromatography using hex:EtOAc (5:1–3:1) as eluent to yield 10 mg (11%), d.e. >99%) of one diastereomer of (2*R*,3*S*,6*R*)-2-hydroxydihydrocarvone **17**. The product was confirmed using NMR, and IR spectroscopies, and run on GC using achiral DB-Wax column to give one peak at retention time 30.974 min.

(2*R*,3*R*,6*S*)-2-Hydroxy-6-methyl-3-(prop-1-en-2-yl)cyclohexan-1-one, 20, and (2*R*,3*R*,6*R*)-2-Hydroxy-6-methyl-3-(prop-1-en-2-yl)cyclohexan-1-one, 19 (mixture of isomers)

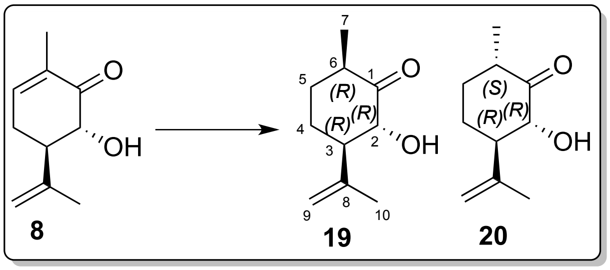

The title compounds were prepared from *anti* 6-hydroxycarvone **8** according to the procedure used for **15** and **16**. The yielded was (72 mg, 24% with R_f 0.16 in hex; EtOAc 3:1) of diastereomers mixture (10:1) as colourless oil.

(6*S*)-Diastereomer (20): ^{1}H NMR (400 MHz; $CDCl_3$) δ 4.91 (quint, *J* = 1.6 Hz, 1H, C=C<u>H</u>$_2$, H-9), 4.87–4.86 (m, 1H, C=C<u>H</u>$_2$, H-9), 4.10 (ddd, *J* = 11.3, 3.7, 1.5 Hz, 1H, C<u>H</u>OH, H-2), 3.68 (d, *J* = 3.8 Hz, 1H, OH), 2.50 (dquintetd, *J* = 12.8, 6.4, 1.6 Hz, 1H, CH_2CH_2C<u>H</u>, H-6), 2.26–2.19 (m, 1H, C<u>H</u>CH_2CH_2, H-3), 2.12 (ddt, *J* = 13.3, 6.2, 3.2 Hz, 1H, CHC<u>H</u>$_2CH_2$, H-4), 1.81 (dd, *J* = 1.4, 0.7 Hz, 3H, C<u>H</u>$_3$C=CH_2), 1.80–1.77 (m, 2H, CHC<u>H</u>$_2CH_2$, H-4 and CHCH_2C<u>H</u>$_2$, H-5) 1.44–1.33 (m, 1H, CHCH_2C<u>H</u>$_2$, H-5), 1.11 (d, *J* = 6.5 Hz, 3H, C<u>H</u>$_3$CHC=O).

^{13}C NMR (100 MHz; $CDCl_3$) δ 212.3 (C=O), 145.1 (<u>C</u>=CH_2, C-8), 112.6 (C=<u>C</u>H, C-3), 76.8 (<u>C</u>HOH, C-2), 56.1 (CH_2CH_2<u>C</u>H, C-6), 43.3 (<u>C</u>HCH_2CH_2, C-3), 35.0 (CH<u>C</u>H$_2CH_2$, C-4), 29.0 (CHCH_2<u>C</u>H$_2$, C-5), 19.4 (<u>C</u>H$_3$-C=CH_2, C-10), 14.1 (<u>C</u>H$_3$CHC=O, C-7).

(20 and 19 mixture): HRMS (APCI) *m/z* for $C_{10}H_{16}O_2$, $[M]^+$, (Calc.: 168.1145, Found: 168.1147); oil; IR ν_{max} 3477 (OH), 2969, 2933, 2861, 1708 (C=O), 1647, 1448, 1375, 1272, 1180, 841 cm^{-1}.

(2*S*,3*R*,5*R*)-2,3-Dimethyl-5-(prop-1-en-2-yl)cyclohexan-1-one, 22
(2*R*,3*R*,5*R*)-2,3-Dimethyl-5-(prop-1-en-2-yl)cyclohexan-1-one, 23
(2*R*,3*S*,5*R*)-2,3-Dimethyl-5-(prop-1-en-2-yl)cyclohexan-1-one, 24, and
(2*S*,3*S*,5*R*)-2,3-Dimethyl-5-(prop-1-en-2-yl)cyclohexan-1-one, 25 (mixture of isomers)[6]

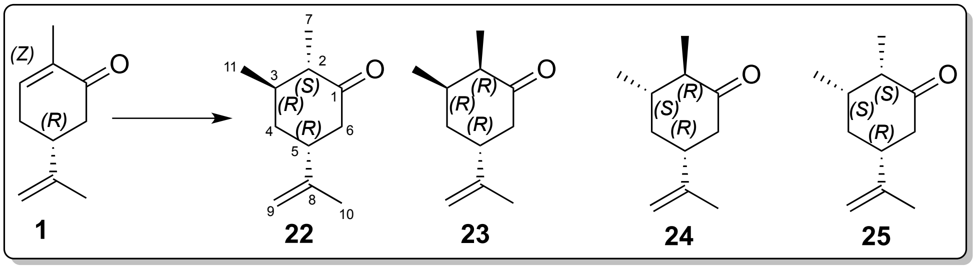

To magnetically stirred suspension of cupper bromide (CuBr, 5% mole, 36 mg) in dry THF (4 mL), TMA (5 mmol, 2M in hexane) was added under N_2 atmosphere, and the mixture allowed to stir for 5 min at RT. *R*-(−)-carvone **1** (5 mmol, 750 mg) was one-portion added, and the reaction mixture stirred for further 30 min. The mixture of reaction was carefully quenched with saturated NH_4Cl (1 mL), and left for 10 min to re-cooling. The solution was filtered with Duran-sintered disc filter, and the solid part washed with THF (3 x 10 mL). The combined organic layer was concentrated with reduced pressure evaporator, and the mixture was purified with flash column chromatography using hexane:EtOAc (10:1) to afford mixture of four diastereomers (1.5:1:1:0.1) as a colourless oil with yield (740 mg, 90%), and further separation was run on normal phase HPLC.

(Mixture of isomers): ^{1}H NMR (400 MHz, $CDCl_3$) δ 4.82–4.68 (m, 2H), 2.70–2.54 (m, 1H), 2.46–2.17 (m, 3H), 2.06–1.80 (m, 2H), 1.76–1.71 (m, 3H), 1.53–1.43 (m, 1H), major isomer (**22**): 1.11 (d, *J* = 6 Hz, 3H), 1.06 (d, *J* = 6.4 Hz, 3H); second isomer (**23**): 1.09 (d, *J* = 6.8 Hz, 3H), 1.03 (d, *J* = 6.4 Hz, 3H); third isomer (**24**): 0.98 (d, *J* = 6.8 Hz, 3H), 0.82 (d, *J* = 6.8 Hz, 3H).

^{13}C NMR (100 MHz; $CDCl_3$) δ 213.5, 213.0, 212.6 (C=O), 147.8, 147.7, 147.0 (C=CH_2, C-8), 111.8, 109.8, 109.7 (C=<u>C</u>H_2, C-9), 51.7 (CH), 51.3 (CH), 48.5 (CH), 46.7 (CH_2), 46.6 (CH_2), 45.2 (CH), 44.1 (CH_2), 41.2 (CH_2), 40.9 (CH), 40.1 (CH), 40.0 (CH_2), 37.8 (CH_2), 36.4 (CH), 35.2 (CH_2), 35.0 (CH), 22.0, 20.9, 20.7, 20.6, 20.5, 14.1, 13.2, 12.1, 11.6 (CH_3).

MS *m/z* 189 (MNa^+, 80%), 167 (MH^+, 60%); colourless oil; Anal. Calc. for $C_{11}H_{18}O$, Expected C, 79.5; H, 10.9; Found C, 79.3; H, 10.7;IR ν_{max} 2969, 2929, 2873, 1708 (C=O), 1645, 1451, 1375, 1213, 889 cm^{-1}.

General enzymatic procedure for Baeyer–Villiger oxidation using CHMOs

The standard enzymatic reaction (1 mL) was achieved in buffer solution of Tric.HCl (50 mM, pH 7.5), comprises [substrate] = 5 mM, [$NADP^+$] = 15 μM, GDH = 10 U, glucose or glucose-6-phosphate 15 mM, [Enzyme] = 2 or 10 μM. The reaction mixture was shaken at required temperature (25 or 30 or 37 °C) at 135 rpm for the desired time. The reaction was then terminated by extraction into an organic layer with 800 μL of EtOAc:internal standard (+)-limonene (99.5:0.5). The organic mixture was dried over ($MgSO_4$), and analysed with Varianchiralsil-DEX CB column.

Scale up of BV oxidation of (2*R*,3*R*,6*R*)-2,6-dimethyl-3-(prop-1-en-2-yl)cyclohexan-1-one 21 by CHMOs

(3*R*,6*S*,7*R*)-3,7-Dimethyl-4-(prop-1-en-2-yl)oxepan-2-one, 21

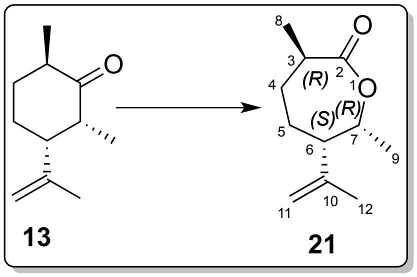

In conical flask contains buffer solution of Tris.HCl pH 7.5, 60 mL, a solution of **13** (50 mg dissolved in 1.2 mL absolute EtOH), $NADP^+$ (15 μM), GDH (600 U), glucose (15 mM) and CHMOs (120 μM stock diluted 1 mL to 60 mL, 2 μM employed) were rapidly mixed. The enzymatic reaction mixture was incubated for 24 h at 25 °C, and monitored via TLC plate. The organic layer was extracted with EtOAc (3 x 50 mL), dried over ($MgSO_4$), and concentrated to afford a lactone **21** with yield (49 mg, 90%) as a colorless oil.

(3*R*,6*S*,7*R*)-Lactone (21): ^1H NMR (400 MHz; $CDCl_3$) δ 4.88 (quint, *J* = 1.5 Hz, 1H, C=C<u>H</u>₂, H-11), 4.77–4.75 (m, 1H, C=C<u>H</u>₂, H-11), 4.66 (qd, *J* = 7.2, 3.3 Hz, 1H, CHC<u>H</u>OCO, H-7), 2.81 (dqd, *J* = 10.6, 6.8, 5.0 Hz, 1H, C<u>H</u>CH₂CH₂, H-3), 2.40 (ddd, *J* = 8.7, 7.1, 3.3 Hz, 1H, C<u>H</u>CHOCO, H-6), 1.92–1.81 (m, 3H, CHC<u>H</u>₂CH₂ and CHCH₂C<u>H</u>₂, 2 x H-4 and H-5), 1.77 (dd, *J* = 1.4, 0.8 Hz,

3H, C<u>H</u>$_3$C=CH$_2$, 3 x H-12), 1.47–1.39 (m, 1H, CHCH$_2$C<u>H</u>$_2$, H-5), 1.34 (d, J = 7.2 Hz, 3H, C<u>H</u>$_3$CHOCO, 3 x H-9), 1.28 (d, J = 6.8 Hz, 3H, C<u>H</u>$_3$CHCH$_2$, 3 x H-8).

^{13}C NMR (100 MHz; CDCl$_3$) δ 177.1 (C=O), 145.0 (<u>C</u>=CH$_2$, C-10), 113.6 (C=<u>C</u>H$_2$, C-11), 75.4 (CH$_2$CH<u>C</u>H, C-7), 50.7 (CH$_2$<u>C</u>HCH, C-6), 41.2 (<u>C</u>HCH$_2$CH$_2$, C-3), 30.2 (CH<u>C</u>H$_2$CH$_2$, C-4), 27.4 (CHCH$_2$<u>C</u>H$_2$, C-5), 22.2 (<u>C</u>H$_3$C=CH$_2$, C-12), 19.0 (<u>C</u>H$_3$CHCH$_2$, C-7), 16.1 (CH$_3$CH, C-9).

HRMS (ES) *m/z* for $C_{11}H_{19}O_2$, [MH]$^+$, (Calc.: 183.1385, Found: 183.1383); oil; IR ν_{max} 2972, 2934, 2874, 1717 (C=O ester), 1644, 1456, 1378, 1261, 1208, 1095 cm^{-1}; $[\alpha]_D$ +50 ° (c 0.04, CH$_2$Cl$_2$, 24 °C), Retention time 13.868 min.

Scale up of BV oxidation of two isomers mixture of 3-methyldihydrocarvone 22 and 23 by CHMOs

(3*R*,4*R*,6*S*)-3,4-Dimethyl-6-(prop-1-en-2-yl)oxepan-2-one, 26, and (3*S*,4*R*,6*S*)-3,4-Dimethyl-6-(prop-1-en-2-yl)oxepan-2-one, 27 (mixture of normal and abnormal lactones)

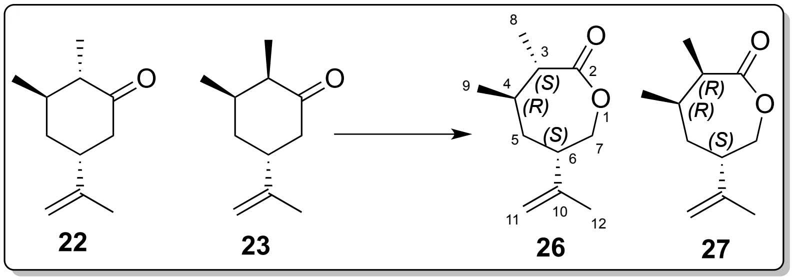

In conical flask contains buffer solution of Tris.HCl pH 7.5, 70 mL, a solution of **22** and **23** (63 mg dissolved in 1.5 mL absolute EtOH), NADP$^+$ (15 μM), GDH (750 U diluted to 10 U in reaction), glucose (15 mM) and CHMOs (150 μM stock diluted 1 mL to 75 mL, 2 μM employed) were rapidly mixed. The enzymatic reaction mixture was incubated for 24 h at 25 °C, and monitored via TLC plate. The organic layer was extracted with EtOAc (3 x 50 mL), dried over (MgSO$_4$), and concentrated to afford a mixture of abnormal lactones **26 and 27** as a colorless volatile oil (62 mg, 90%).

(3*R*,4*R*,6*S*)-lactone (26): ^{1}H NMR (400 MHz; CDCl$_3$) δ 4.82–4.79 (m, 2H, C=C<u>H</u>$_2$, 2 x H-11), 4.15–4.12 (m, 2H, C<u>H</u>$_2$-O, 2 x H-7), 2.59 (dq, J = 10.1, 6.8 Hz, 1H, C<u>H</u>=O, H-3), 2.35 (ddt, J = 12.0, 8.8, 3.1 Hz, 1H, C<u>H</u>CH$_2$-O, H-6), 1.88–1.84 (m 1H, C<u>H</u>$_2$CHCH, H-5), 1.75–1.73 (m, 3H, C<u>H</u>$_3$C=CH$_2$, 3 x H-12), 1.69–1.59 (m, 1H, C<u>H</u>CHC=O, H-4), 1.48–1.46 (m, 1H, CHCHC<u>H</u>$_2$, H-

5), 1.16 (d, *J* = 6.8 Hz, 3H, C<u>H</u>$_3$CHC=O, 3 x H-8), 1.00 (d, *J* = 6.8 Hz, 3H, C<u>H</u>$_3$CHCHC=O, 3 x H-9).

^{13}C NMR (100 MHz; CDCl$_3$) δ 178.0 (C=O), 145.9 (<u>C</u>=CH$_2$, C-10), 111.2 (C=<u>C</u>H$_2$, C-11), 71.9 (<u>C</u>H$_2$-O, C-7), 46.4 (<u>C</u>HCH$_2$-O, C-6), 44.3 (CHCH<u>C</u>H$_2$, C-5), 41.5 (CH<u>C</u>HC=O, C-3), 41.0 (<u>C</u>HCHC=O, C-4), 22.0 (<u>C</u>H$_3$C=CH$_2$, C-12), 16.7 (<u>C</u>H$_3$CHCH, C-8), 13.7 (<u>C</u>H$_3$CH, C-9).

(3*S*,4*R*,6*S*)-lactone (27): ^{1}H NMR (400 MHz; CDCl$_3$) δ 4.71–4.69 (m, 2H, C=C<u>H</u>$_2$, H-11), 4.19– 4.16 (m, 2H, C<u>H</u>$_2$-O, 2 x H-7), 3.02 (qd, *J* = 6.8, 1.5 Hz, 1H, CHC<u>H</u>C=O, H-3), 2.49 (dq, *J* = 10.3, 5.4 Hz, 1H, C<u>H</u>CH$_2$-O, H-6), 1.98–1.92 (m, 2H, C<u>H</u>$_2$CHCH, 2 x H-5), 1.76–1.74 (m, 3H, C<u>H</u>$_3$C=CH$_2$, 3 x H-12), 1.19 (d, *J* = 6.8 Hz, 3H, C<u>H</u>$_3$CHC=O, 3 x H-8), 0.94 (d, *J* = 7.2 Hz, 3H, C<u>H</u>$_3$CHCHC=O, 3 x H-9).

^{13}C NMR (100 MHz; CDCl$_3$) δ 176.4 (C=O), 145.7 (<u>C</u>=CH$_2$, C-10), 111.4 (C, C=<u>C</u>H$_2$, C-11), 71.2 (CH<u>C</u>H$_2$-O, C-7), 44.3 (<u>C</u>HCH$_2$-O, C-6), 41.6 (<u>C</u>HCHCH$_2$, C-3), 41.3 (<u>C</u>H$_2$CHCH, C-5), 34.1 (CH<u>C</u>HCH$_2$, C-4), 21.8 (<u>C</u>H$_3$C=CH$_2$, C-12), 20.6 (<u>C</u>H$_3$CHCH, C-8), 15.3 (<u>C</u>H$_3$CH, C-9).

Abnormal lactones mixture (26 and 27): HRMS (ES) *m/z* for $C_{11}H_{19}O_2$, $[MH]^+$, (Calc.: 183.1385, Found: 183.1384); oil; IR ν_{max} 2970, 2933, 2887, 1715 (C=O ester), 1640, 1458, 1391, 1278, 1234, 1111 cm^{-1}. Retention time 15.942 and 16.165 min of **26** and **27** respectively.

2 Spectral data

(5*R*,6*S*)-(+)-5-Isopropenyl-2,6-dimethylcyclohexane-2-enone, 2

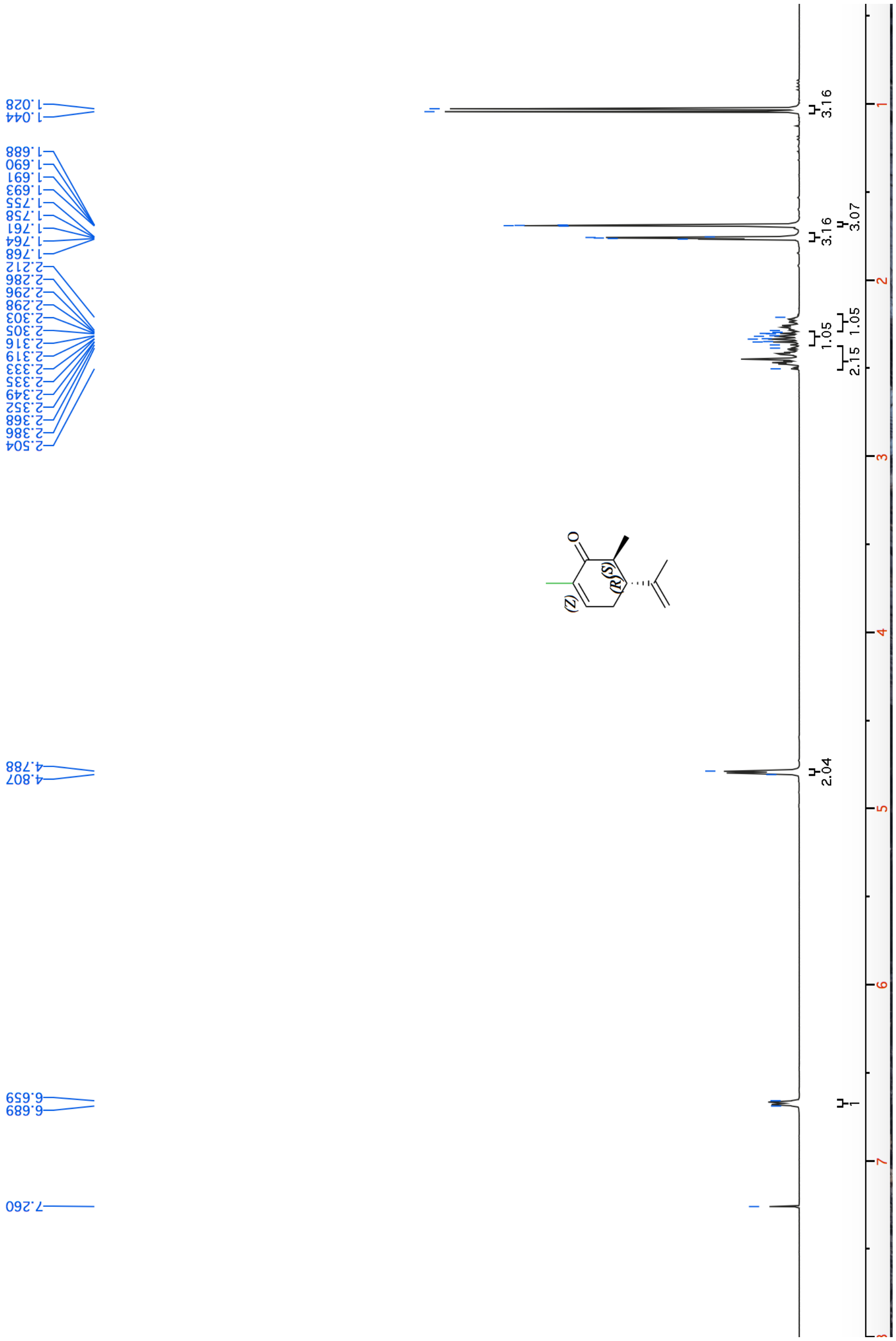

^{13}C (100 MHz): (5*R*,6*S*)-(+)-5-Isopropenyl-2,6-dimethylcyclohexane-2-enone, 2

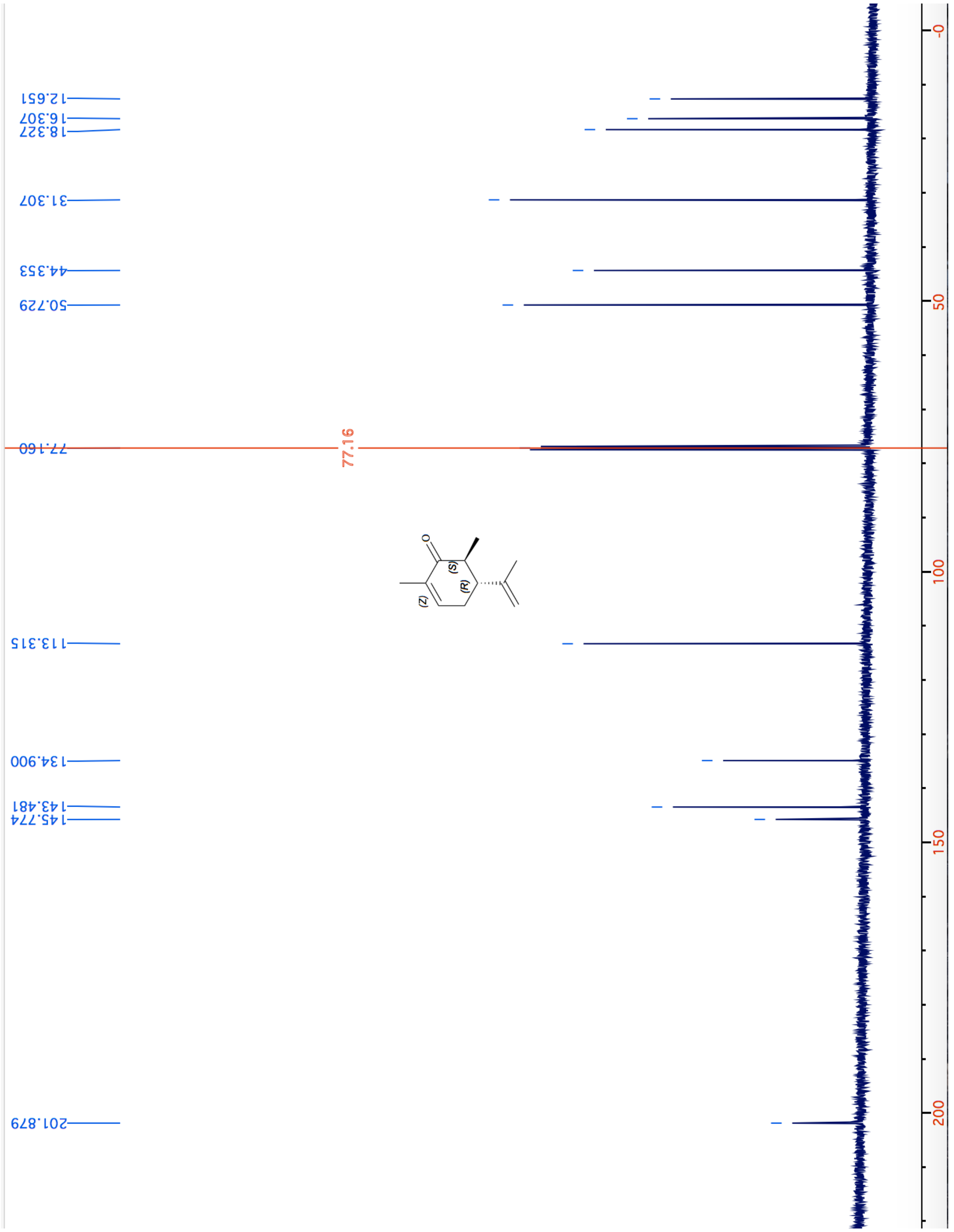

COSY: (5*R*,6*S*)-(+)-5-Isopropenyl-2,6-dimethylcyclohexane-2-enone, 2

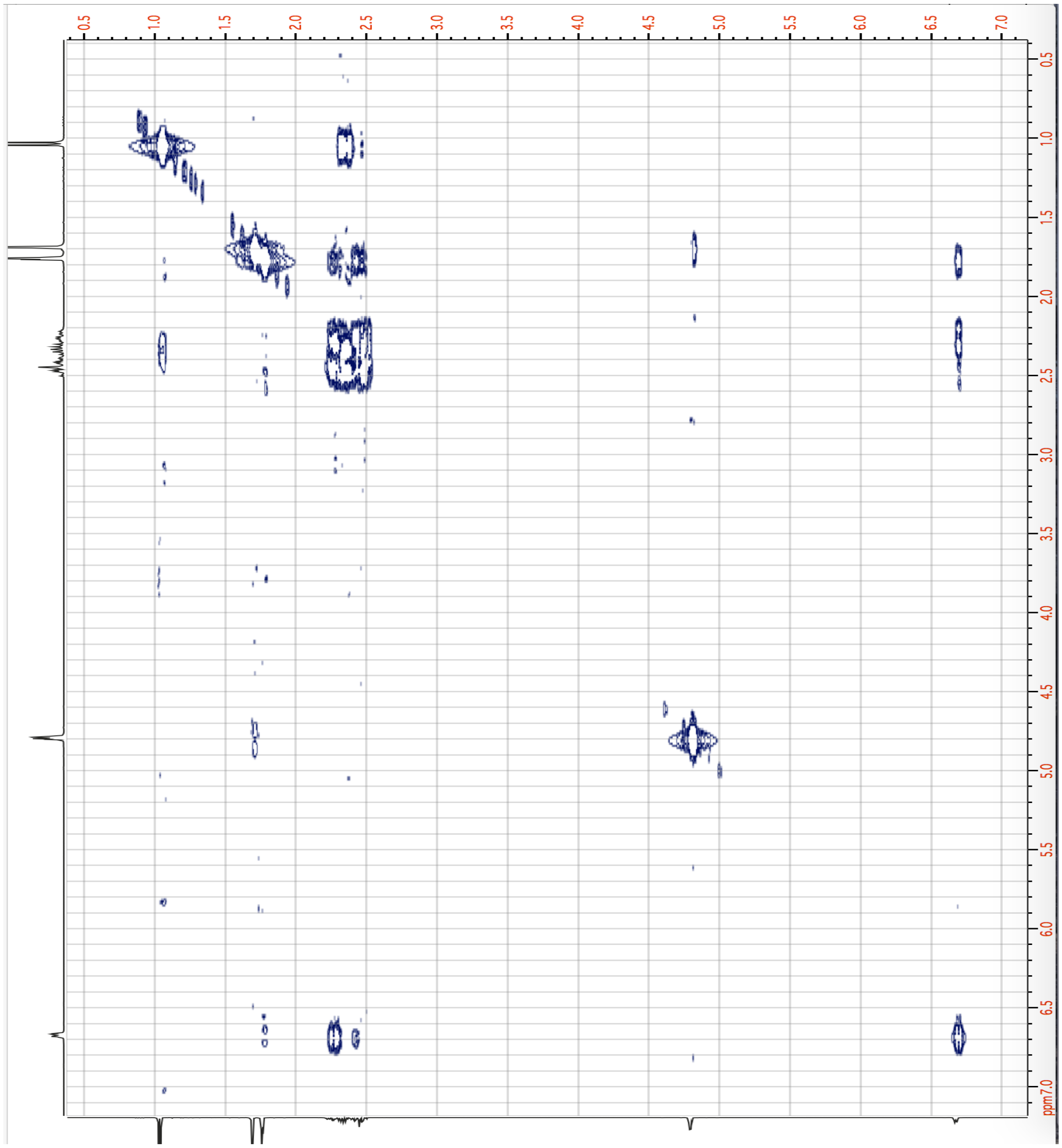

(5*R*,6*R*)-(+)-5-Isopropenyl-2, 6-dimethylcyclohexane-2-enone, 3

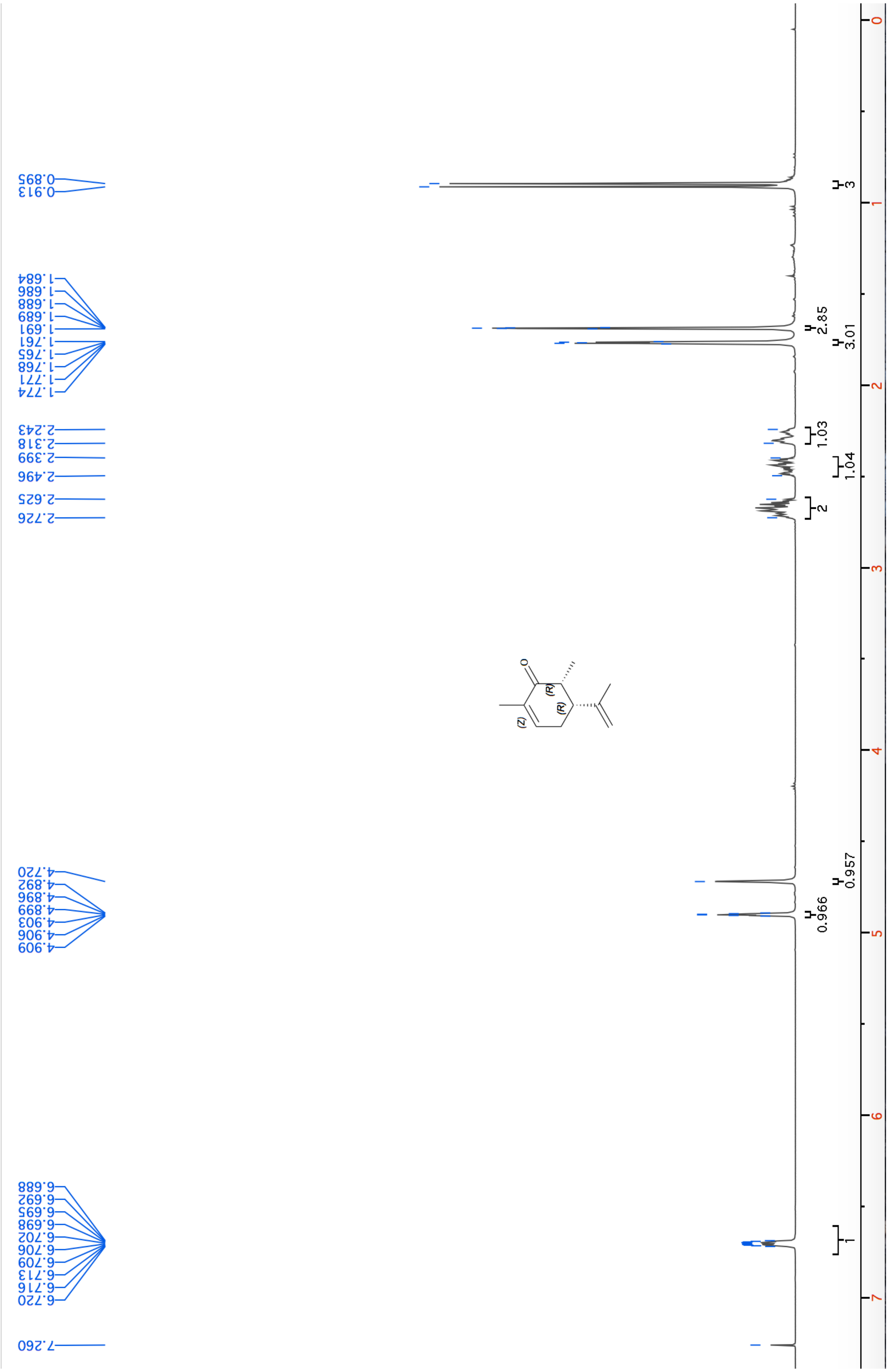

(5*R*,6*R*)-(+)-5-Isopropenyl-2, 6-dimethylcyclohexane-2-enone, 3

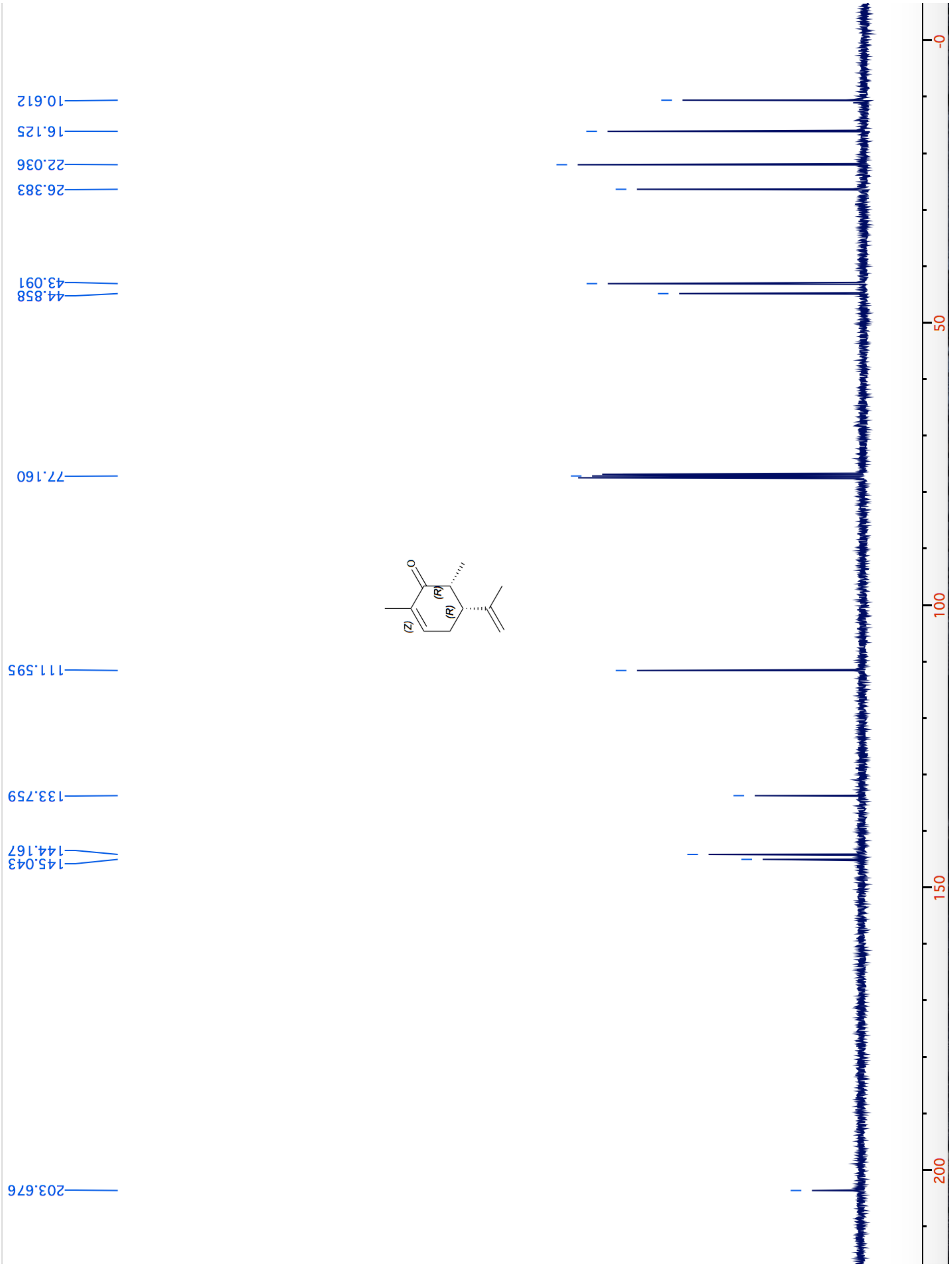

Supporting Information

(5*R*,6*R*)-(+)-5-Isopropenyl-2, 6-dimethylcyclohexane-2-enone, 3

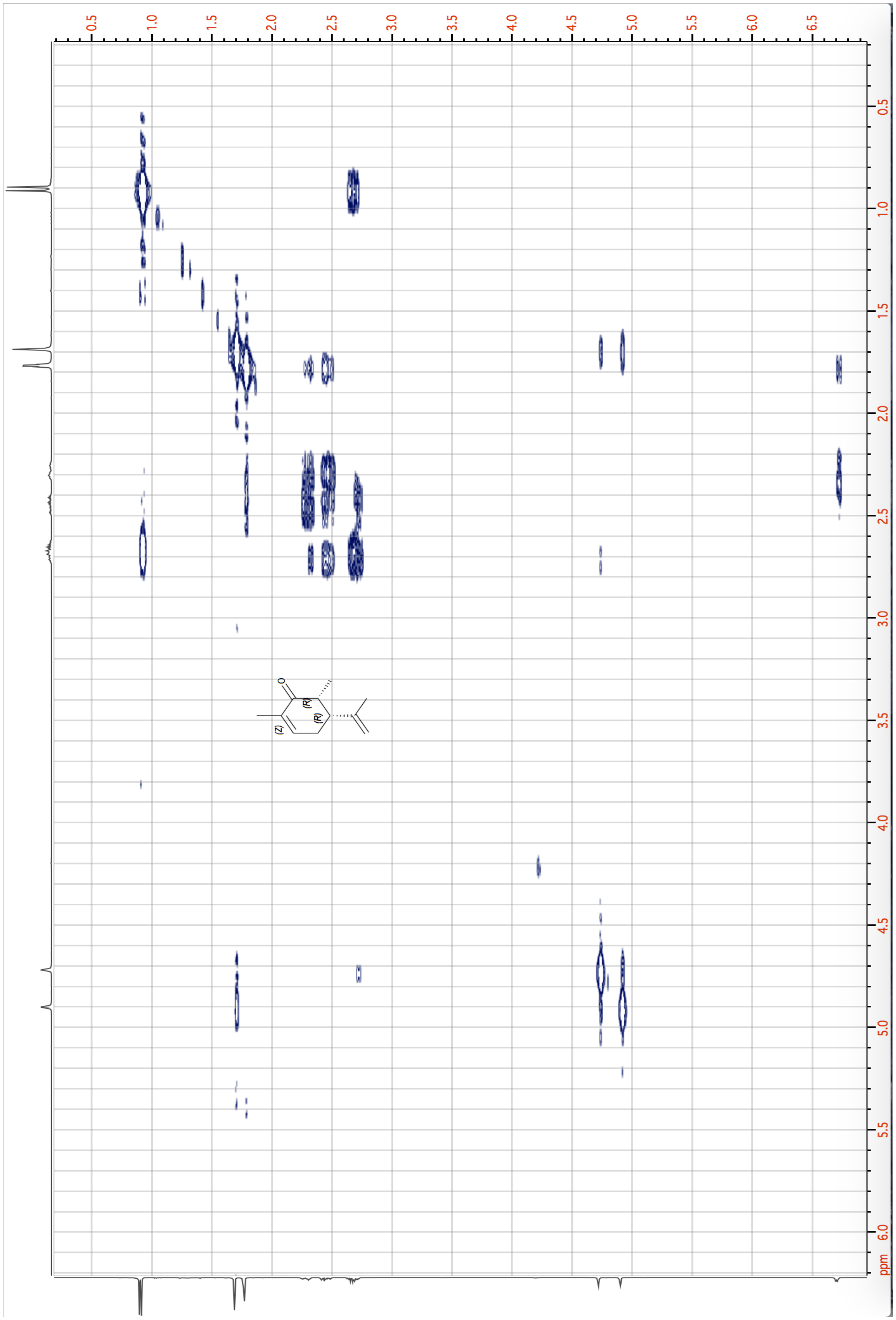

(5*S*,6*R*)-6-Hydroxy-2-methyl-5- (prop-1-en-2-yl) cyclohex-2-en-1-one, 4

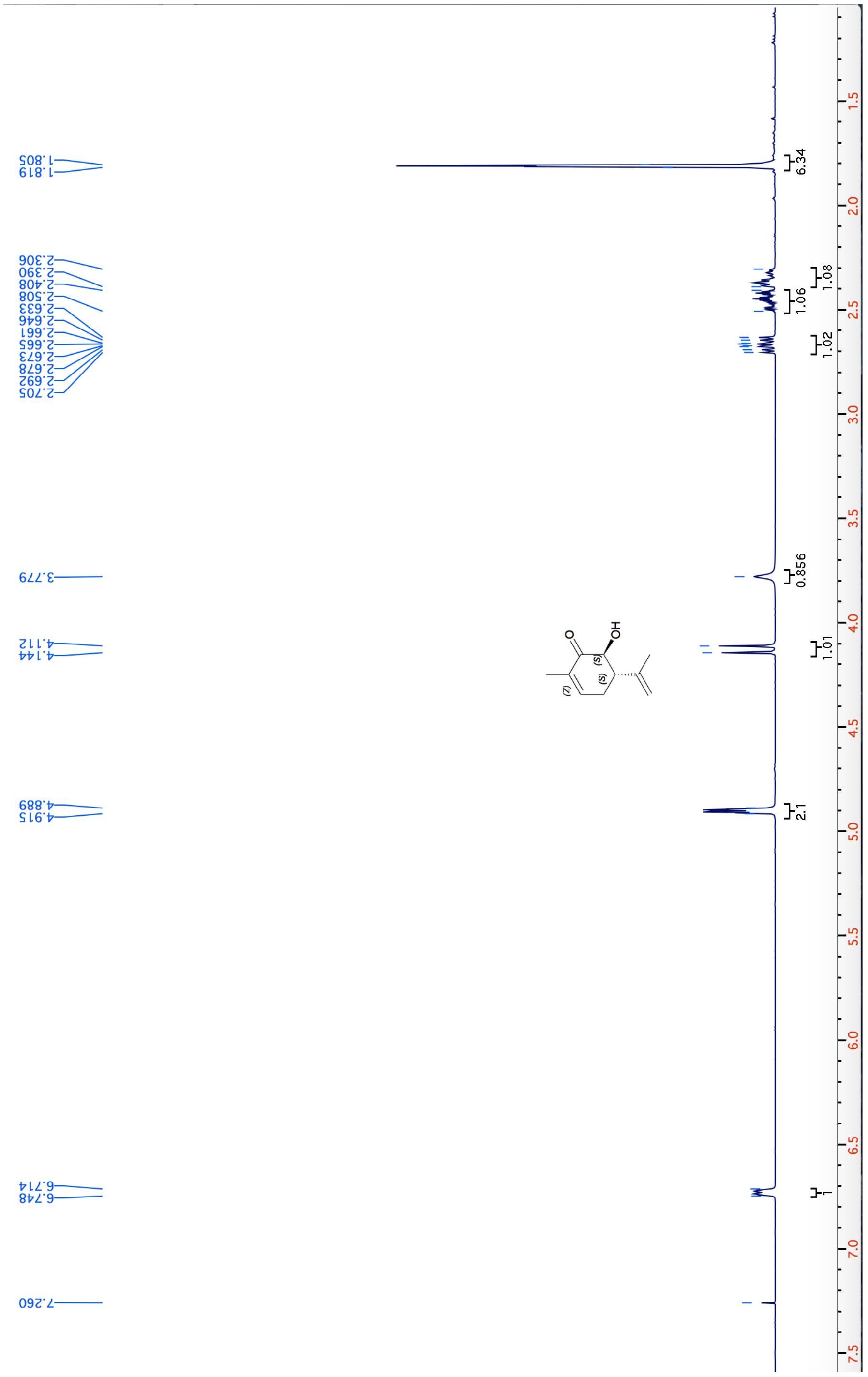

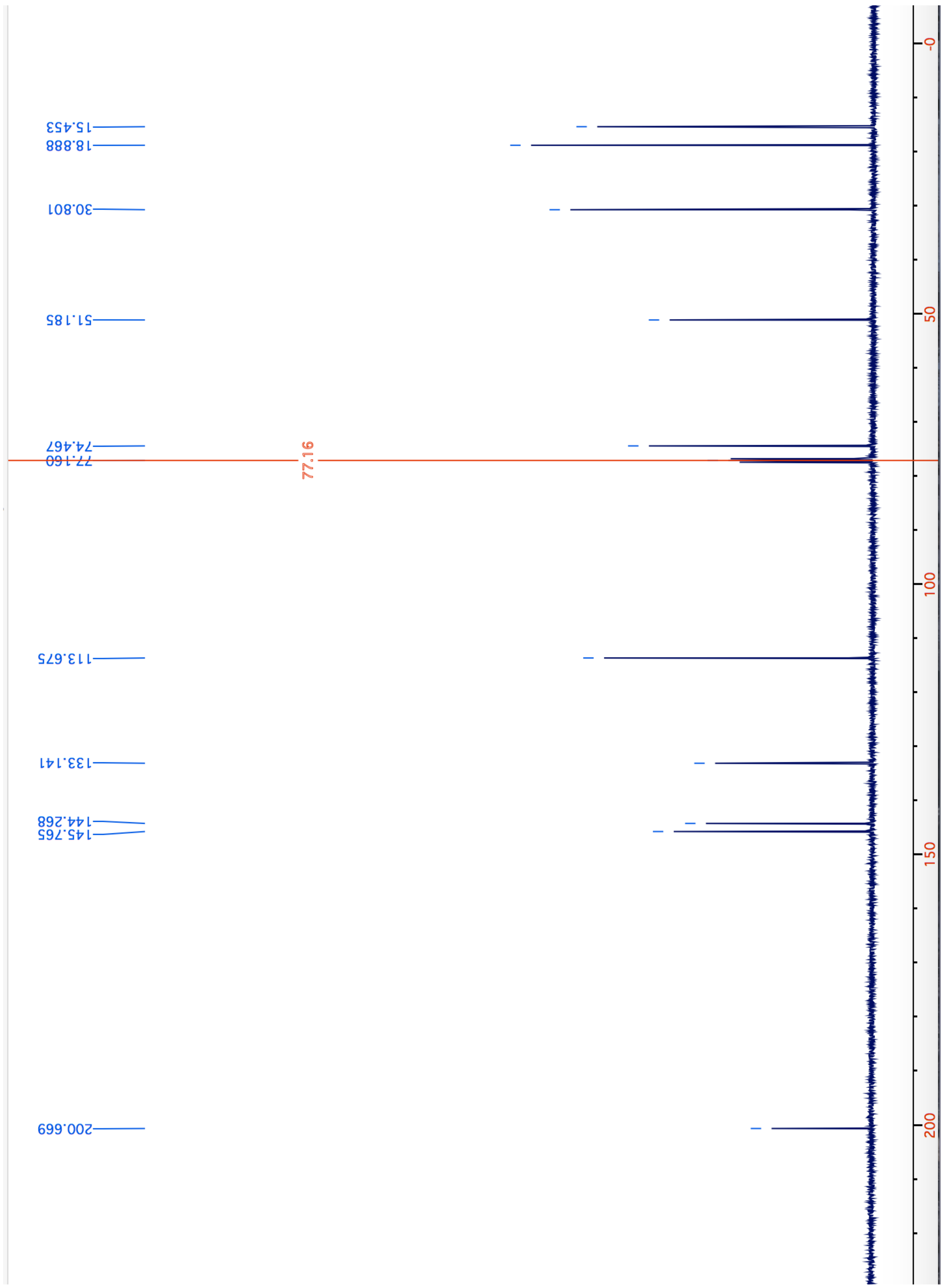
15.453
18.888
30.801
51.185
74.467
77.160
77.16
113.675
133.141
144.268
145.765
200.669
0
50
100
150
200

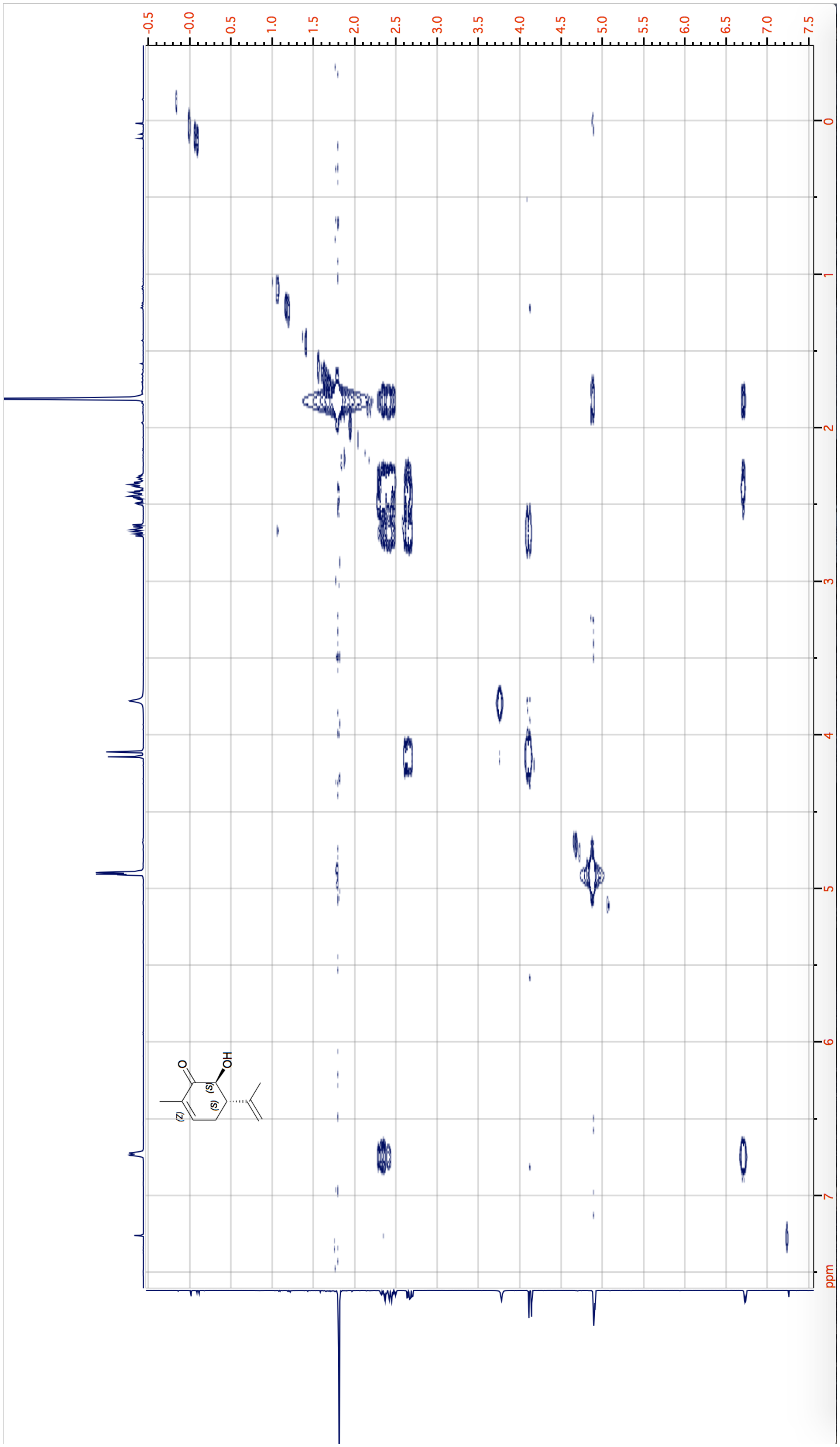

ppm
O
OH
(S)
(S)
(Z)

(5*S*,6*S*)-6-Hydroxy-2-methyl-5- (prop-1-en-2-yl) cyclohex-2-en-1-one, 5

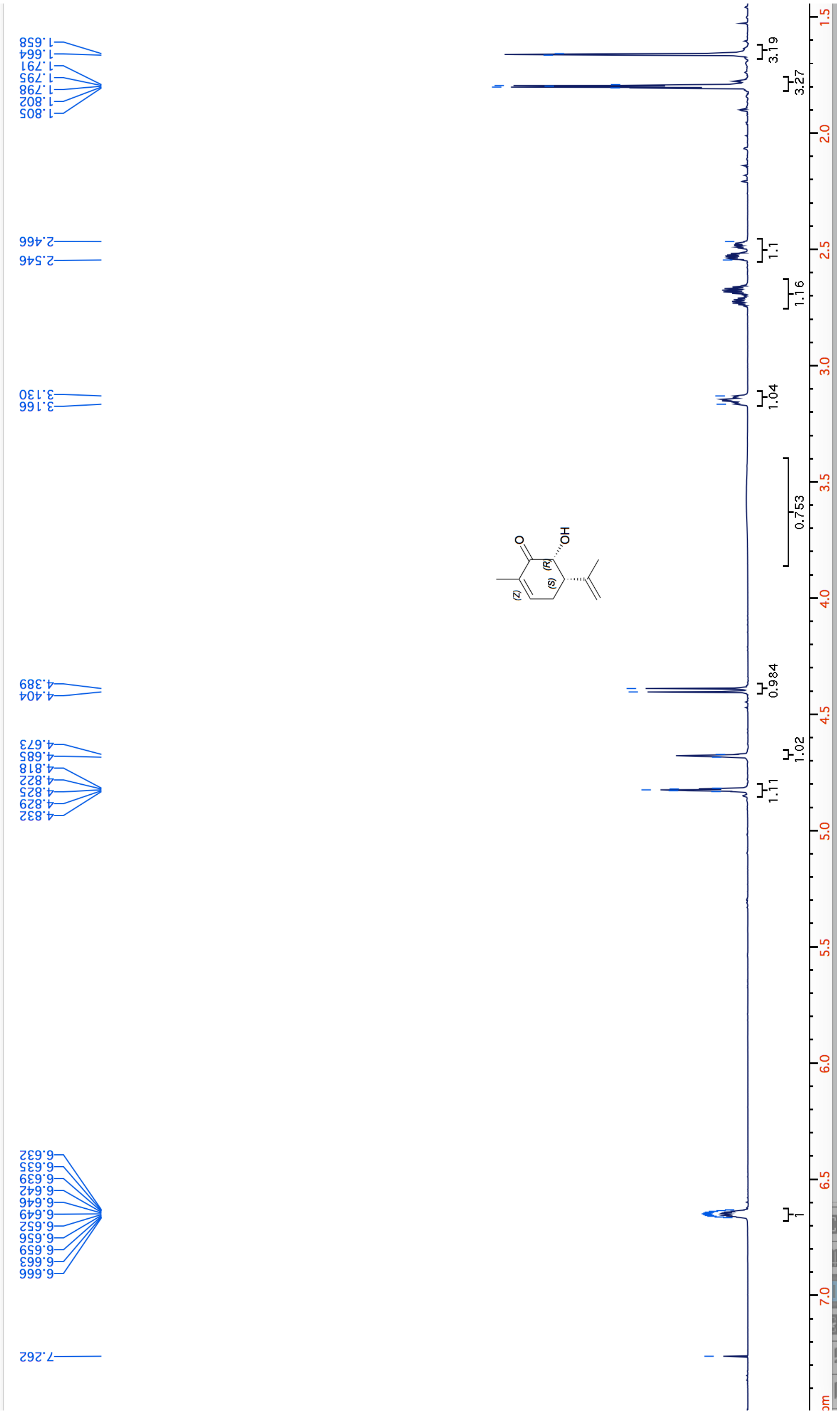

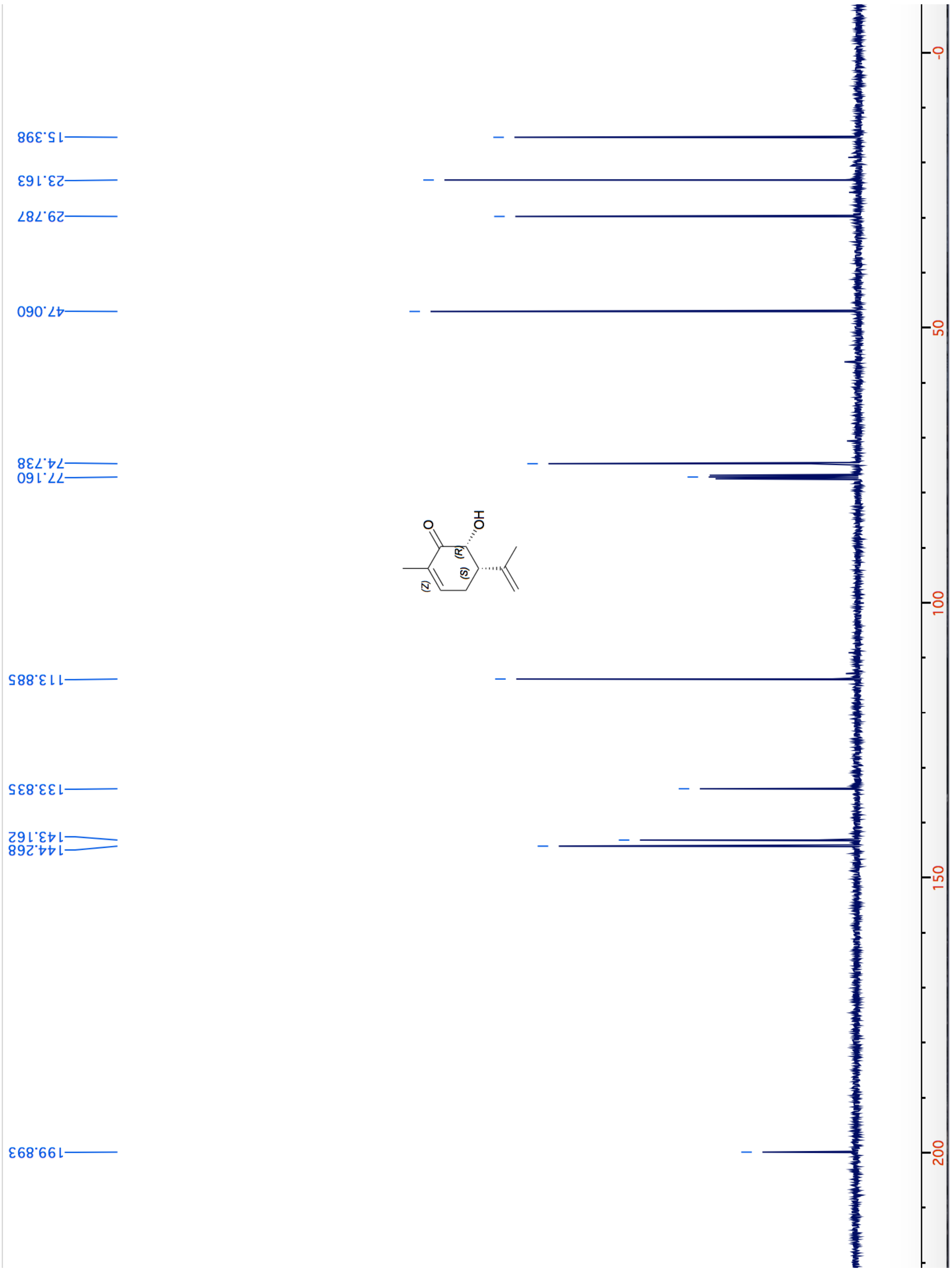
15.398
23.163
29.787
47.060
74.738
77.160
113.885
133.835
143.162
144.268
199.893
O
OH
(R)
(S)
(Z)
0
50
100
150
200

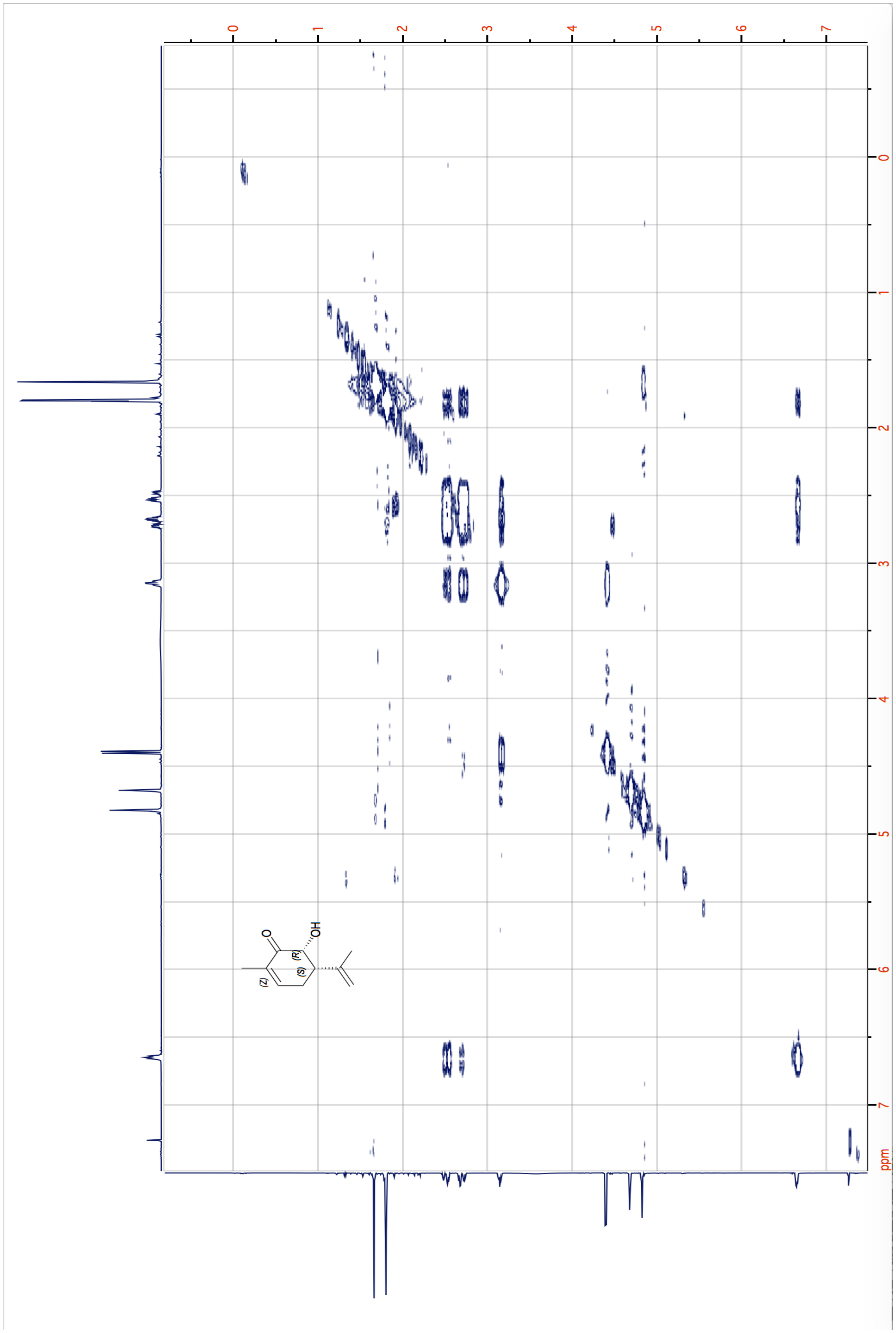

0
1
2
3
4
5
6
7
ppm
OH
O
(R)
(S)
(Z)

(2*S*,3*R*,6*R*)-2,6-Dimethyl-3- (prop-1-en-2-yl)cyclohexan-1-one, 11

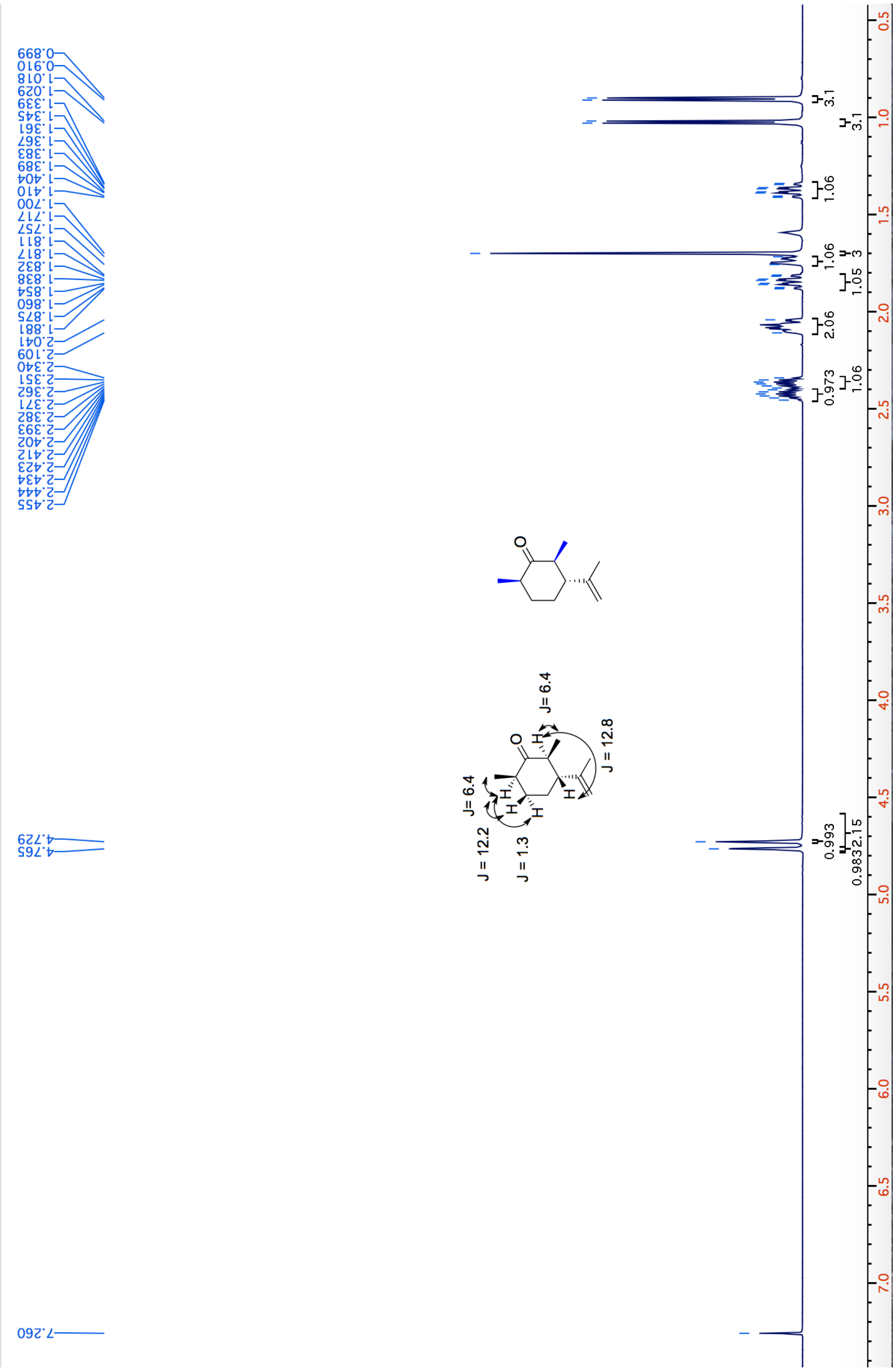

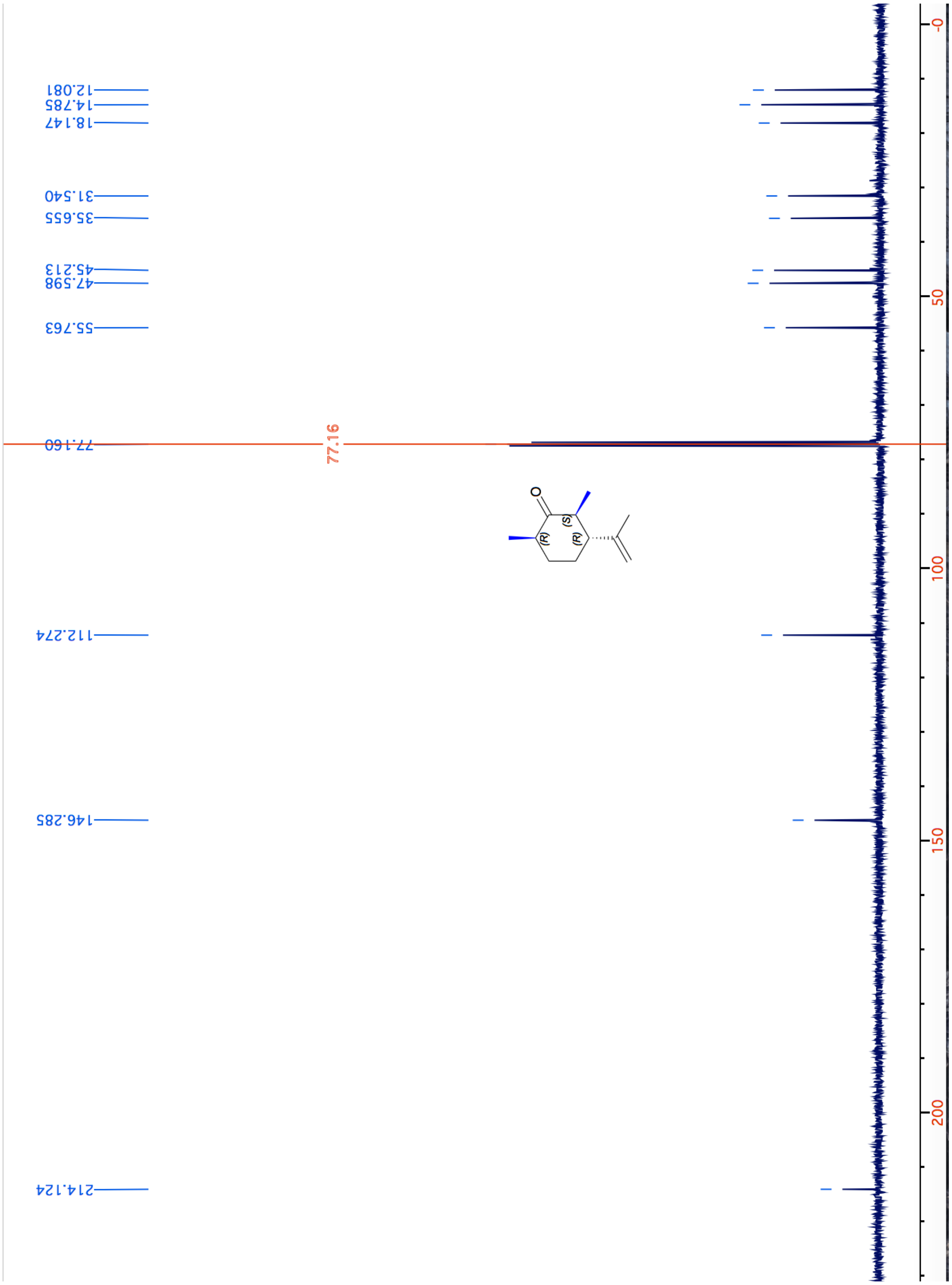
12.081
14.785
18.147
31.540
35.655
45.213
47.598
55.763
77.160
77.16
112.274
146.285
214.124
(R)
(S)
(R)
O
0
50
100
150
200

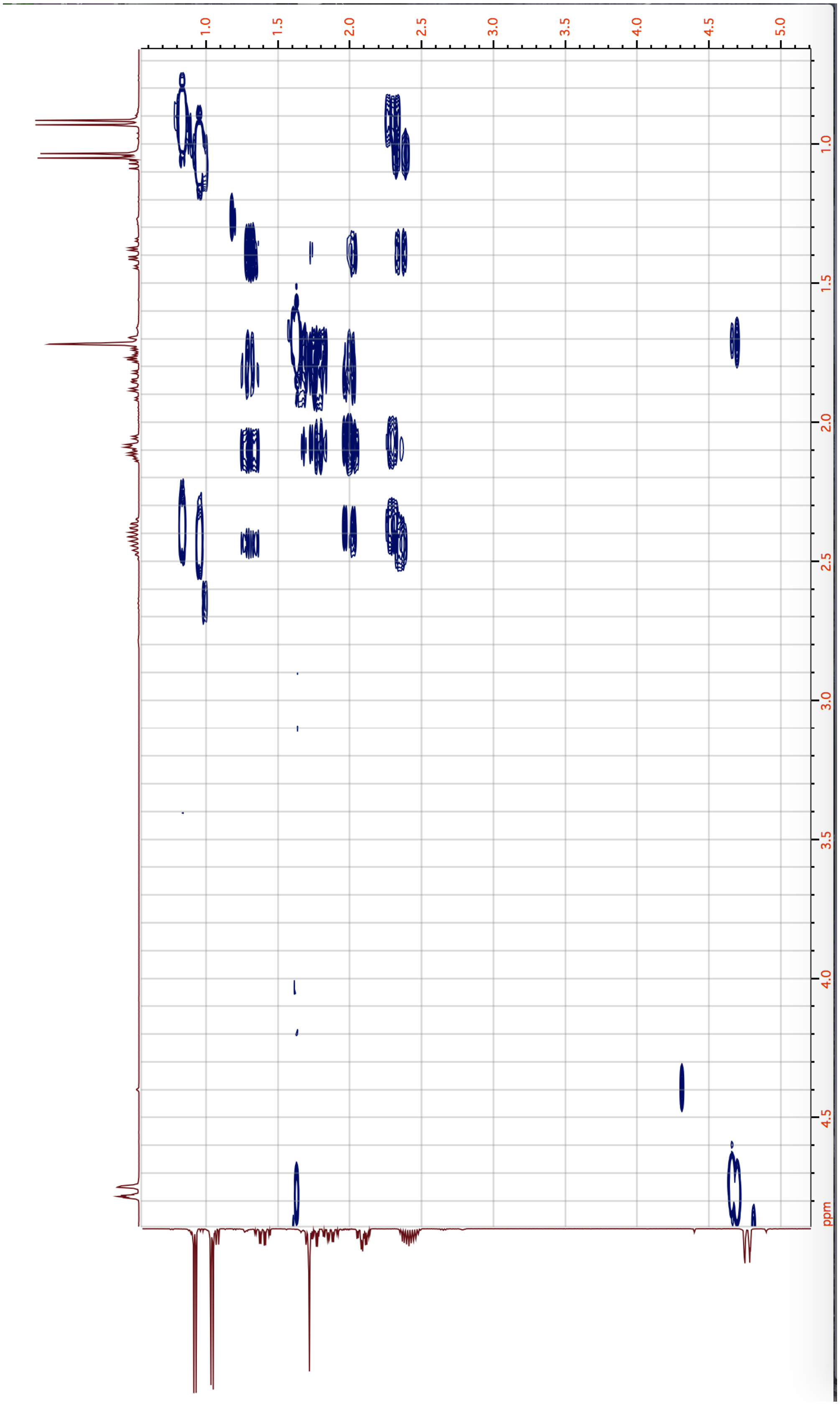
1.0
1.5
2.0
2.5
3.0
3.5
4.0
4.5
5.0
ppm

(2*S*,3*R*,6*S*)-2,6-Dimethyl-3-(prop-1-en-2-yl)cyclohexan-1-one, 12

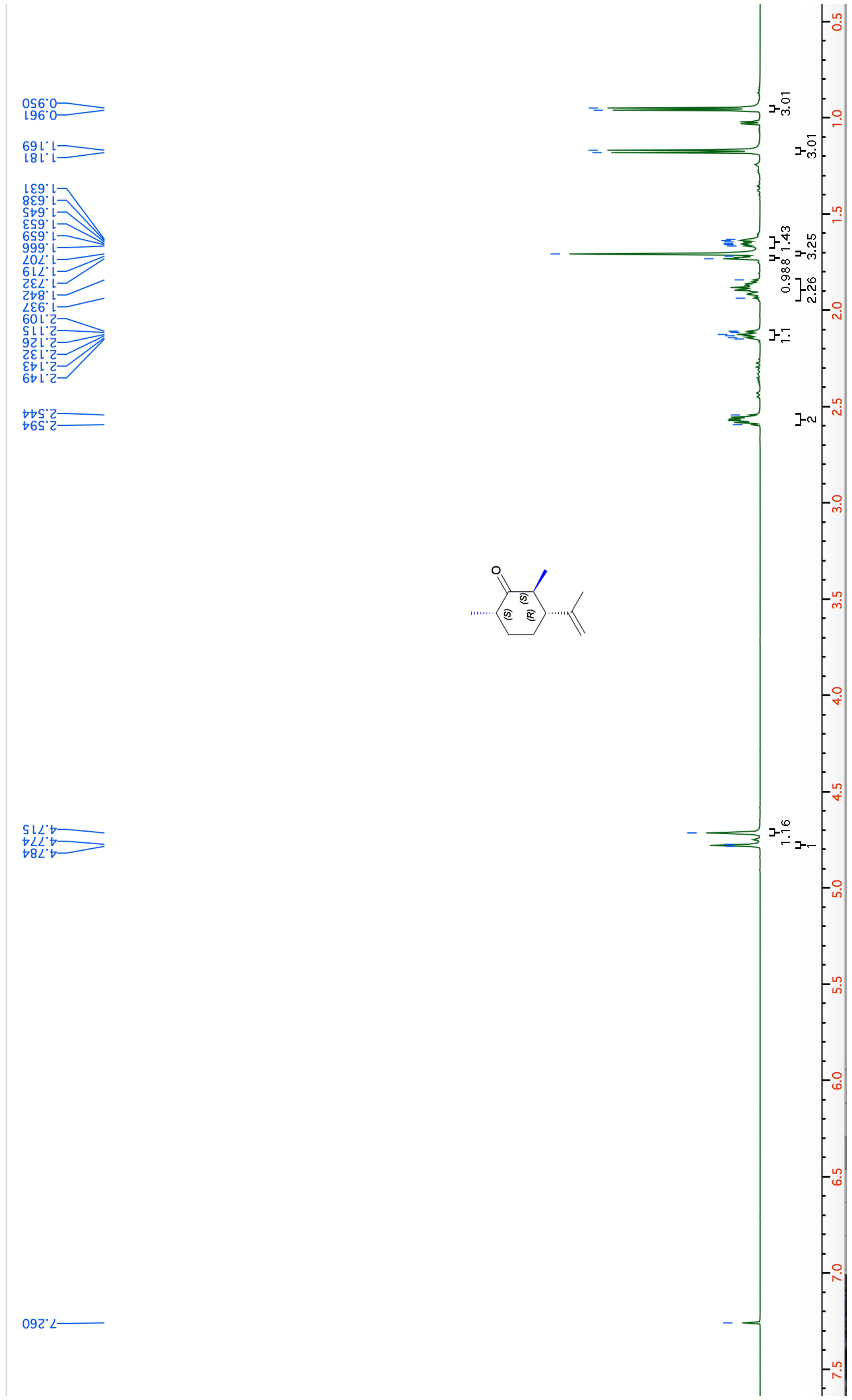

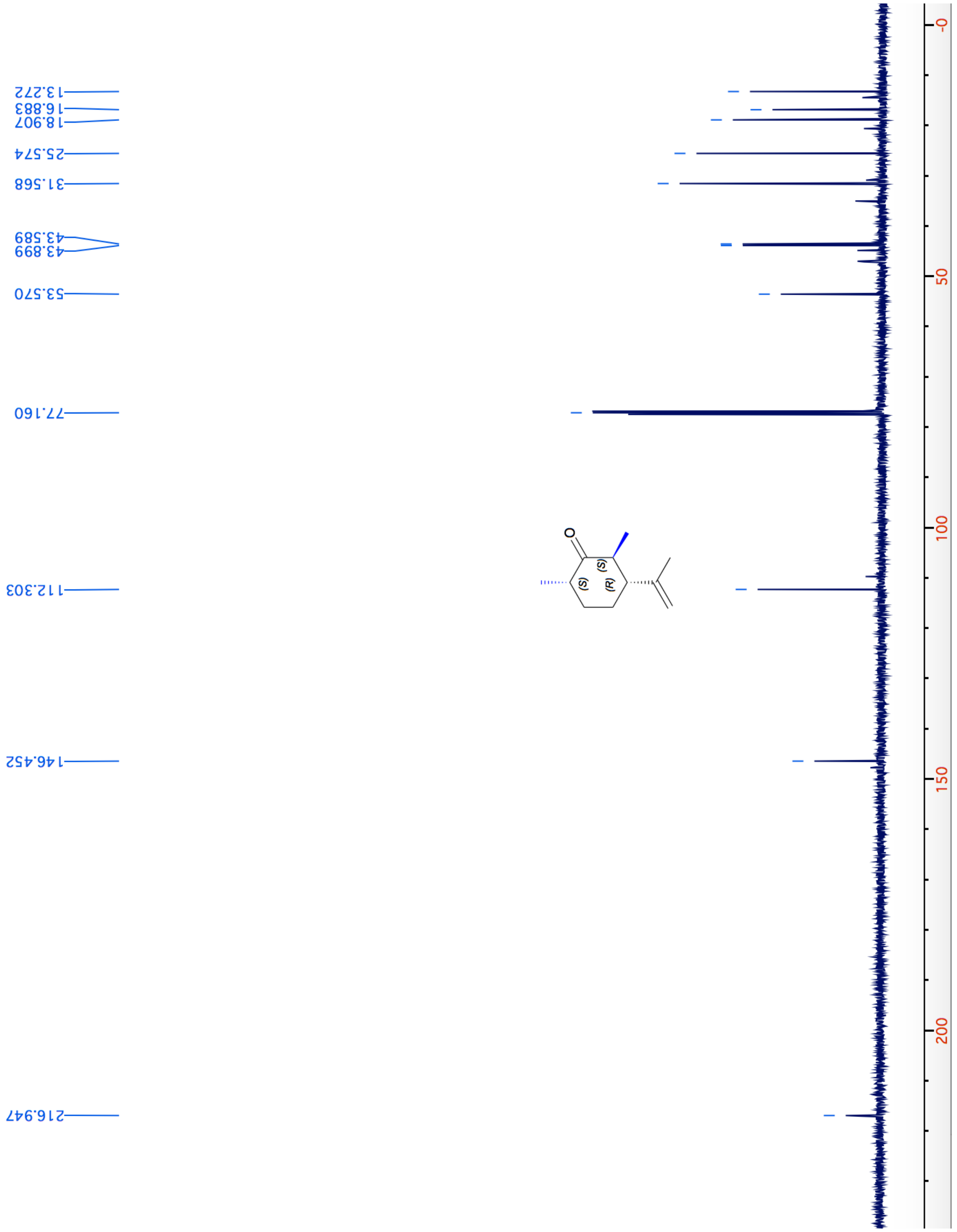
13.272
16.883
18.907
25.574
31.568
43.589
43.899
53.570
77.160
112.303
146.452
216.947
0
50
100
150
200
(S)
(S)
(R)
O

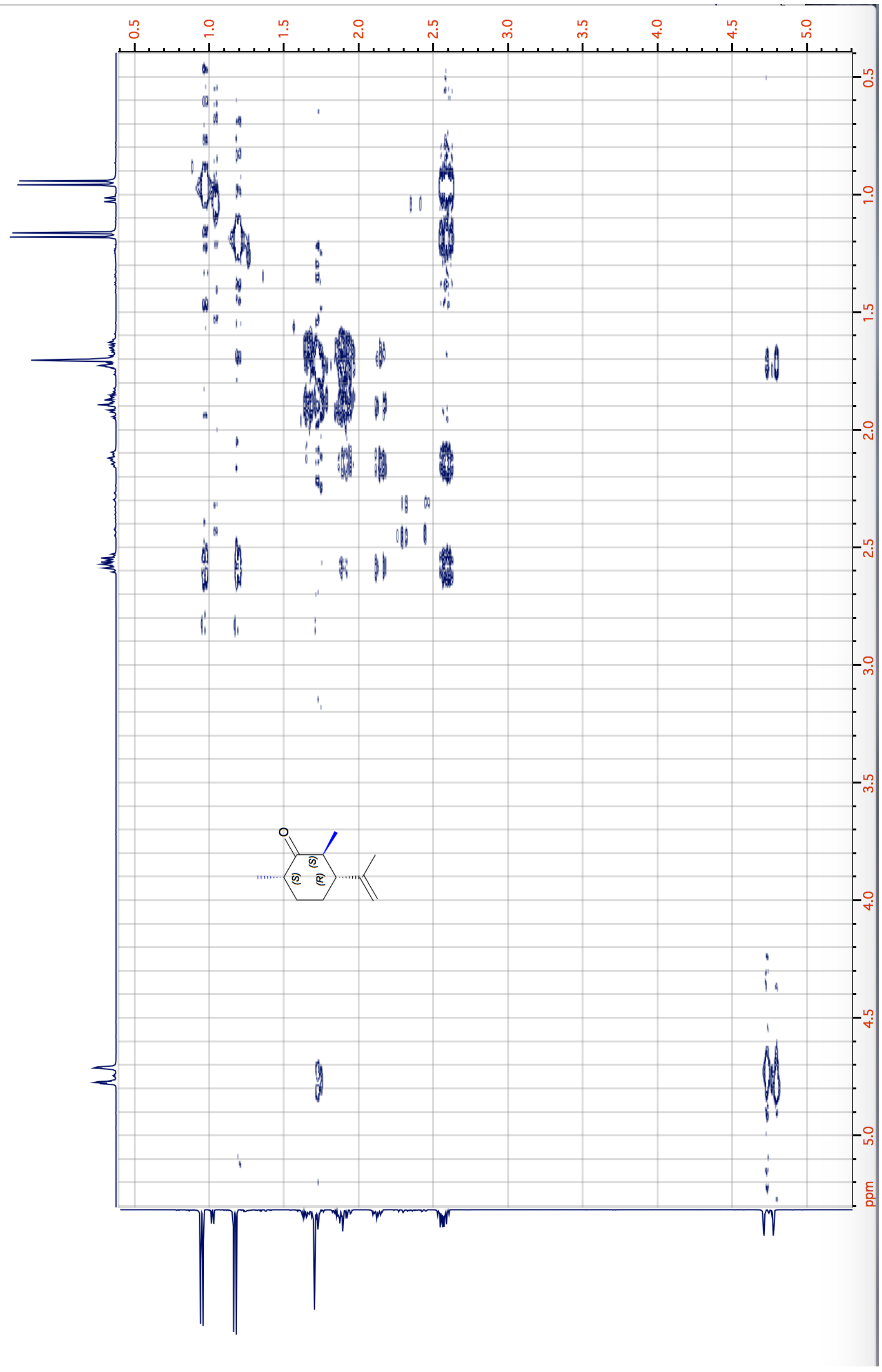
ppm
(S)
(S)
(R)
O

(2*R*,3*R*,6*R*)-2,6-Dimethyl-3-(prop-1-en-2-yl)cyclohexan-1-one, 13

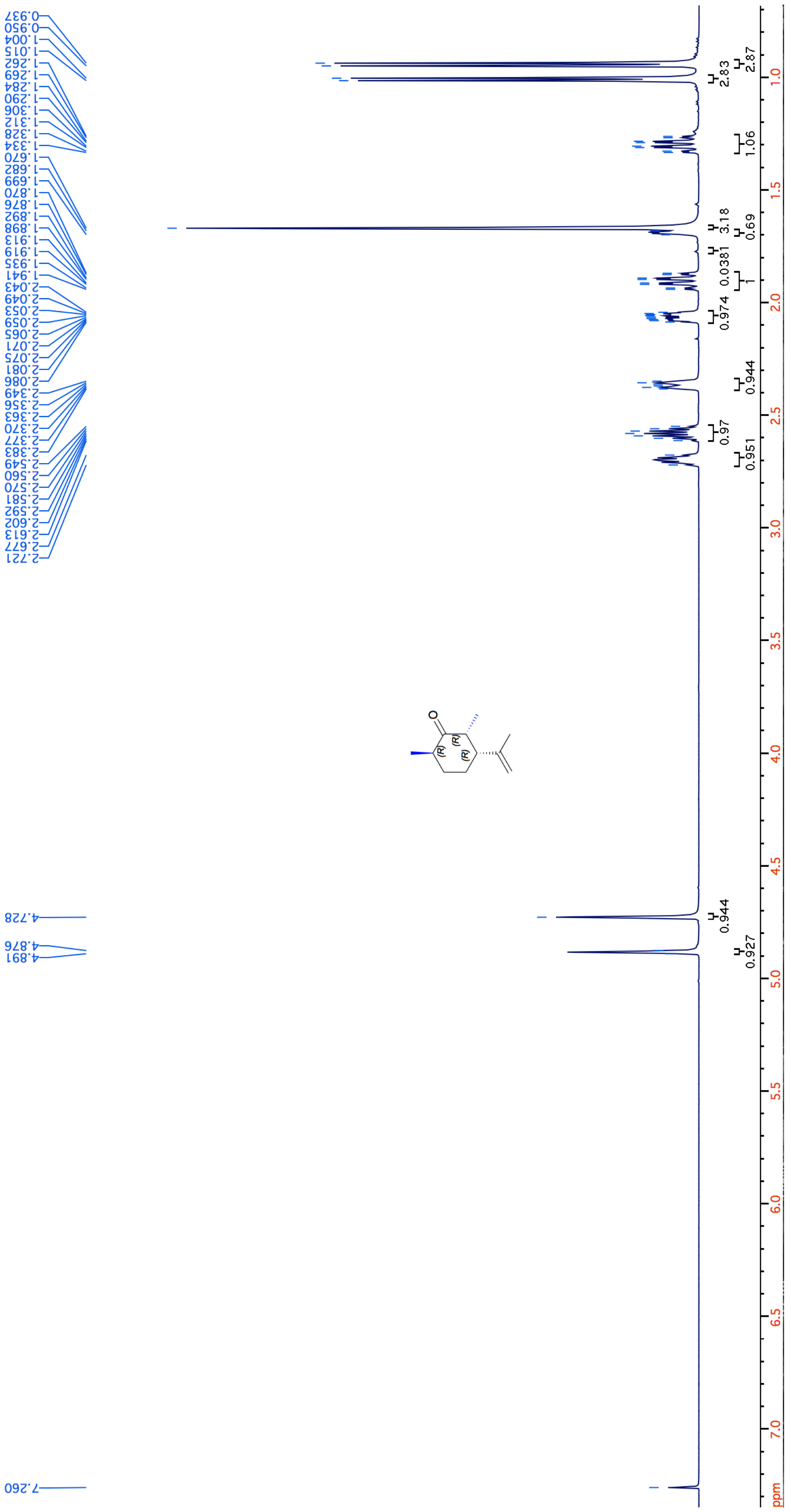

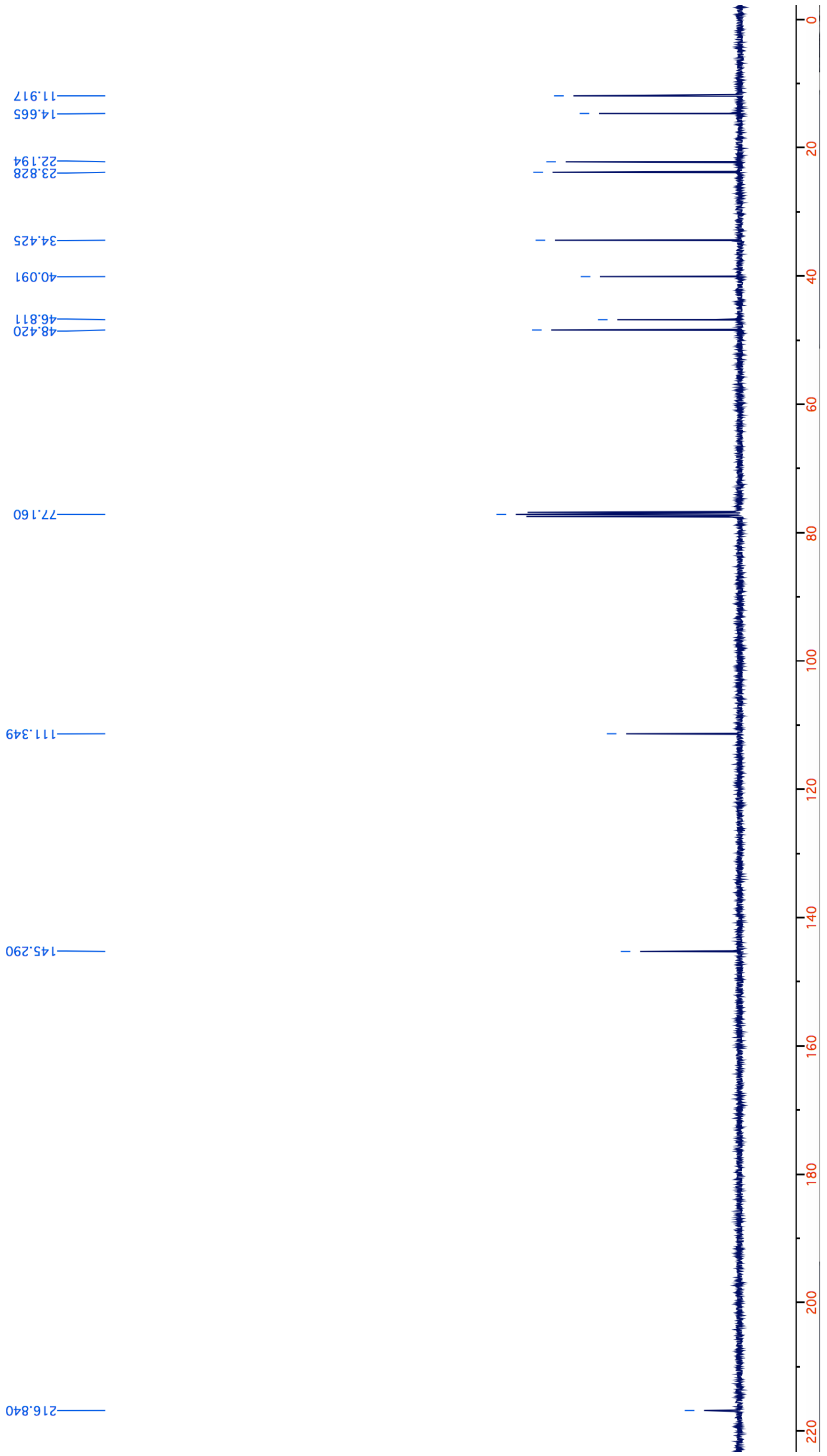
11.917
14.665
22.194
23.828
34.425
40.091
46.811
48.420
77.160
111.349
145.290
216.840
0
20
40
60
80
100
120
140
160
180
200
220

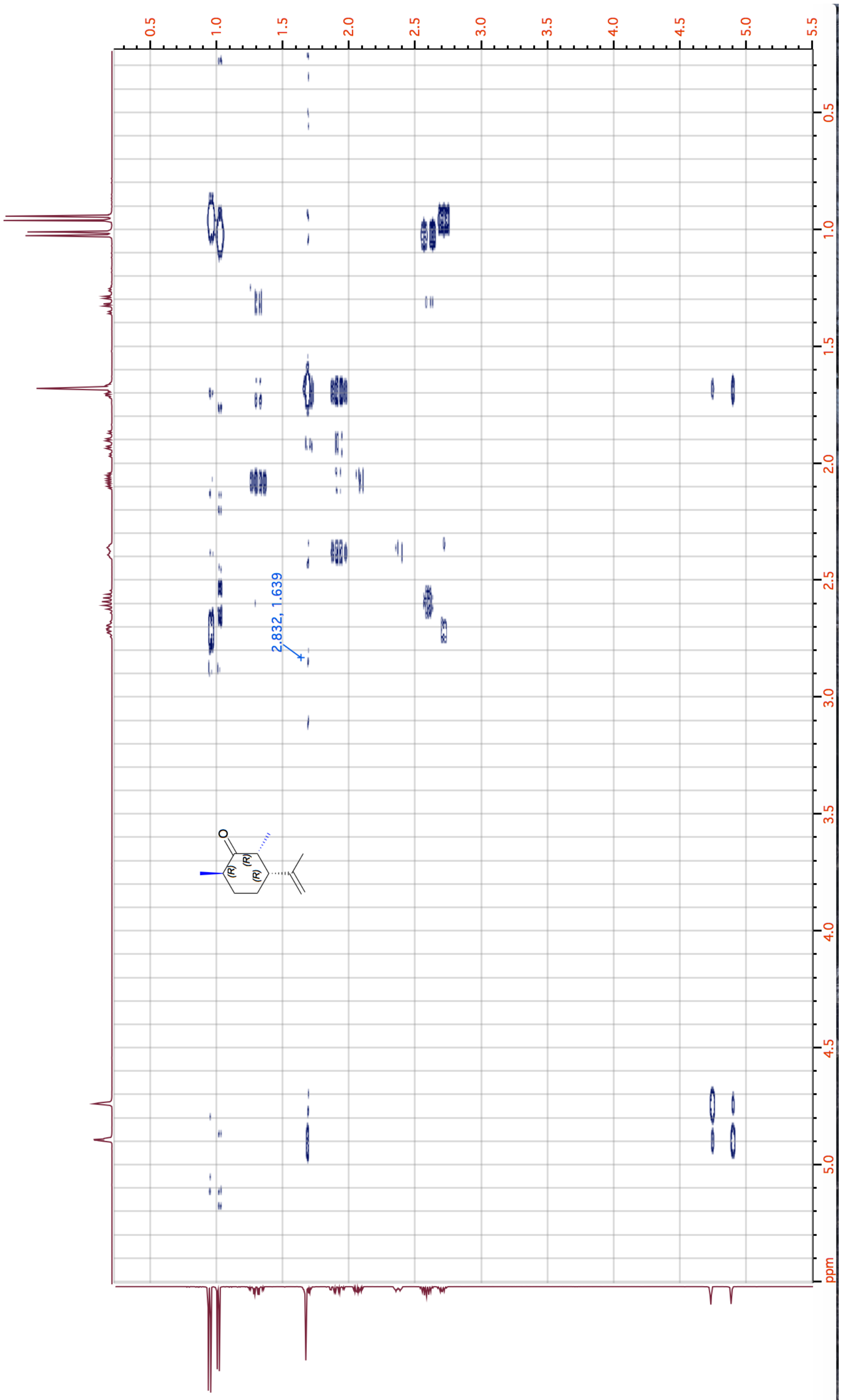
2.832, 1.639
ppm
(R)
(R)
(R)
O

(2*R*,3*R*,6*S*)-2,6-Dimethyl-3-(prop-1-en-2-yl)cyclohexan-1-one, 14

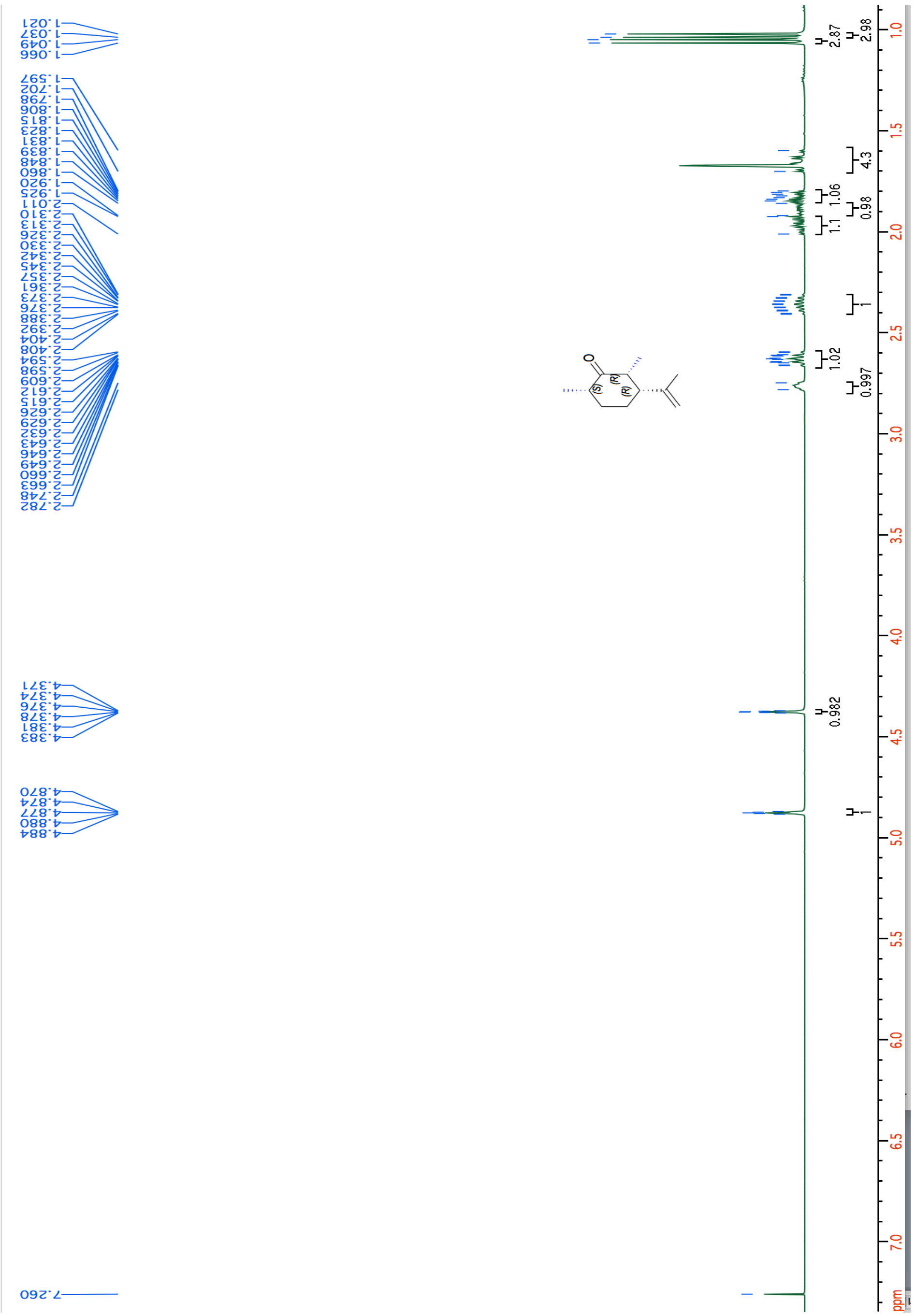

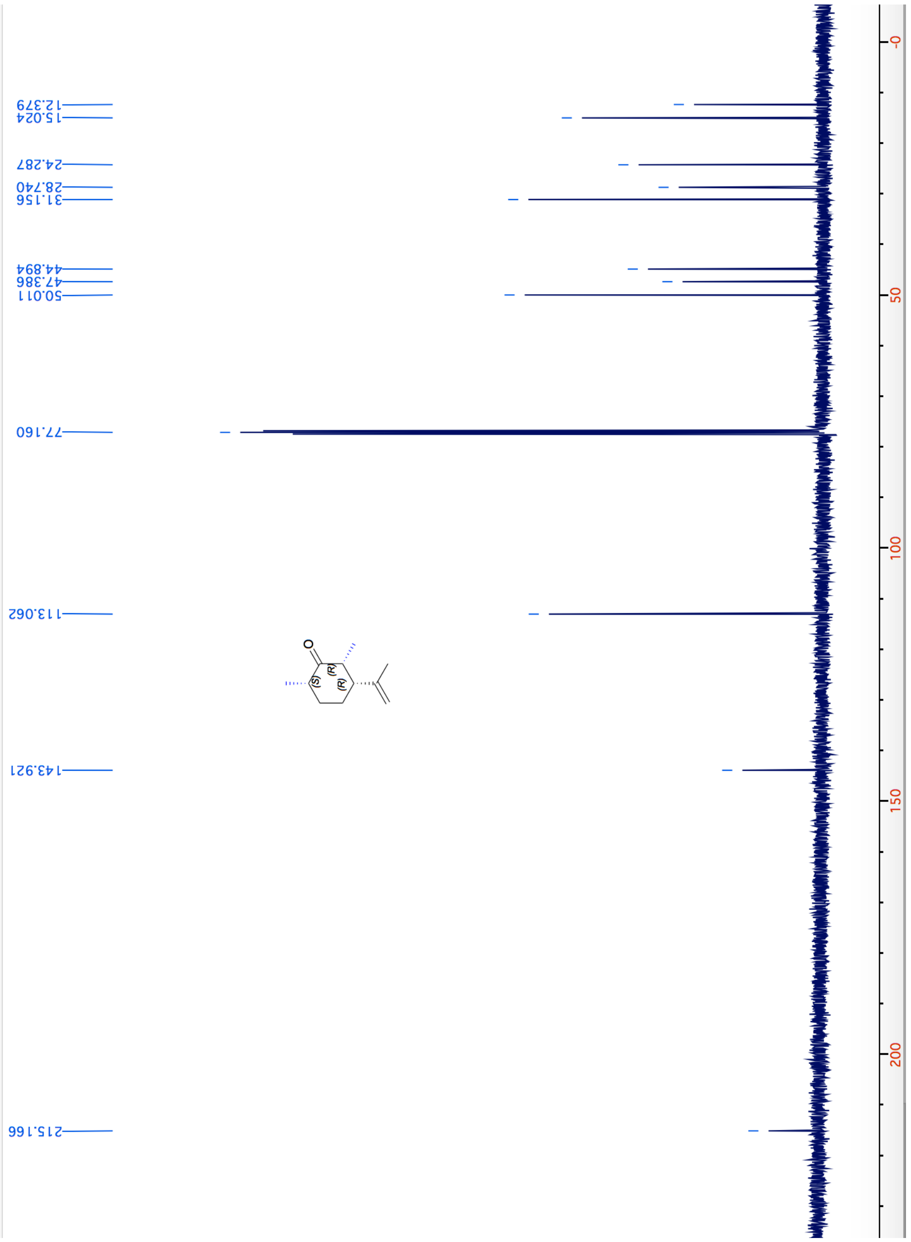
12.379
15.024
24.287
28.740
31.156
44.894
47.386
50.011
77.160
113.062
143.921
215.166
(S)
(R)
(R)
O
0
50
100
150
200

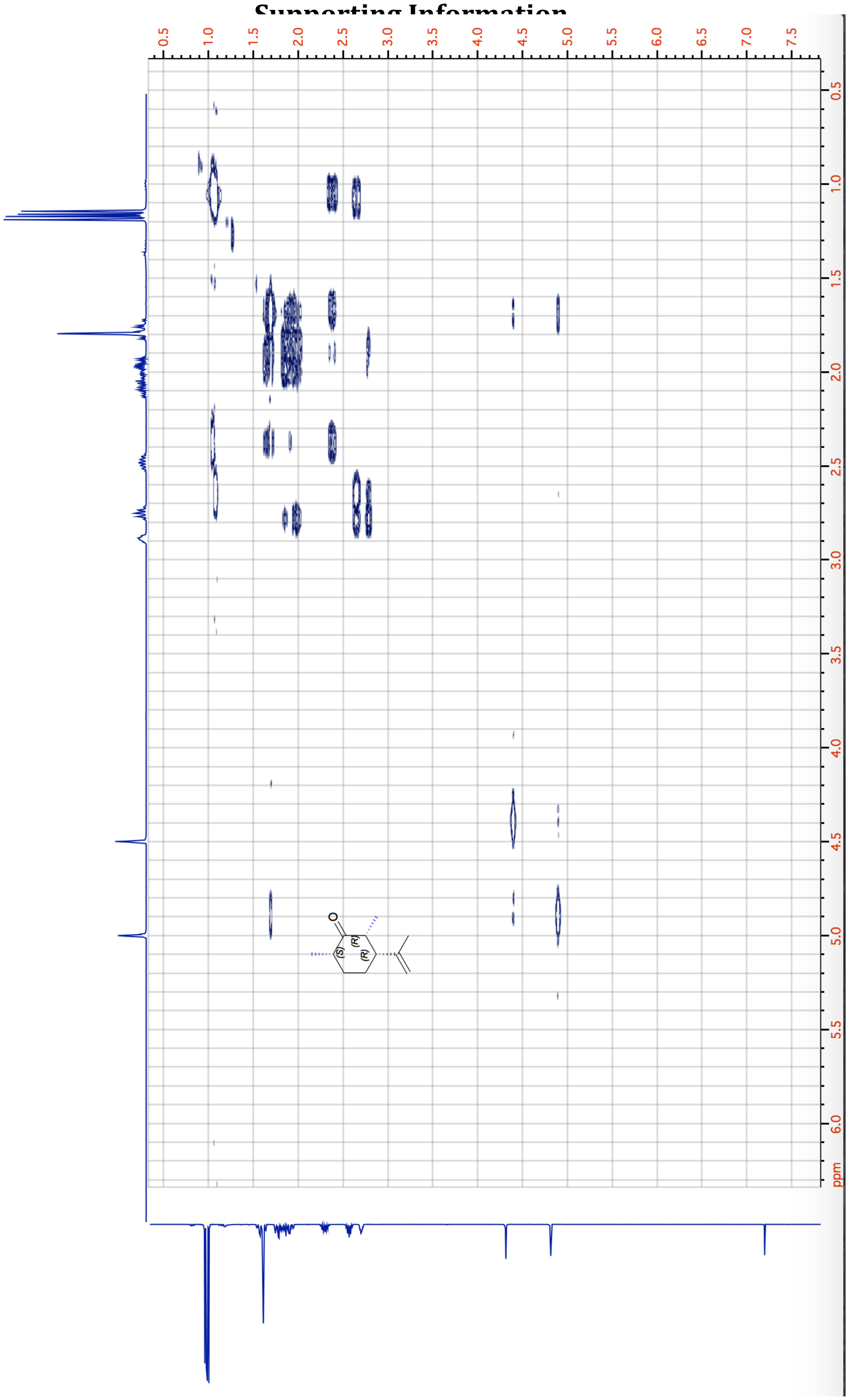

(2*S*,3*S*,6*R*)-2-Hydroxy-6-methyl-3-(prop-1-en-2-yl)cyclohexan-1-one, 15

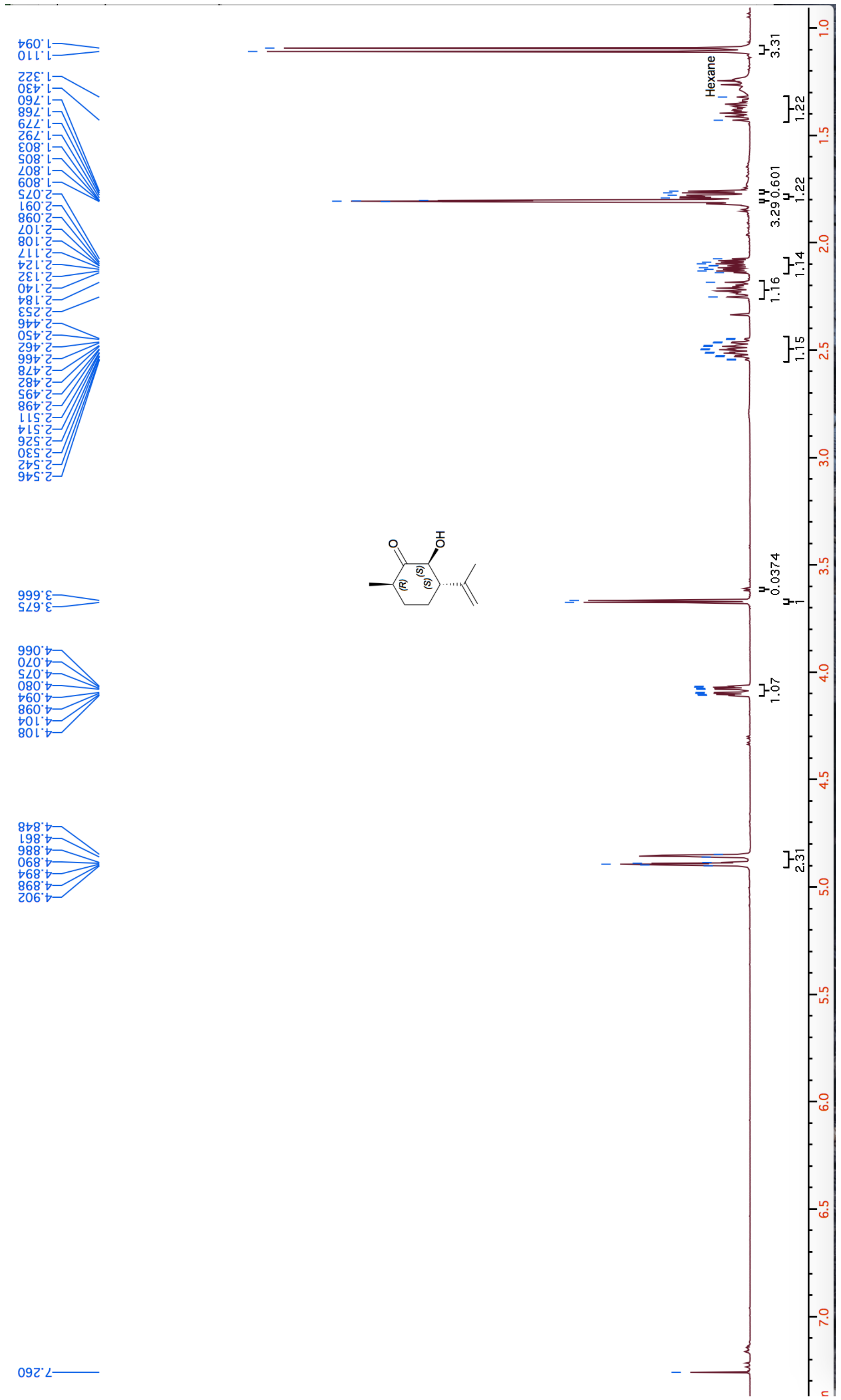

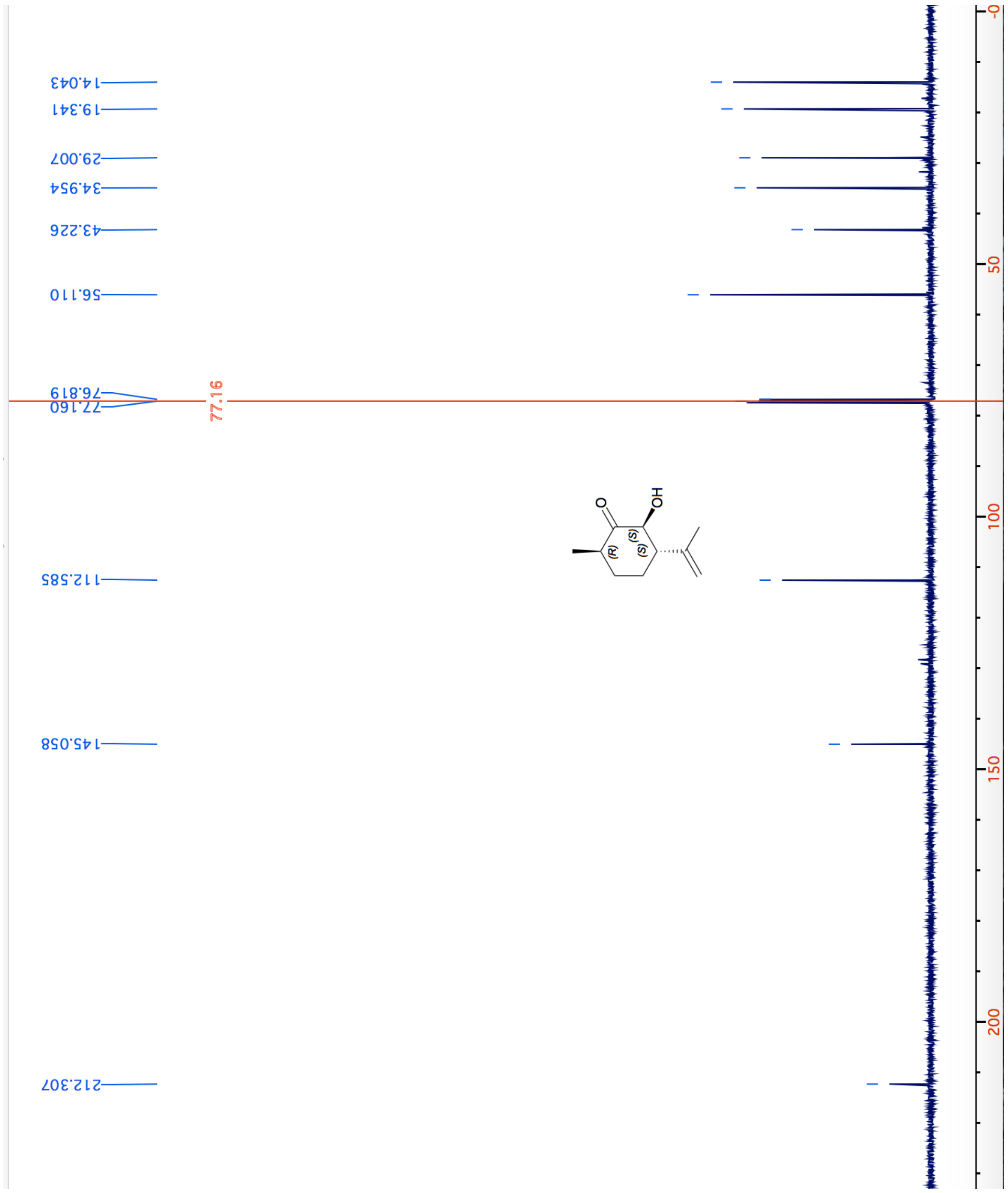
14.043
19.341
29.007
34.954
43.226
56.110
76.819
77.160
77.16
112.585
145.058
212.307
O
OH
(R)
(S)
(S)
0
50
100
150
200

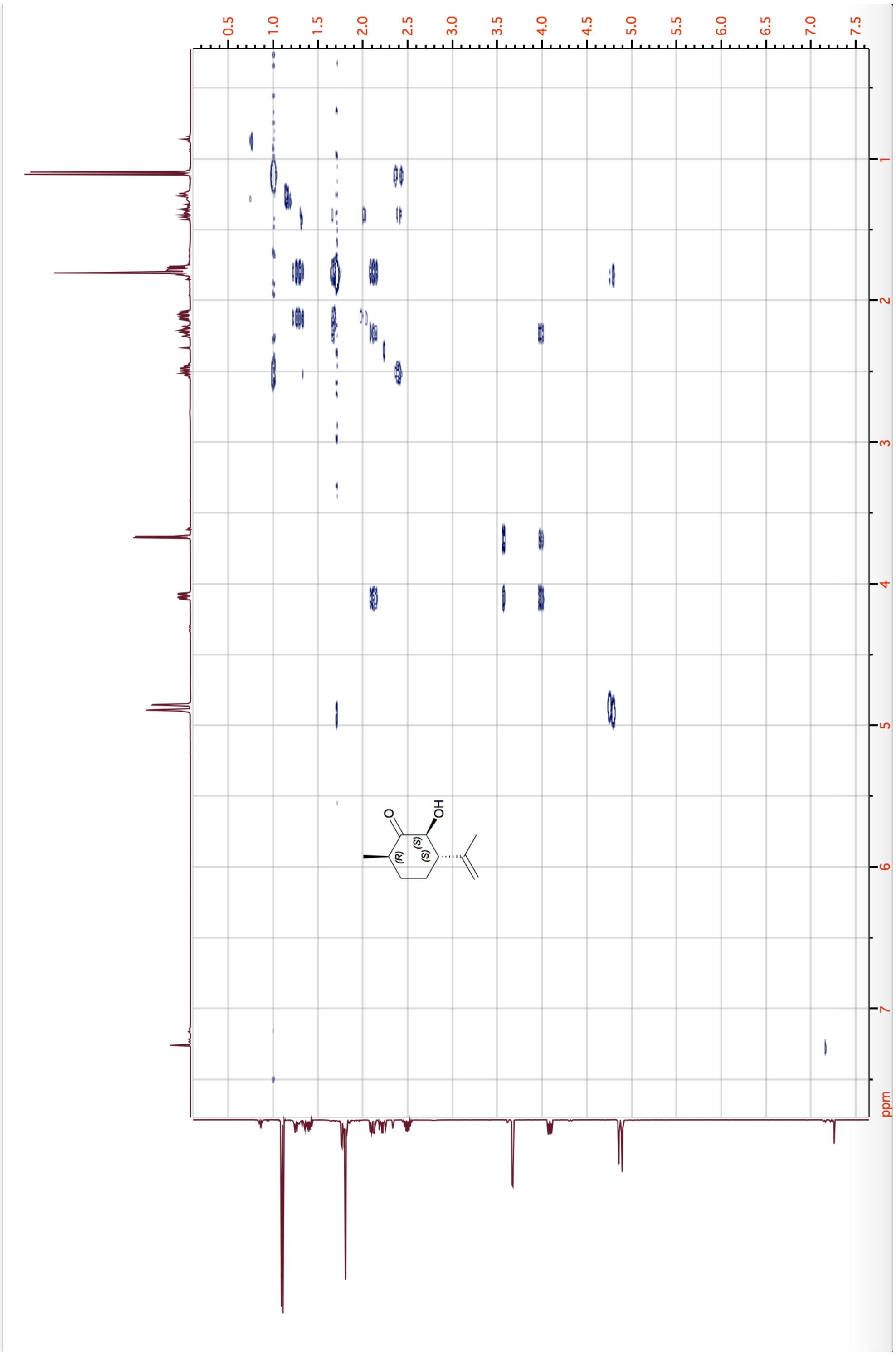
0.5
1.0
1.5
2.0
2.5
3.0
3.5
4.0
4.5
5.0
5.5
6.0
6.5
7.0
7.5
1
2
3
4
5
6
7
ppm
O
OH
(R)
(S)
(S)

(2*R*,3*S*,6*R*)-2-Hydroxy-6-methyl-3-(prop-1-en-2-yl)cyclohexan-1-one, 17

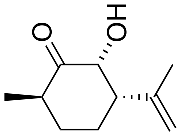

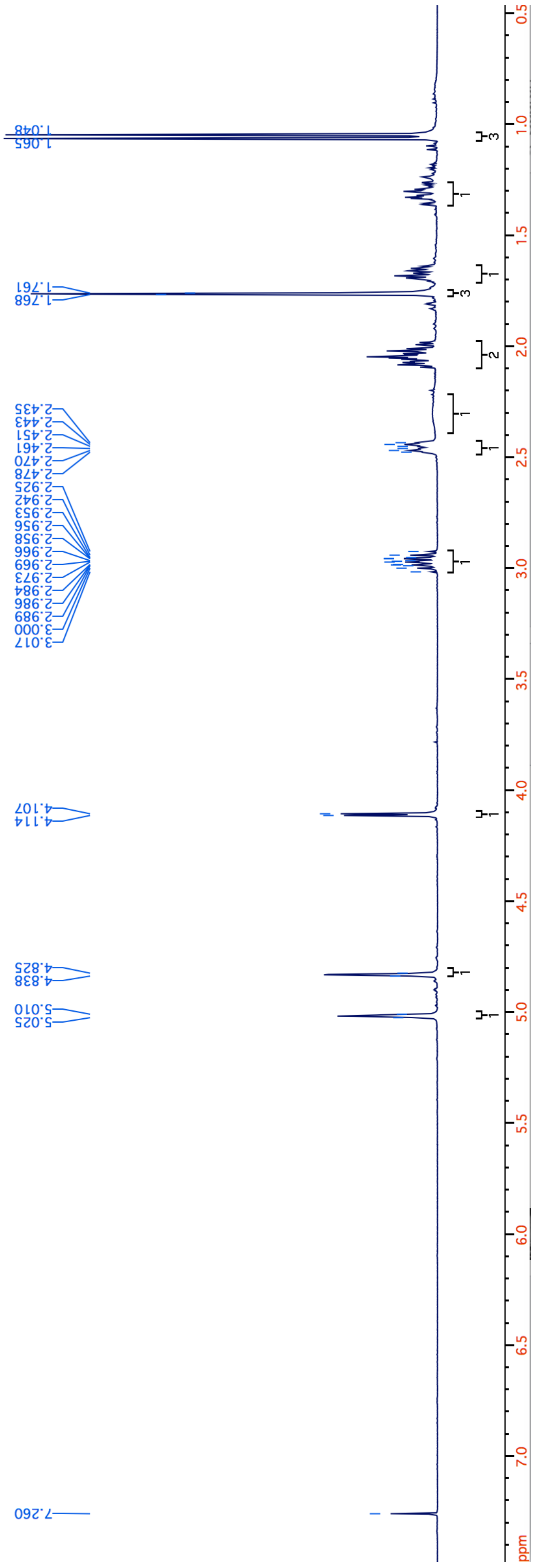

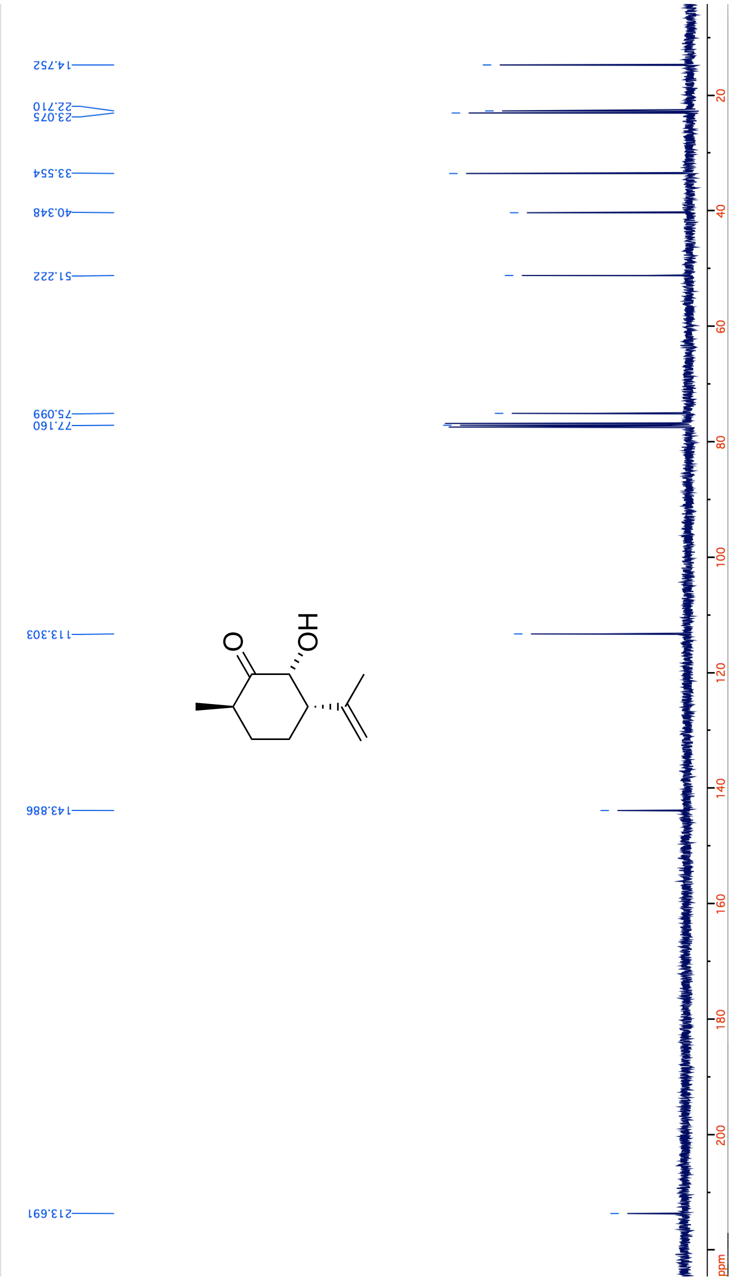
14.752
22.710
23.075
33.554
40.348
51.222
75.099
77.160
113.303
143.886
213.691
OH
O
20
40
60
80
100
120
140
160
180
200
ppm

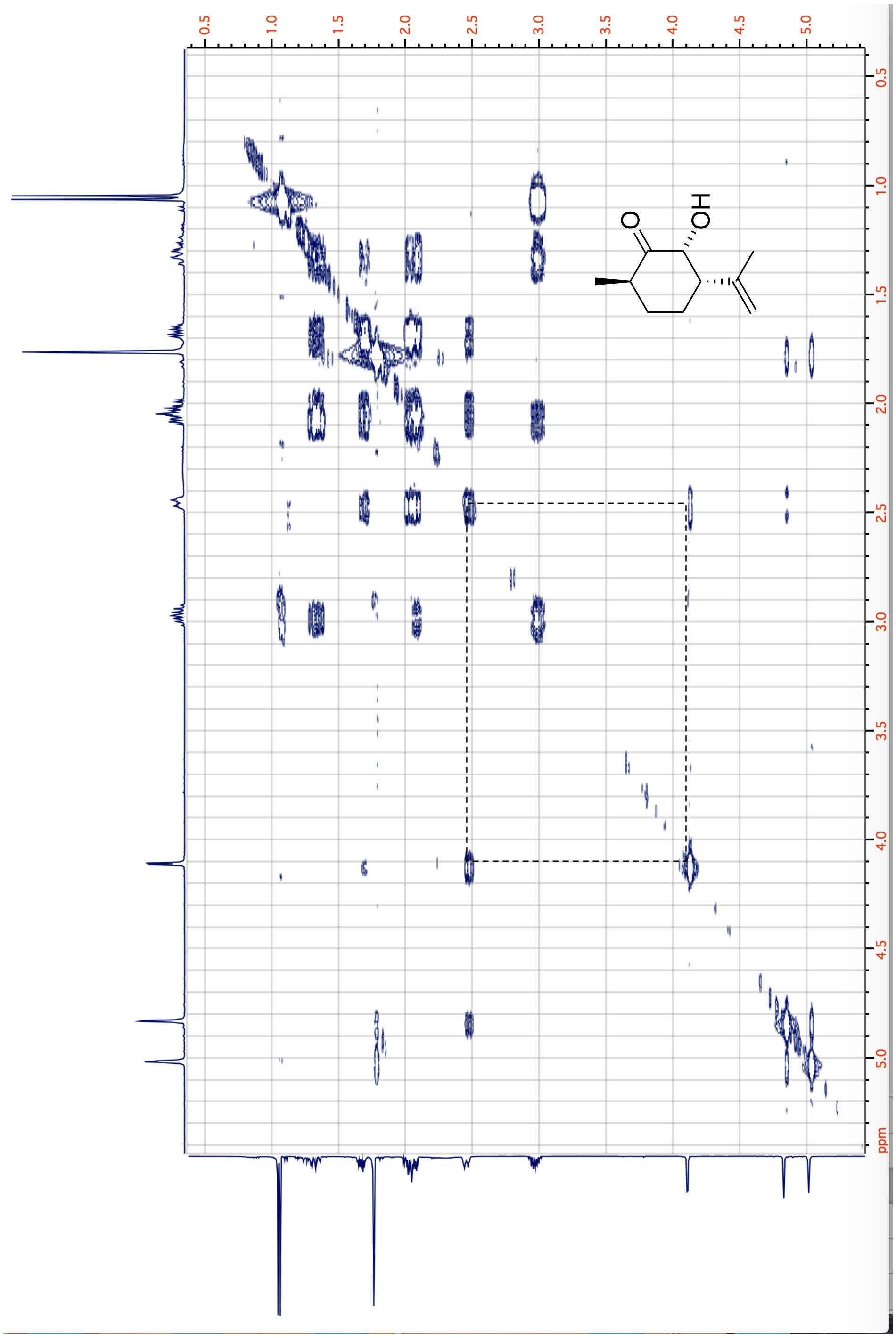

Supporting Information

(2*R*,3*R*,6*S*)-2-Hydroxy-6-methyl-3-(prop-1-en-2-yl)cyclohexan-1-one, 20

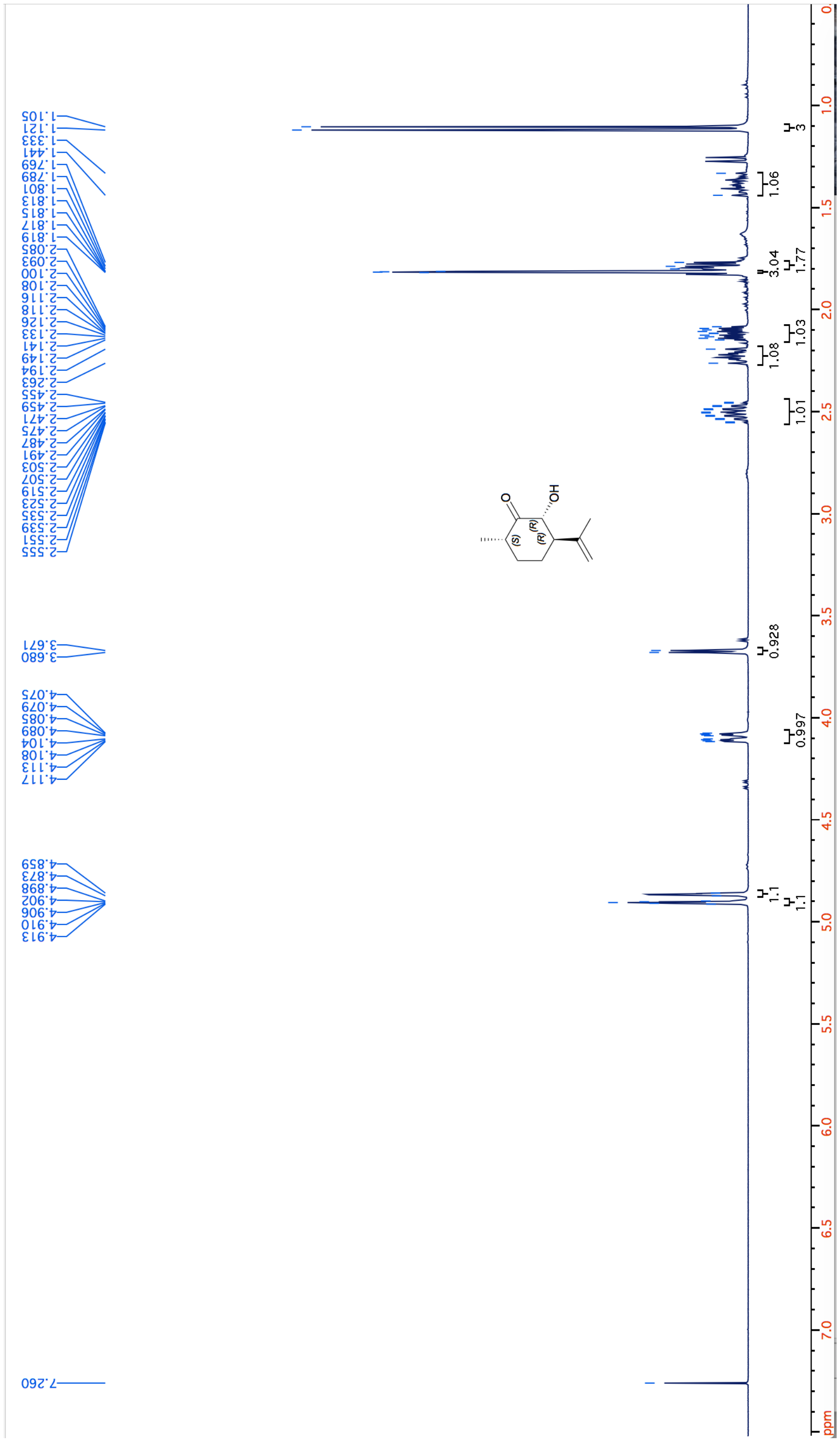

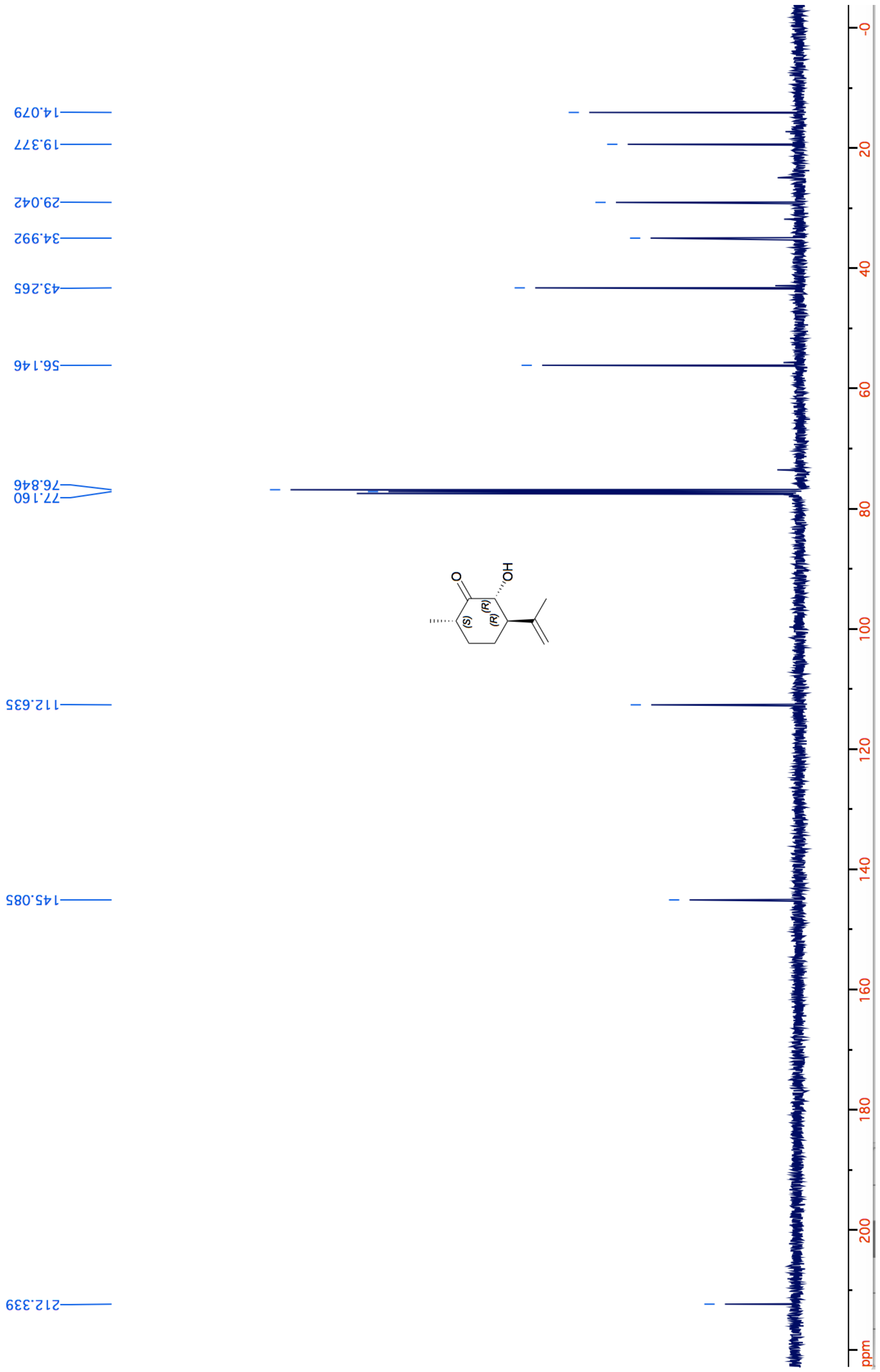
14.079
19.377
29.042
34.992
43.265
56.146
76.846
77.160
112.635
145.085
212.339
O
OH
(S)
(R)
(R)
0
20
40
60
80
100
120
140
160
180
200
ppm

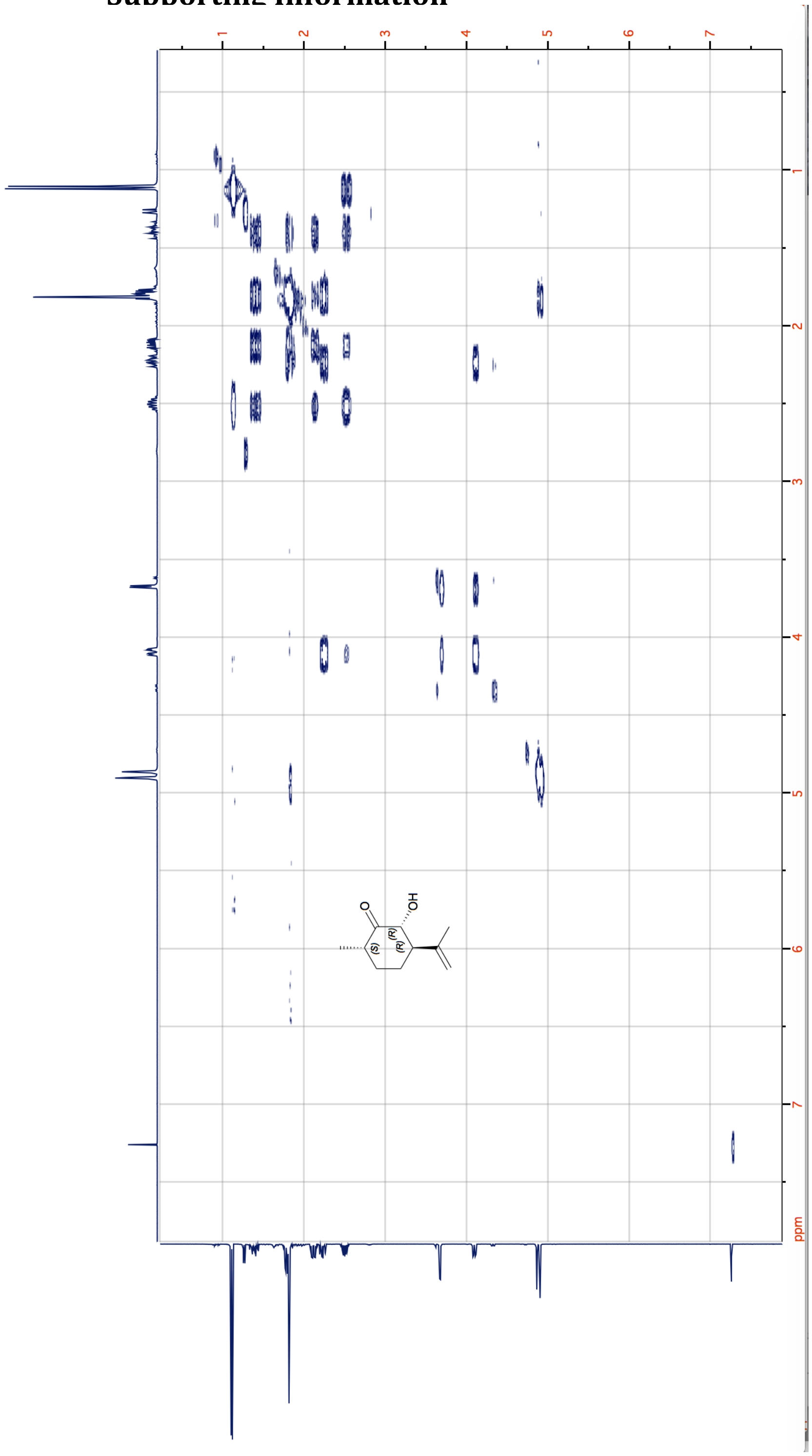
ppm
O
OH
(S)
(R)
(R)

(*R*)-2,3-Dimethyl-5-(prop-1-en-2-yl)cyclohex-2-en-1-one, 10

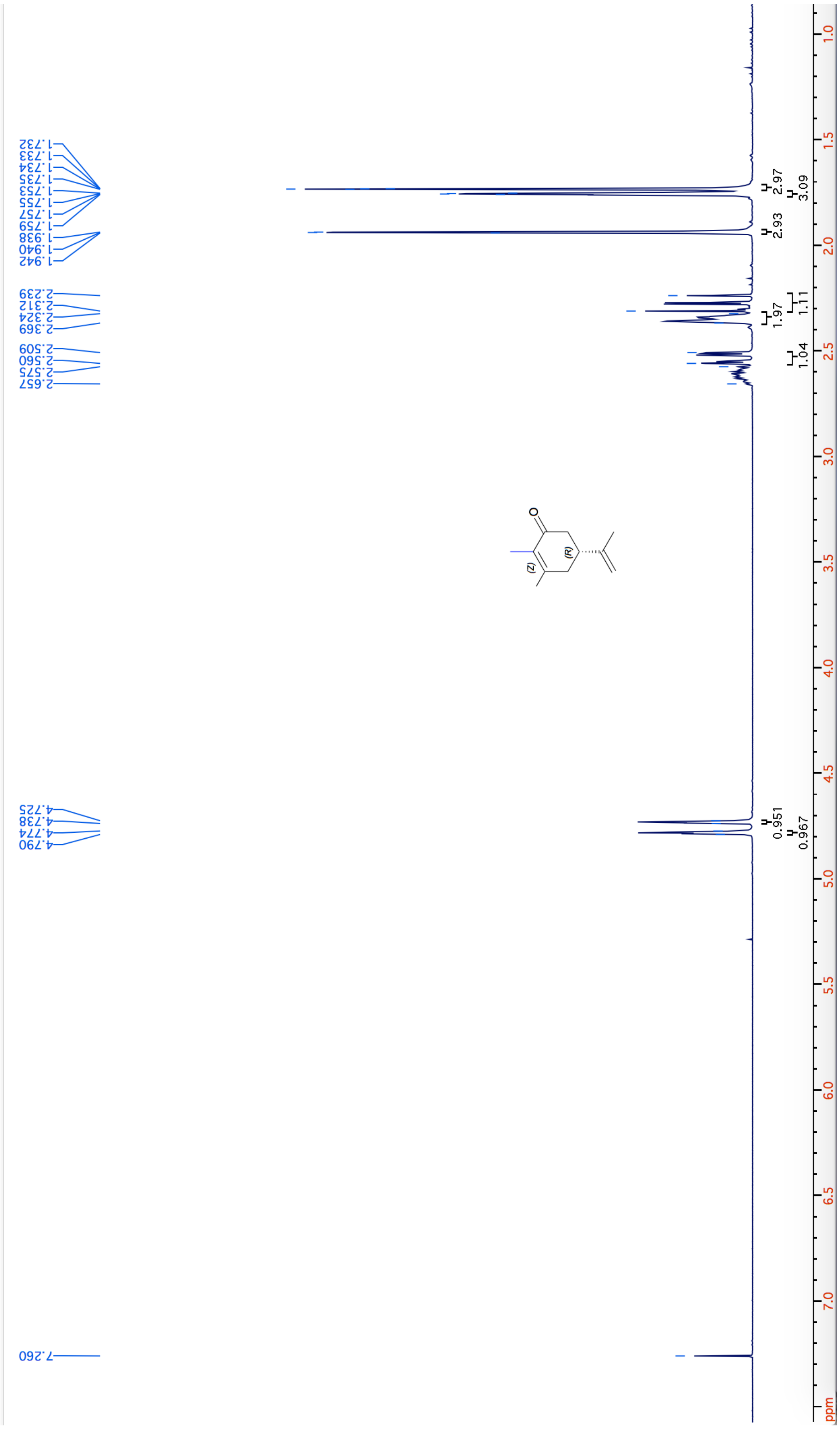

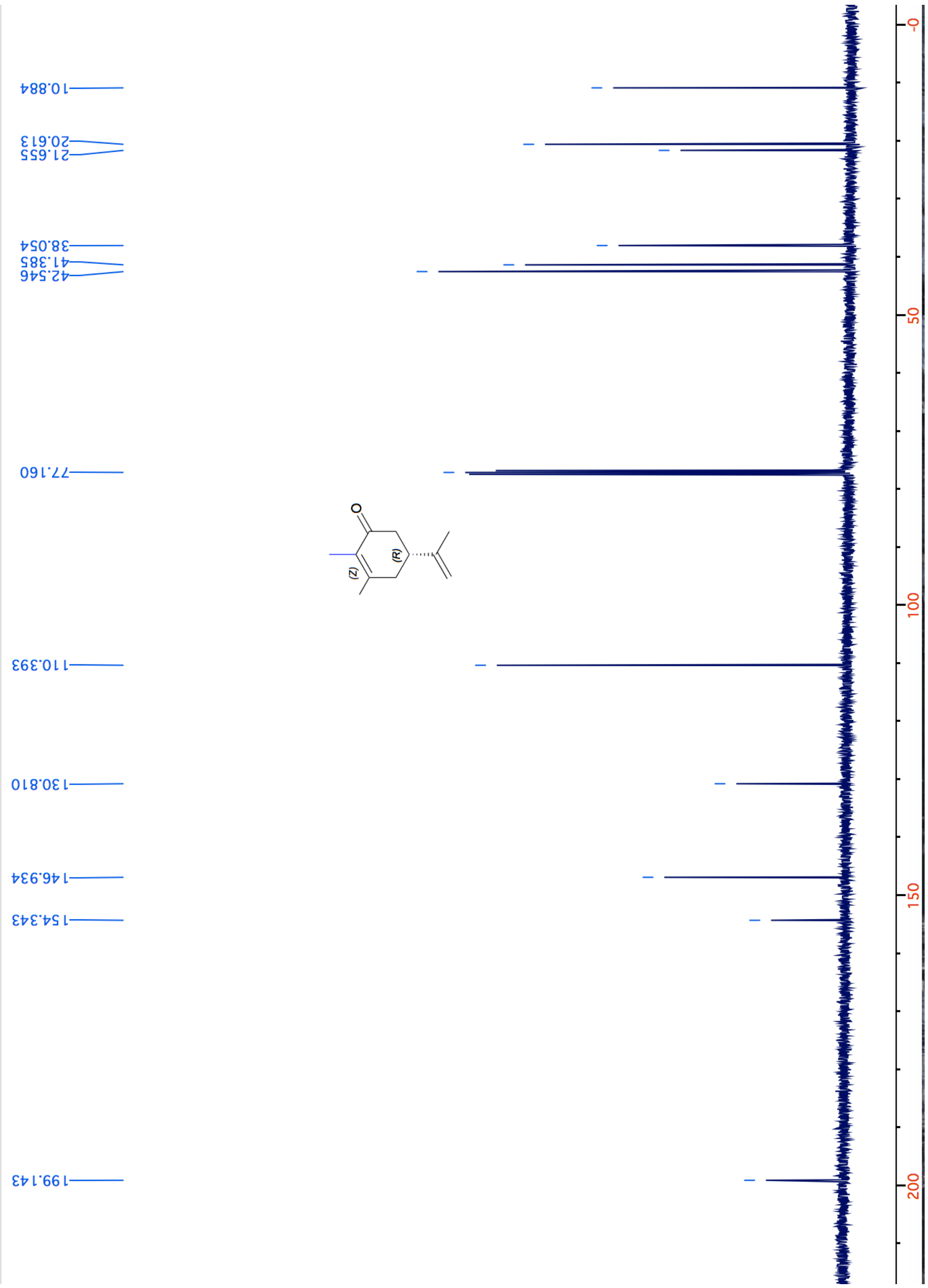
10.884
20.613
21.655
38.054
41.385
42.546
77.160
110.393
130.810
146.934
154.343
199.143
(Z)
(R)
O
0
50
100
150
200

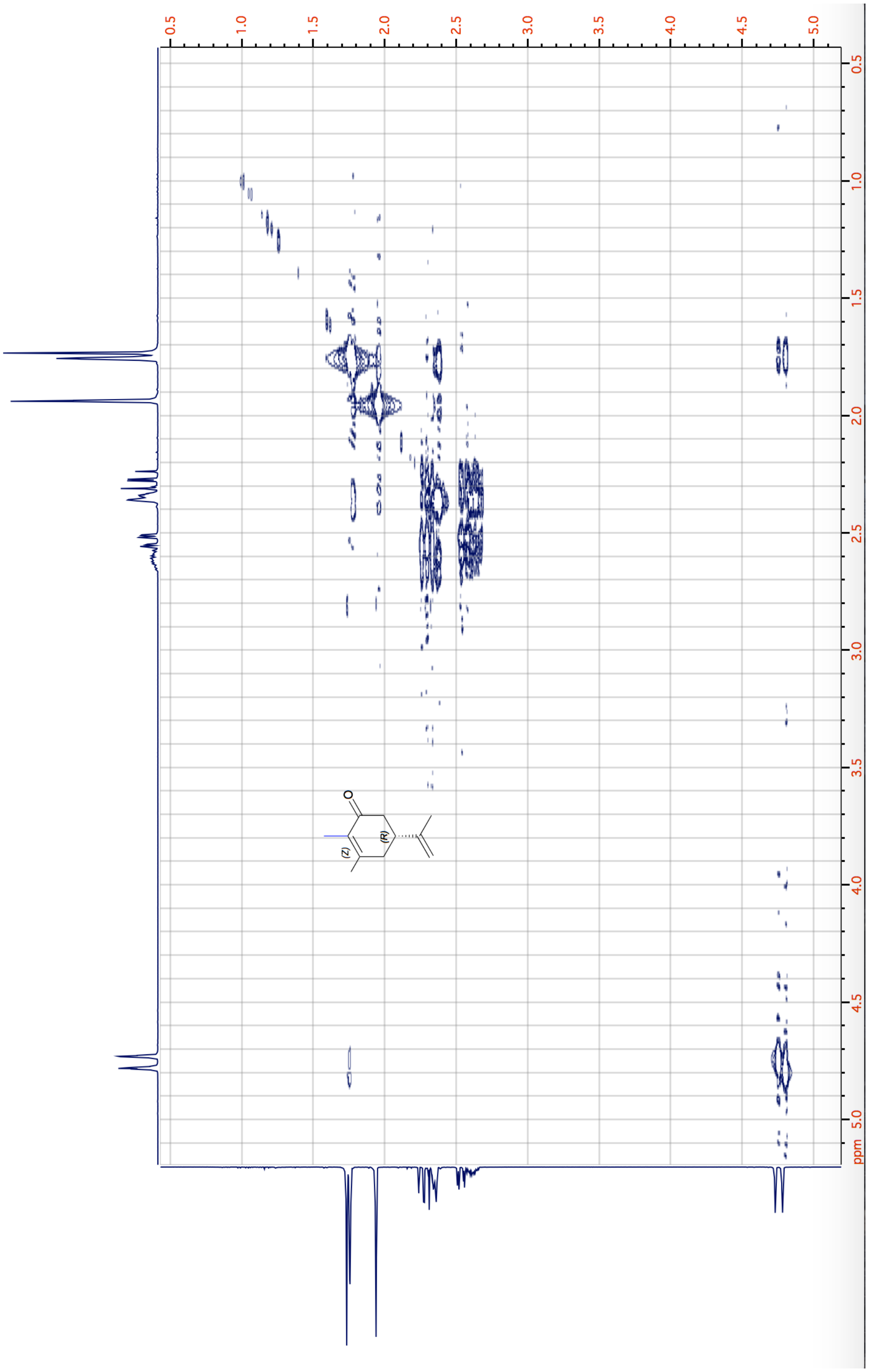

(2*S*,3*R*,5*R*)-2,3-Dimethyl-5-(prop-1-en-2-yl)cyclohexan-1-one, 22

(2*R*,3*R*,5*R*)-2,3-Dimethyl-5-(prop-1-en-2-yl)cyclohexan-1-one, 23

(2*R*,3*S*,5*R*)-2,3-Dimethyl-5-(prop-1-en-2-yl)cyclohexan-1-one, 24, and

(2*S*,3*S*,5*R*)-2,3-Dimethyl-5-(prop-1-en-2-yl)cyclohexan-1-one, 25 (mixture of isomers)

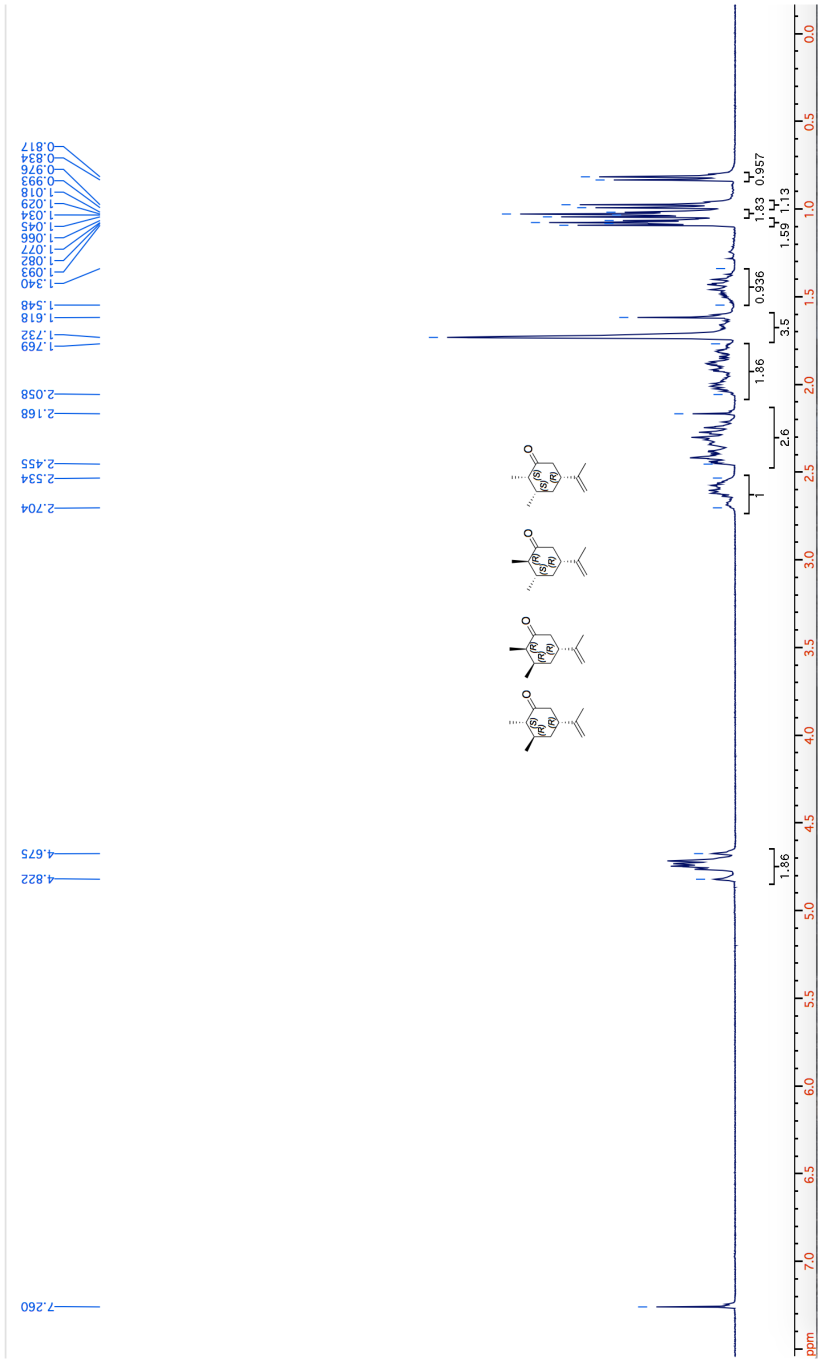

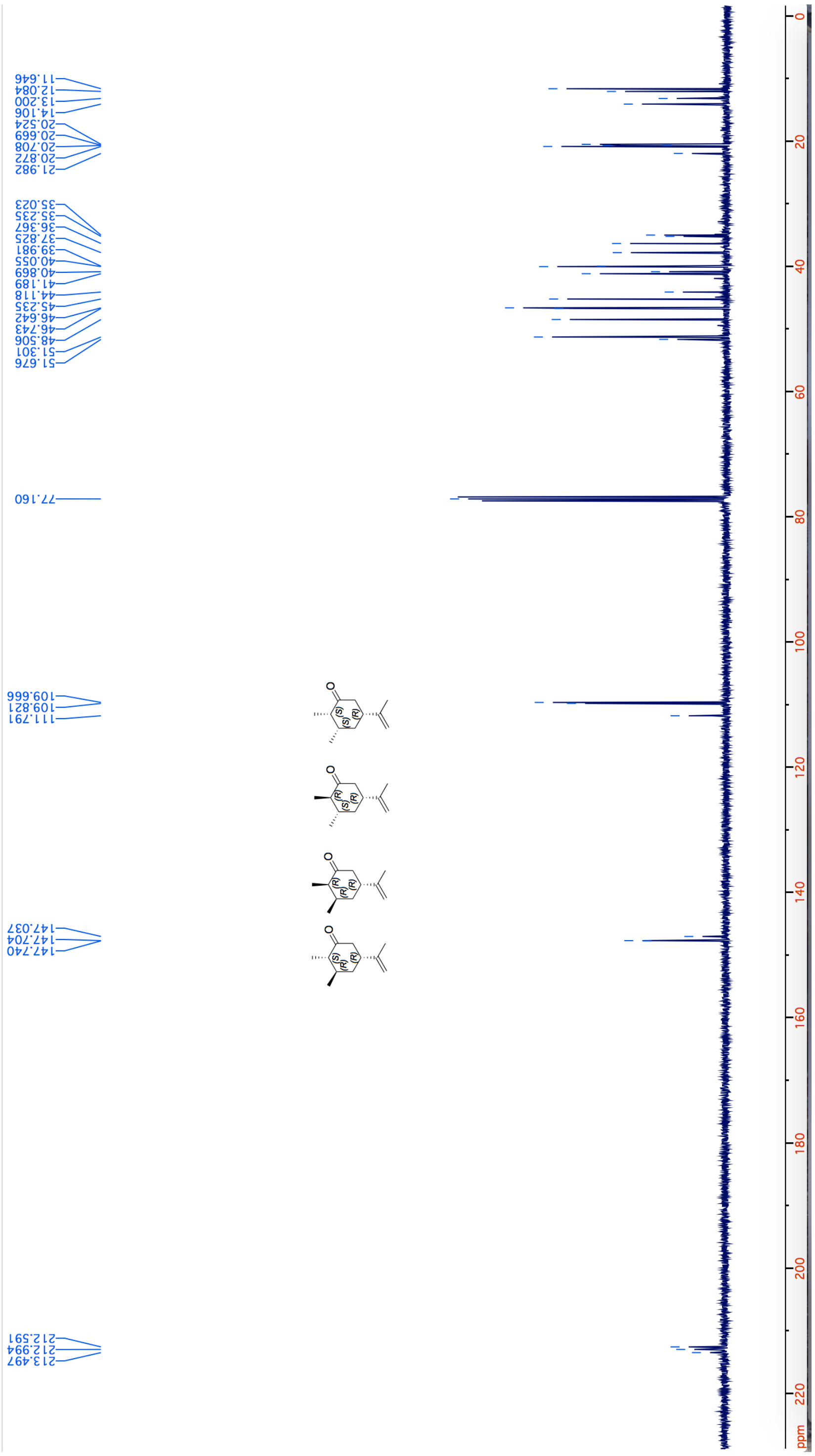
11.646
12.084
13.200
14.106
20.524
20.669
20.708
20.872
21.982
35.023
35.235
36.367
37.825
39.981
40.055
40.869
41.189
44.118
45.235
46.642
46.743
48.506
51.301
51.676
77.160
109.666
109.821
111.791
147.037
147.704
147.740
212.591
212.994
213.497
0
20
40
60
80
100
120
140
160
180
200
220
ppm

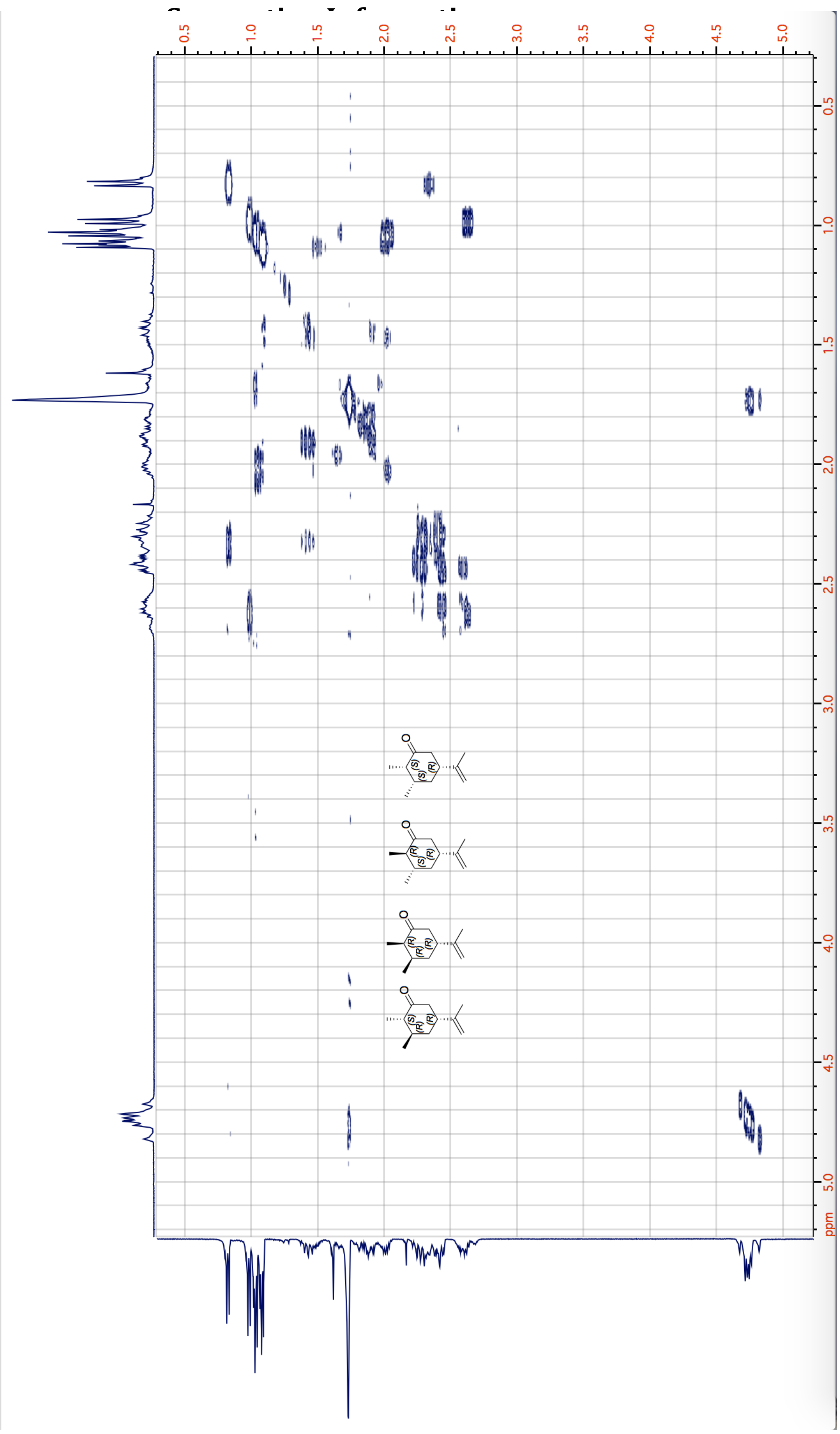

Supporting Information

(3*R*,6*S*,7*R*)-3,7-Dimethyl-4-(prop-1-en-2-yl)oxepan-2-one, 21

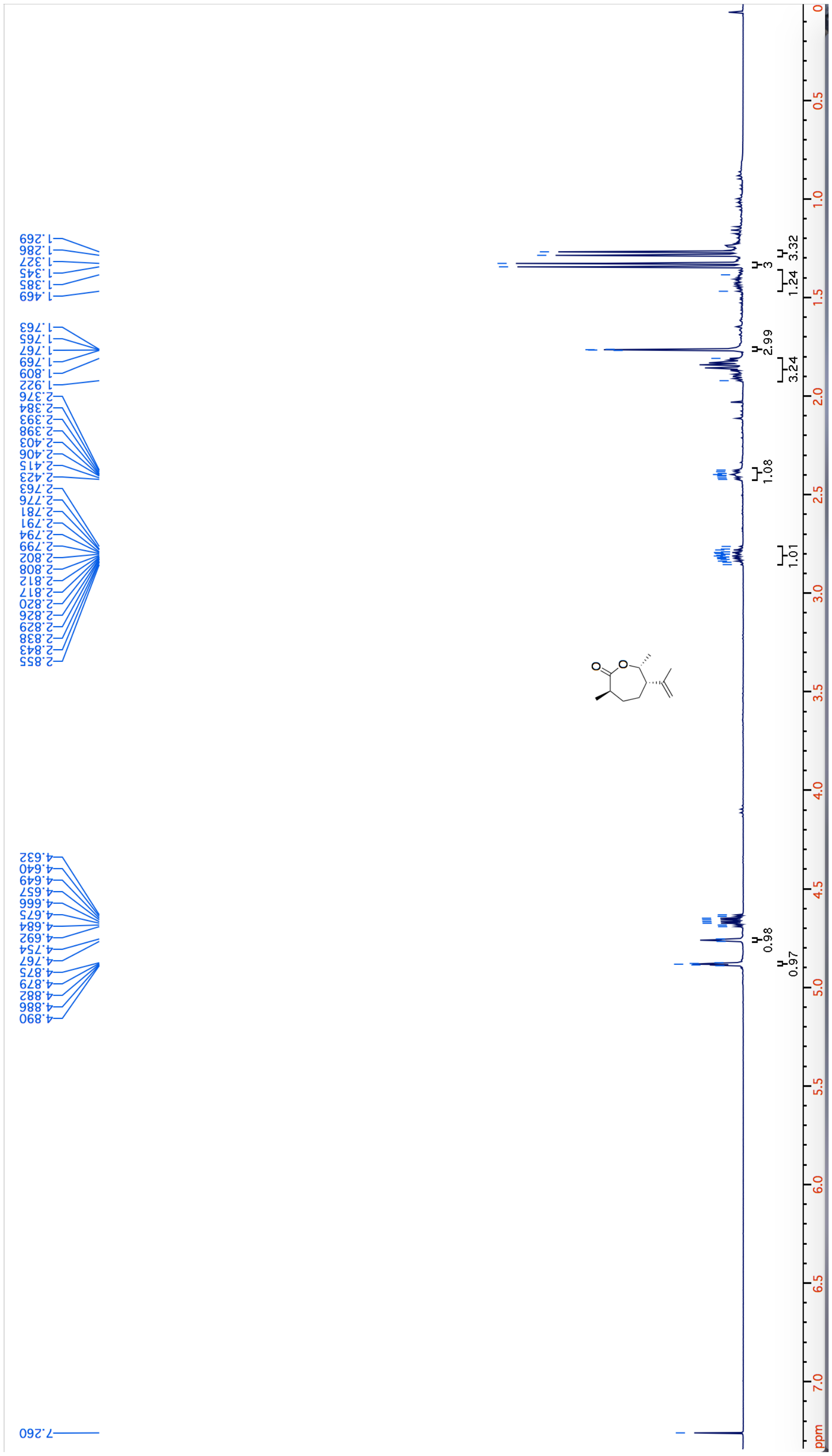

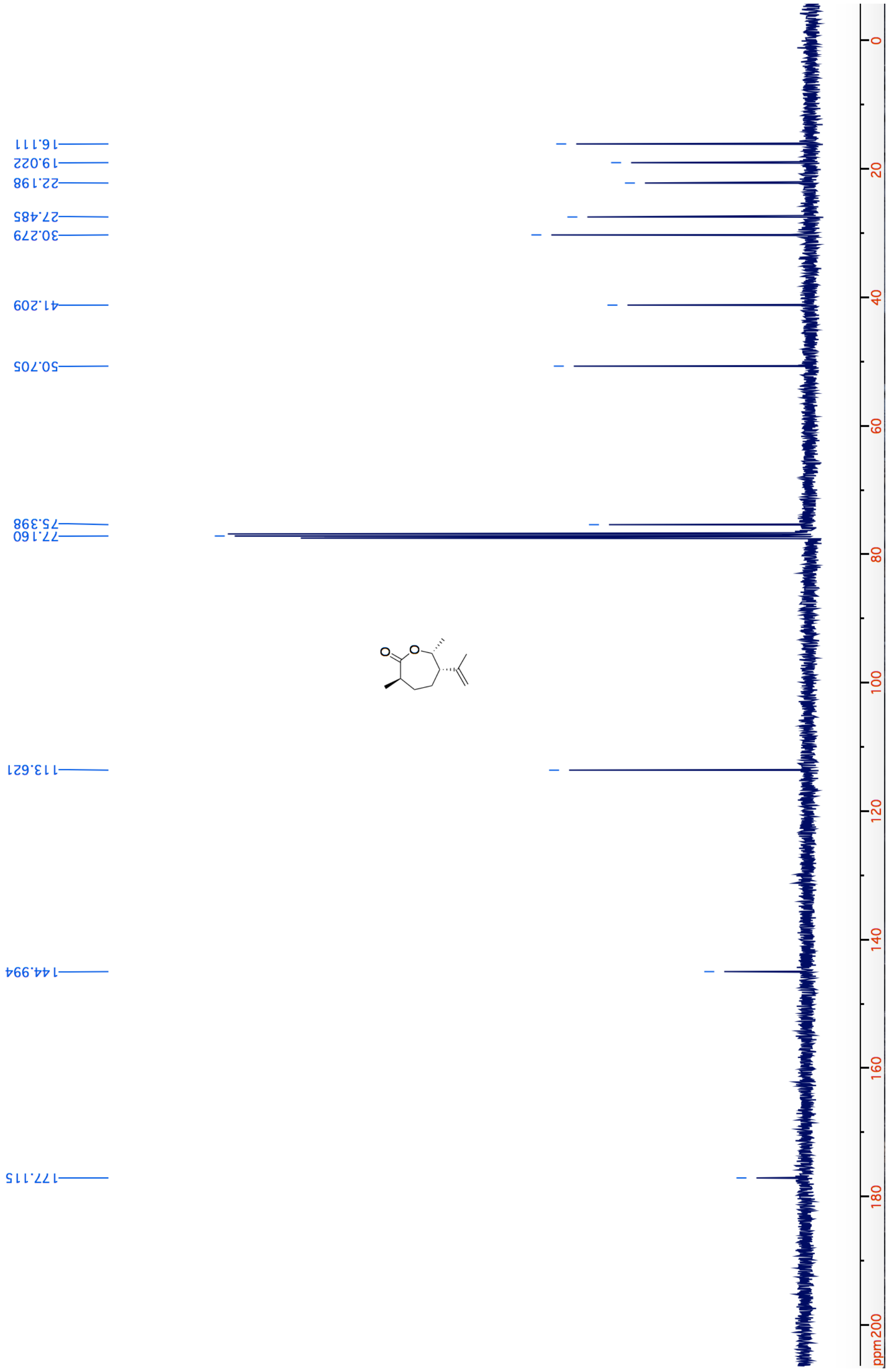
16.111
19.022
22.198
27.485
30.279
41.209
50.705
75.398
77.160
113.621
144.994
177.115
0
20
40
60
80
100
120
140
160
180
ppm 200

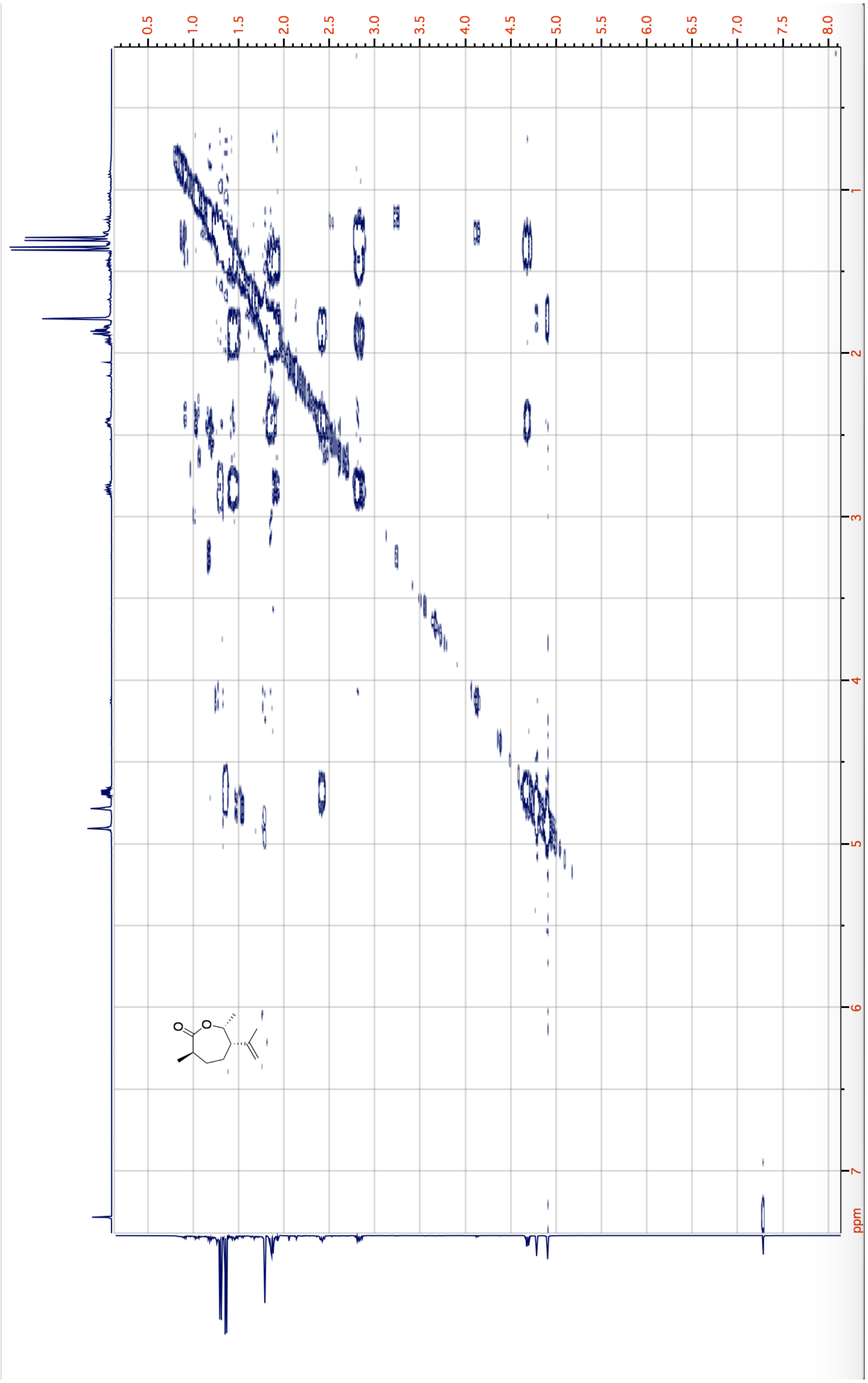

0.5
1.0
1.5
2.0
2.5
3.0
3.5
4.0
4.5
5.0
5.5
6.0
6.5
7.0
7.5
8.0
1
2
3
4
5
6
7
ppm

(3*R*,4*R*,6*S*)-3,4-Dimethyl-6-(prop-1-en-2-yl)oxepan-2-one, 26, and (3*S*,4*R*,6*S*)-3,4-Dimethyl-6-(prop-1-en-2-yl)oxepan-2-one, 27 (mixture of lactones)

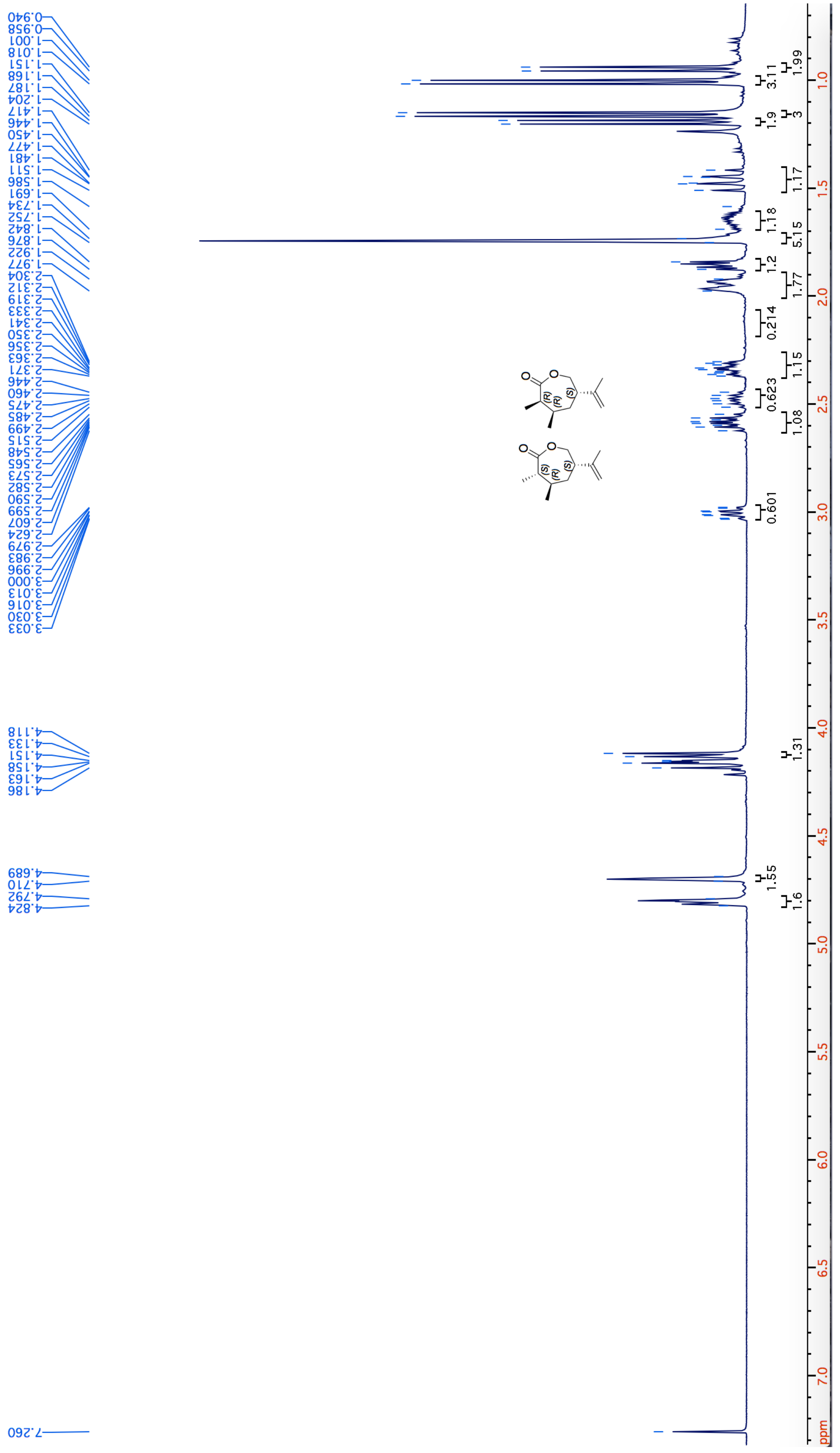

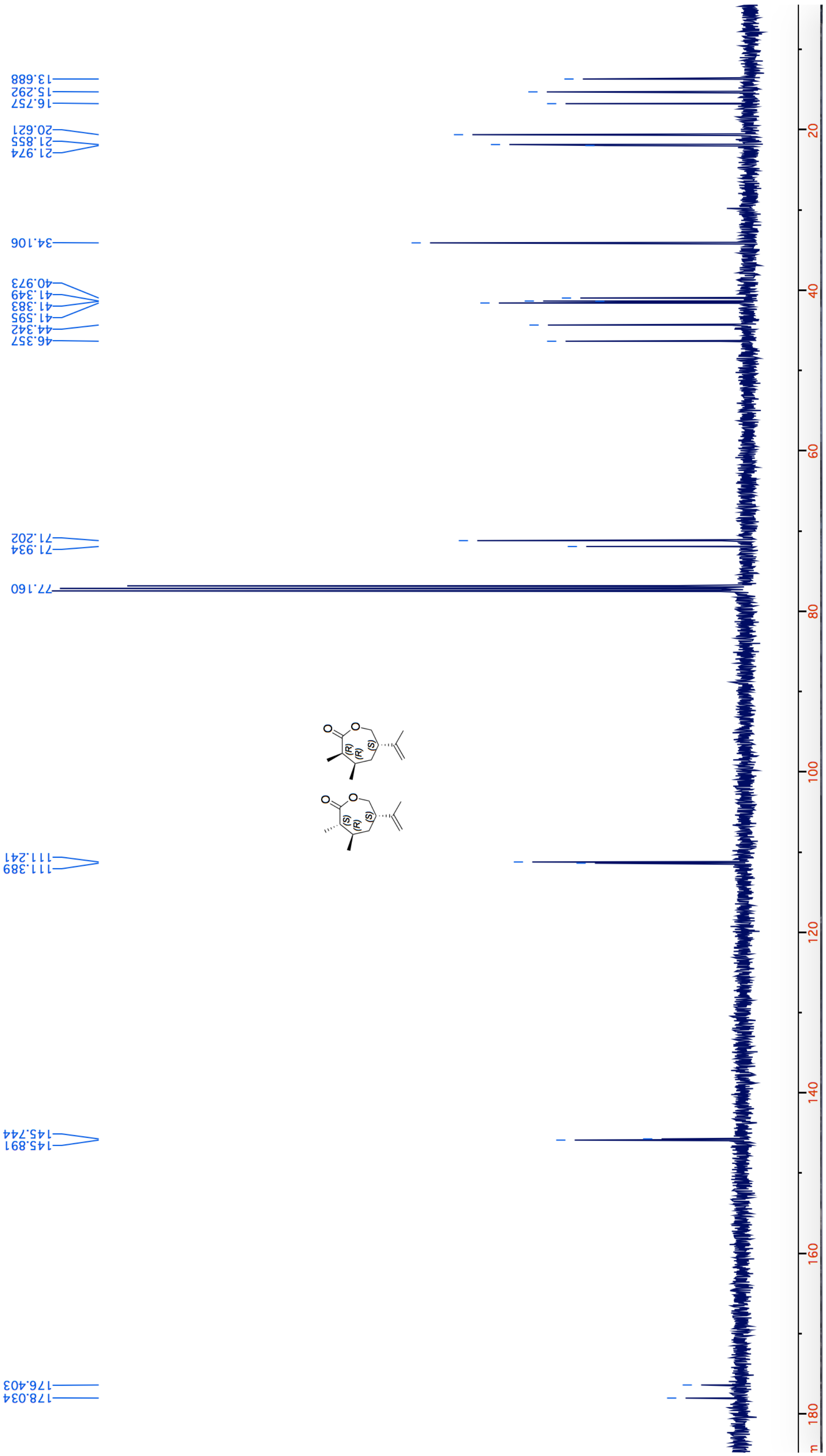
13.688
15.292
16.757
20.621
21.855
21.974
34.106
40.973
41.349
41.383
41.595
44.342
46.357
71.202
71.934
77.160
111.241
111.389
145.744
145.891
176.403
178.034
20
40
60
80
100
120
140
160
180

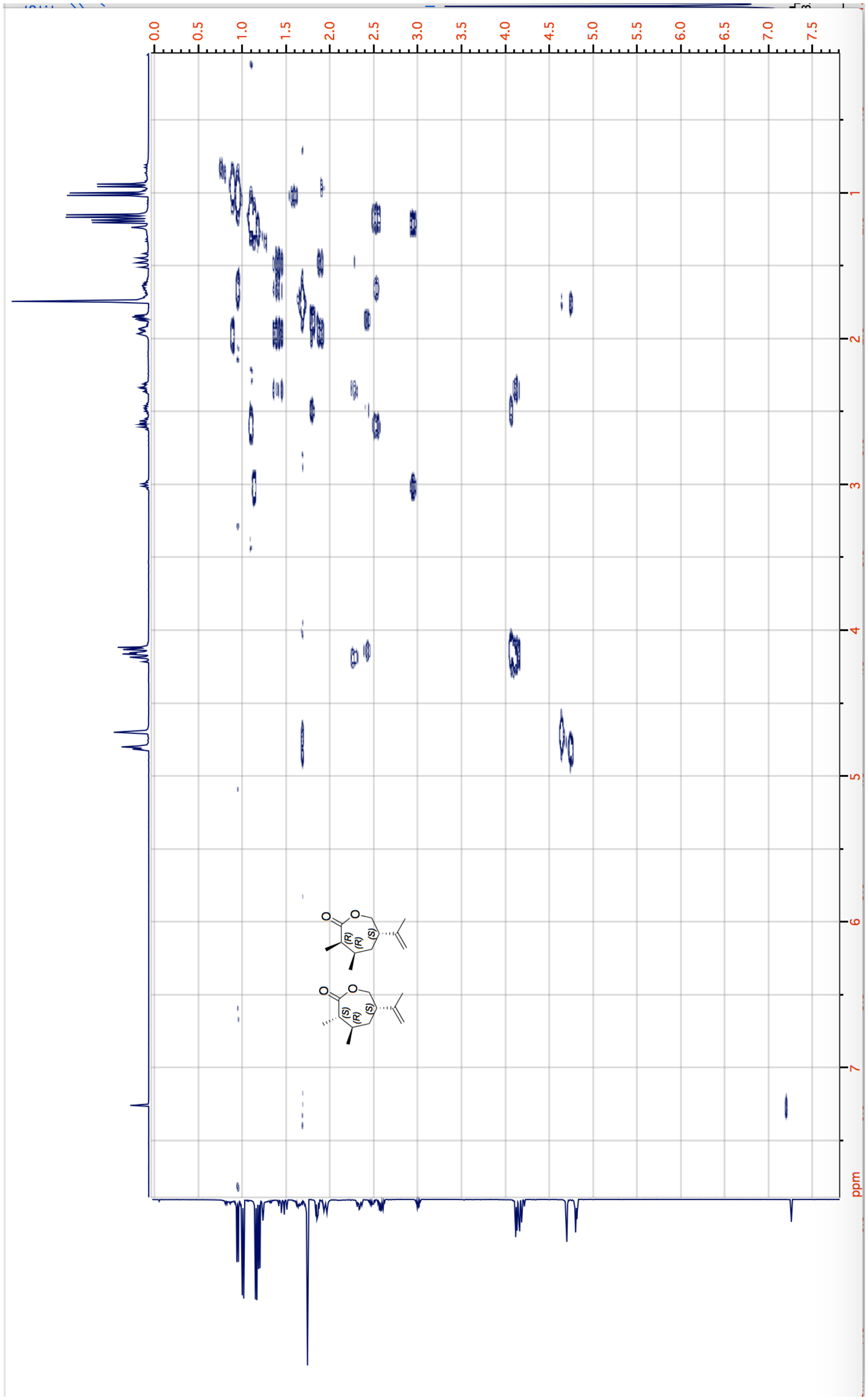
ppm

(5*R*,6*R*)-6-Hydroxy-2-methyl-5-(prop-1-en-2-yl)cyclohex-2-en-1-one, 7

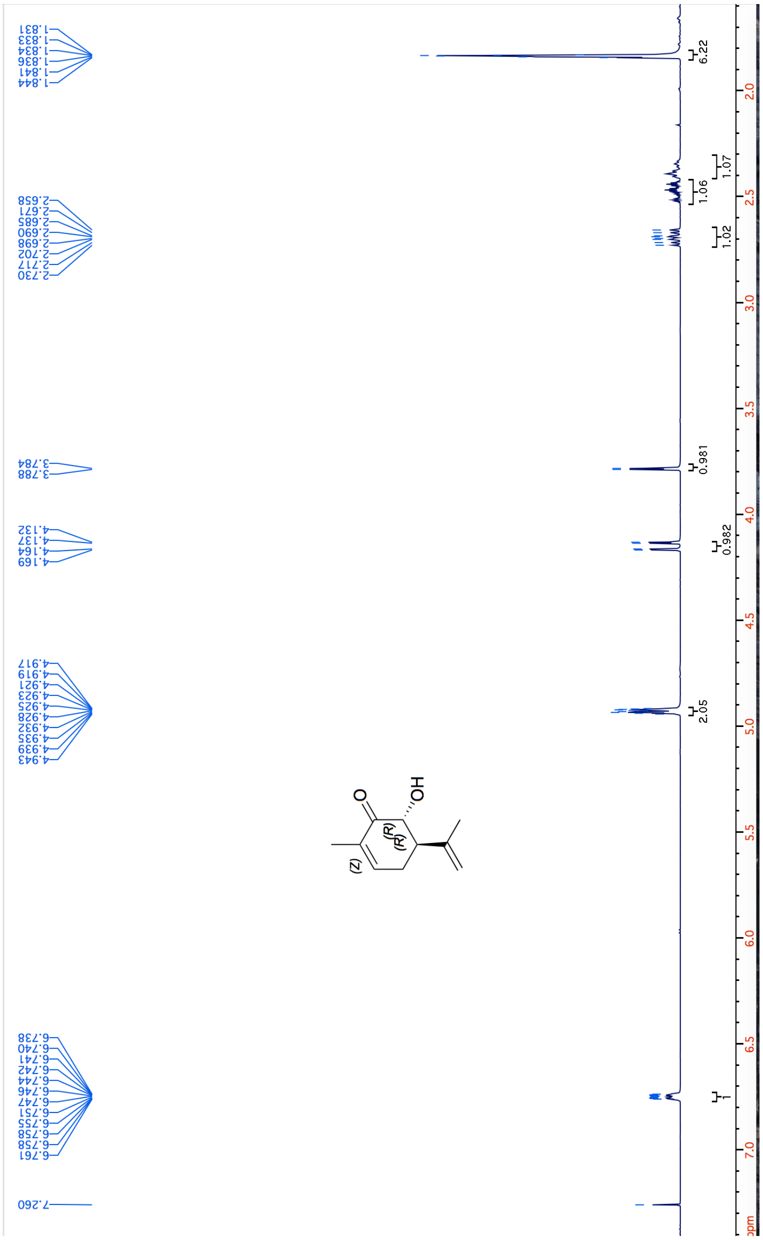

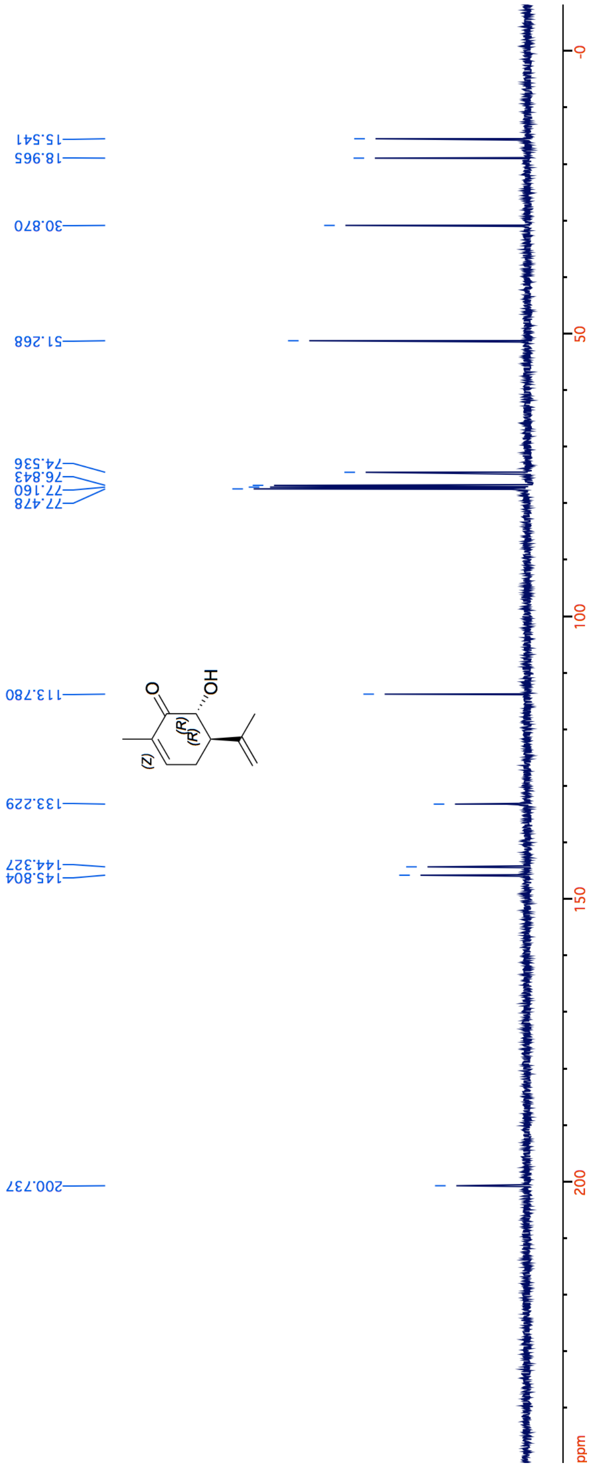
15.541
18.965
30.870
51.268
74.536
76.843
77.160
77.478
113.780
133.229
144.327
145.804
200.737
O
OH
(R)
(R)
(Z)
0
50
100
150
200
ppm

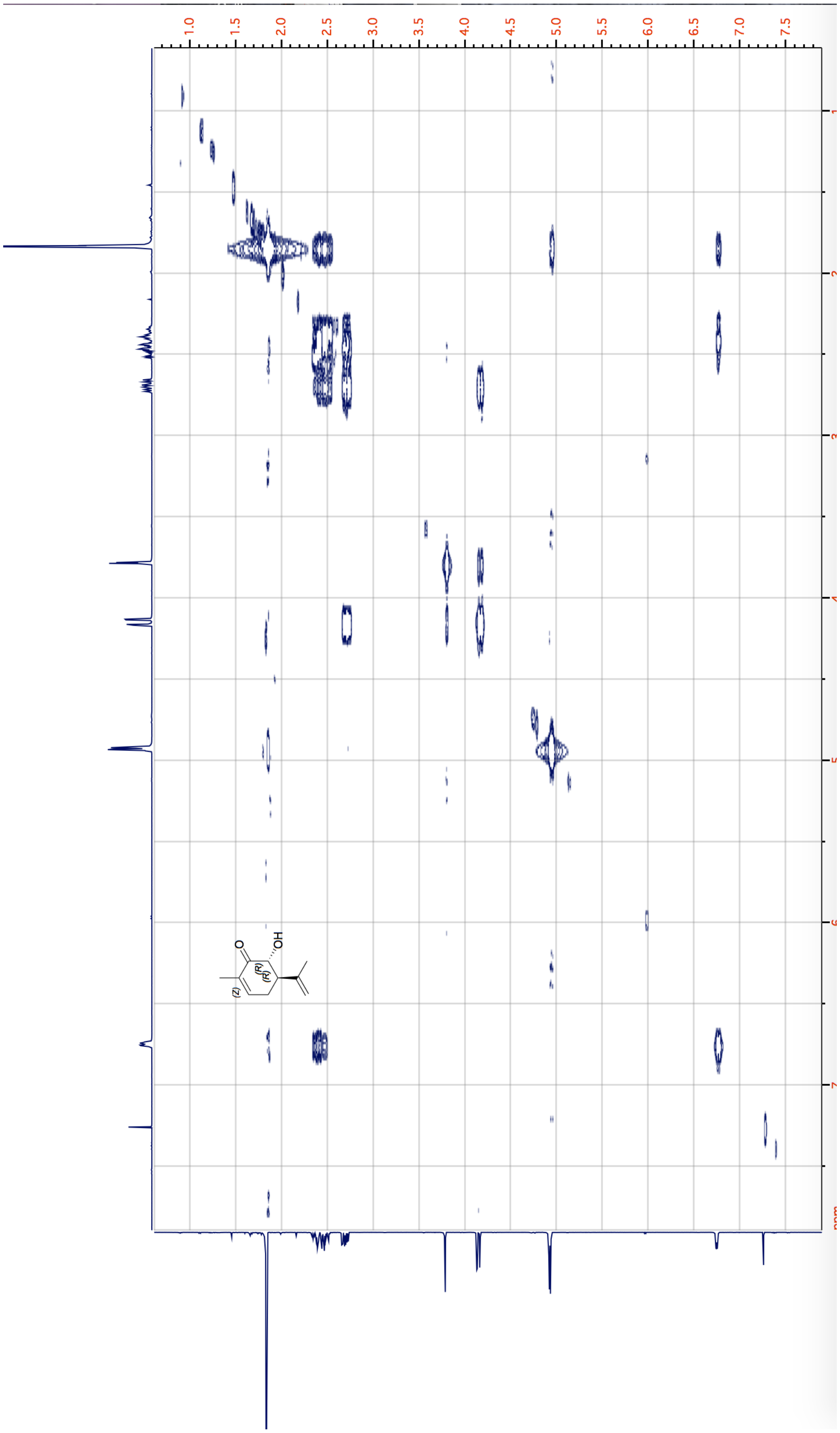

Supporting Information

(5*R*,6*S*)-6-Hydroxy-2-methyl-5-(prop-1-en-2-yl)cyclohex-2-en-1-one, 8

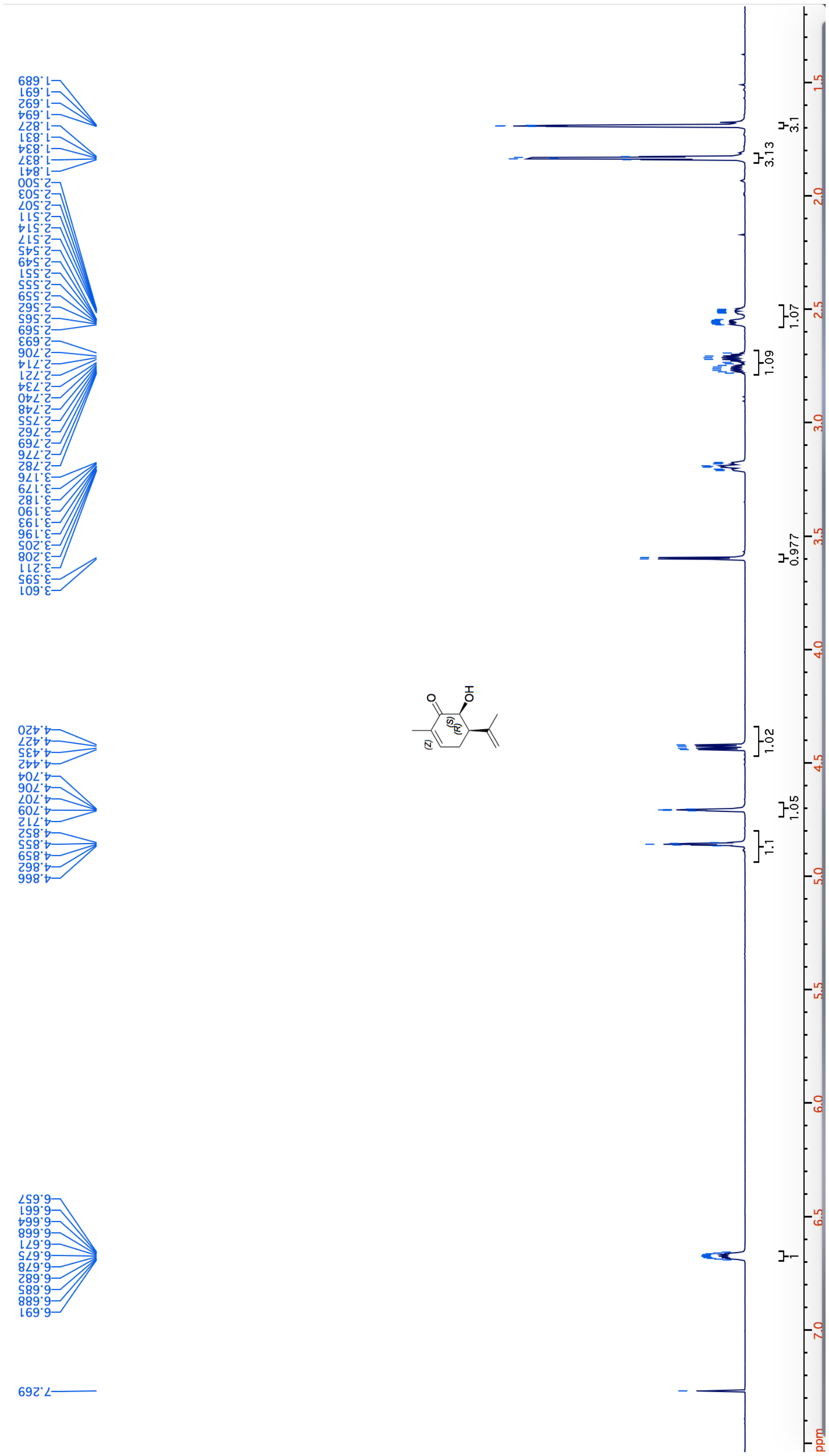

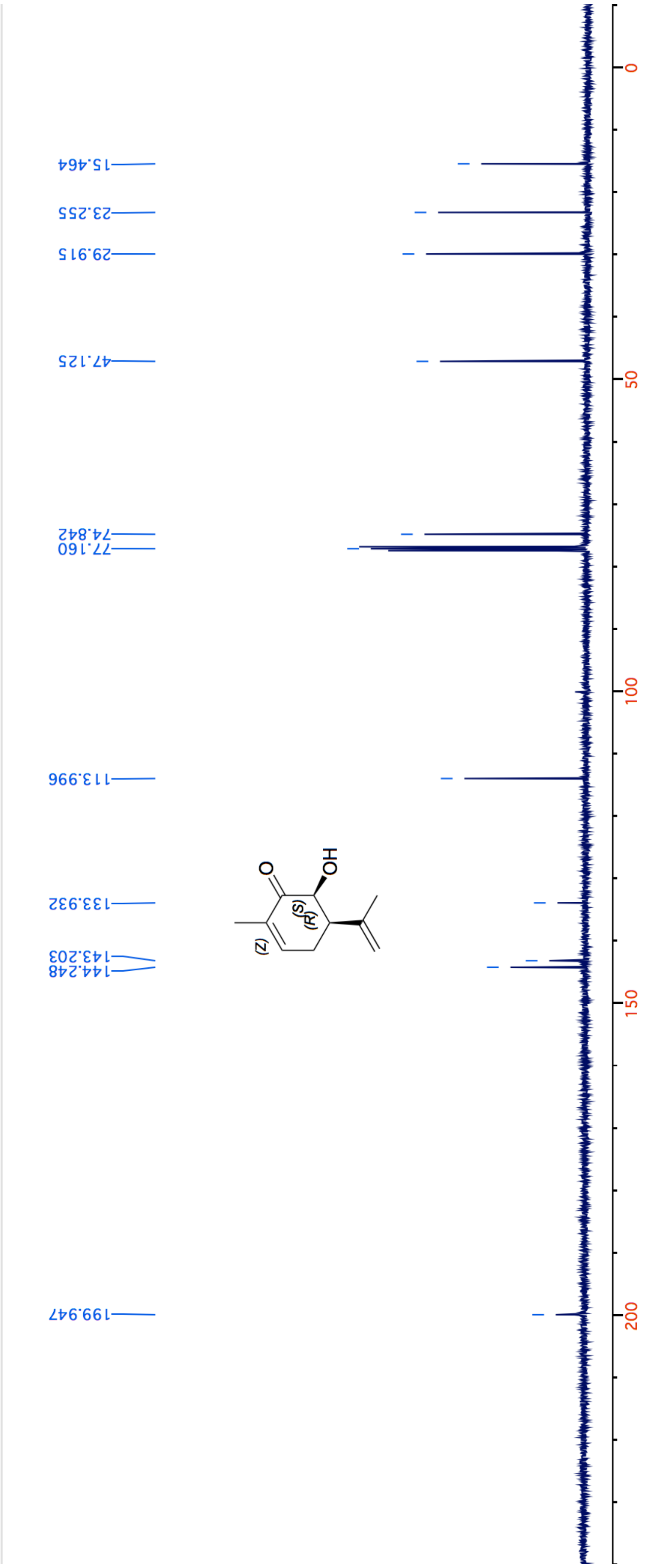
15.464
23.255
29.915
47.125
74.842
77.160
113.996
133.932
143.203
144.248
199.947
0
50
100
150
200
O
OH
(S)
(R)
(Z)

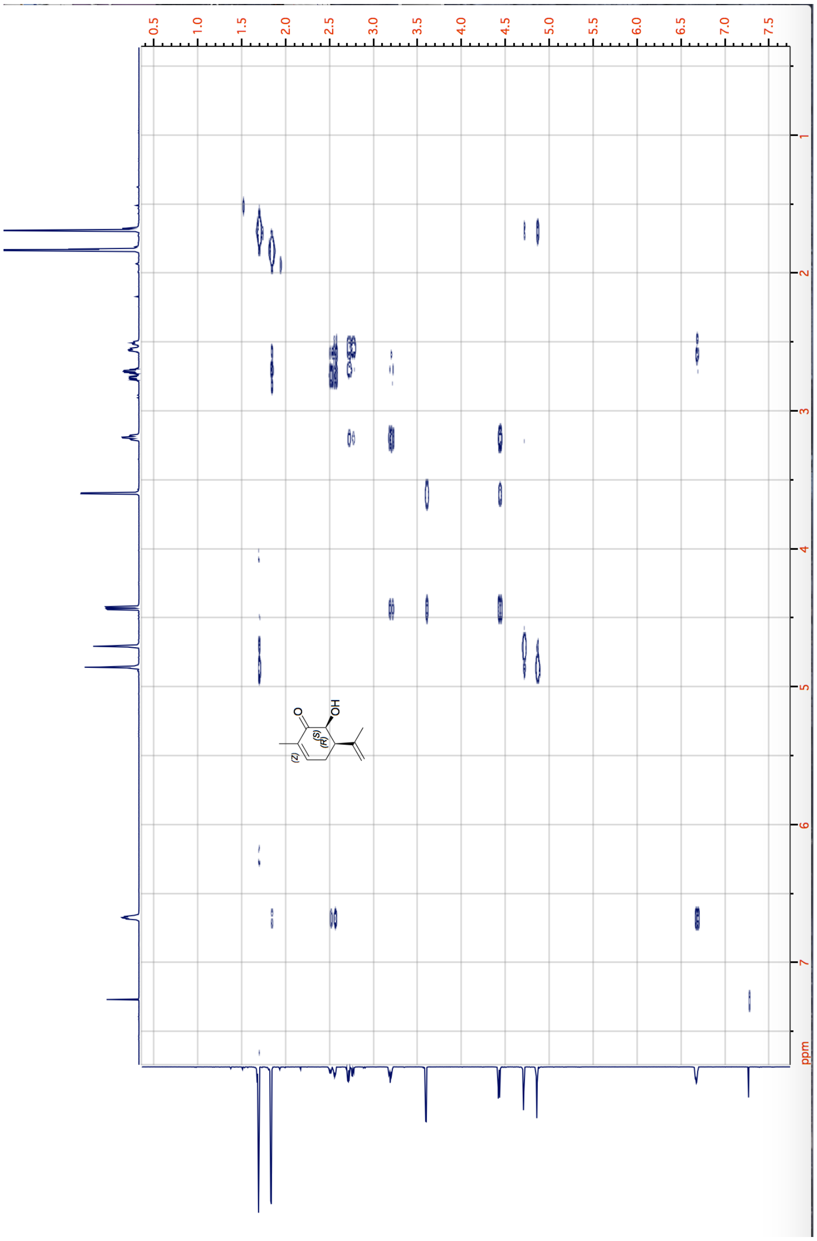

3. Enzymology

3.1 General procedure: Biocatalysis

Biohydrogenation substrates were prepared as stock solutions (250 mM) in absolute ethanol. Glucose dehydrogenase from *Pseudomonas sp.* (GDH), glucose-6-phosphate dehydrogenase from *Leuconostoc mesenteroids* (G-6-PDH), glucose-6-phosphate and potassium phosphate buffers KH_2PO_4 and K_2HPO_4 were obtained from Sigma-Aldrich. The oxidized and reduced forms of nicotinamide adenine dinucleotide phosphate ($NADP^+$/NADPH), and nicotinamide adenine dinucleotide (NAD^+/NADH) were obtained from Melford. Ketoreductase-to-go plates. The results were examined with achiral DB-Wax or GC and chiral Varianchiralsil-DEX CB column.

Table S1 Temperature methods utilised to determine conversion and yield% of substrates

Temperature Methods	Method 1 (DB-Wax column)	Method 3 Varianchiralsil -DEX CB column
Split injector ratio (1:x)	20: 1	splitless
Flow rate (mL.min^{-1})	1.5	1.0
Injector Temp (°C)	220	200
Start Temp (°C)	40	70
Start Temp Hold (min)	2	2
End Temp (°C)	210	200
End Temp Hold (min)	3	3
Temp ramping (°C/ min)	5	2
Detector Temp (°C)	250	200

Purified enzyme stocks of *pentaerythritol tetranitrate* reductase (PETNR) from *Enterobacter cloacae*[7] and Old Yellow Enzyme 3 (OYE3) from *Saccharomyces cerevisiae*[8] were employed. Additionally, the plasmid encoding the gene sequence of Old Yellow Enzyme 2 (OYE2) from *Saccharomyces cerevisiae*[7] was used for re-expression of OYE2. Details of the plasmid-gene constructs and *Escherichia coli* expression strains of the three OYEs are found in Table S1. The

protein sequences for OYE2 (UniProt: Q03558), OYE3 (UniProt: P41816) and PETNR (UniProt: P71278) were obtained from the UniProt online database (http://www.uniprot.org).

Table S2 Details of the three OYE gene constructs and expression strains

Enzyme	Source	Plasmid	Resr. Sites	*E.coli* Strain	His_6-tag	Ref.
OYE2	*S. cerevisiae*	pET21b	NdeI/NotI	BL21(DE3)	C-term	171
OYE3	*S. cerevisiae*	pET21a	NdeI/XhoI	BL21(DE3)	C-term	171
PETNR	*E. cloacae*	pET21a	NdeI/XhoI	BL21(DE3) Star	C-term	170

Resr. Sites = restriction sites through which the gene was cloned into the plasmid.

Cyclohexanone monooxygenase (CHMO_Phi1) from *Rhodococcus sp. Phi1*. UniProt amino acid sequence database ID = Q84H73.[9]

3.2 Expression and purification of recombinant OYE2

Expression and purification of OYE2 was performed using the method detailed below, based on the production and purification of OYE2 as described previously.[8]

3.2.1 Medium and sterile reagents preparation

Sterile stock solutions (1000x) of ampicillin (100 mgmL^{-1}) and isopropyl β-D-1-thiogalactopyranoside (IPTG; 0.5 M) in distilled water were filter sterilised (0.2 mm filtration) and stored at −20 °C prior to use. Super optimal broth (SOC) medium was prepared by combining tryptone (20 gL^{-1}), yeast extract (5 gL^{-1}), NaCl (10 mM), KCl (2.5 mM), $MgCl_2$ (10 mM) and $MgSO_4$ (10 mM) in distilled water and sterilising by autoclaving. A filter-sterilised stock of glucose in distilled water was prepared (1M; 0.2 mm filtration), and added aseptically to the sterile medium to a final concentration of 20 mM. Lysogeny broth-Miller (LB) was prepared by combining tryptone (10 gL^{-1}), yeast extract (5 gL^{-1}) and NaCl (10 gL^{-1}) followed by autoclaving. Lysogeny broth-Miller agar (LB agar) was prepared as for LB, except agarose (15 gL^{-1}) was added to the medium prior to autoclaving. After sterilisation, the medium was cooled to ~ 50 °C, and a sterile ampicillin solution was added (100 mgmL^{-1}) before pouring into sterile agar plates.

3.2.2 Transformation of competent cells

Purified OYE2 plasmid DNA (0.4 mL; Table S2) was mixed with pre-chilled competent *E. coli* BL21 (DE3) cells (50 mL) and incubated on ice for 30 minutes. The suspension was heat shocked by incubating at 42 °C for 30 seconds, followed by cooling on ice for 2 minutes. Cell recovery and growth was initiated by the addition of SOC medium (0.45 mL) and the culture was incubated at 37 °C for 1 hour at 200 rpm agitation. The cell suspension was spread aseptically on to LB agar, containing ampicillin (100 $mgmL^{-1}$), and incubated overnight at 37 °C.

3.2.4 Growth and expression of OYE2 clone

Starter cultures of OYE2 in *E. coli* BL21(DE3) were produced by inoculating LB media (5 mL) containing ampicillin (100 $mgmL^{-1}$) with a colony from the transformation plate (Section 3.2). Cultures were incubated for 1 hour at 37 °C with 200 rpm agitation. Aliquots of starter cultures (0.5 mL) were used to inoculate (6 x 1L LB) containing ampicillin (100 $mgmL^{-1}$) and incubated at 37 °C with 200 rpm agitation until the OD 600 nm reached ~ 0.5. Protein expression was initiated by the addition of sterile IPTG (0.5 mM) and the cultures were incubated overnight at 37 °C with 200 rpm agitation. Cultures were harvested by centrifugation (6,238 *g*, 5 °C, 10 minutes), and the cell pellet was frozen in liquid nitrogen and stored at −80 °C.

3.2.5 Purification of OYE2

The OYE2 cell pellet was resuspended in lysis buffer (50 mM KH_2PO_4/K_2HPO_4 pH 8.0 containing 10 $mgmL^{-1}$ DNAse and 1 x Complete EDTA-free protease inhibitor blend). Lysozyme (10 $mgmL^{-1}$) and flavin mononucleotide (FMN) were added, and the slurry was stirred until uniform. The cells were disrupted by sonication (BANDELIN SONOPULS) (20 minutes) and the cell debris was pelleted by centrifugation (4,3667 *g*, 1 hour, 4 °C). The supernatant was retained, and NaCl (3.9 mL, 0.3 M) and imidazole (0.65 mL, 10 M) were added.

The OYE2 supernatant was applied to a Ni-NTA affinity column (30 mL) pre-equilibrated in wash buffer 1 (50 mM KH_2PO_4/K_2HPO_4 pH 8.0 containing 10 mM imidazole). The column was washed with wash buffer 2 (50 mM KH_2PO_4/K_2HPO_4 pH 8.0 containing 300

mM NaCl and 25 mM imidazole). Protein was recovered from the column in a step to elution buffer (50 mM KH_2PO_4/K_2HPO_4 pH 8.0 containing 300 mM NaCl and 200 mM imidazole).

Eluted protein fractions were checked for protein content and purity by SDS-PAGE using stain-free precast gels (Gel DOC EZ Imager BioRad) in standard running buffer (25 mM Tris containing 192 mM glycine and 0.1 % sodium dodecyl sulfate). Samples were prepared by combining protein fractions (5 mL) with water (5 mL) and 2 X SDS gel loading buffer (10 mL; 100 mM Tris pH 6.8 containing 4 % SDS, 0.2 % bromophenol blue, 200 mM 2-mercaptoethanol and 20 % glycerol). The samples were boiled at 95 °C for 5 min to denature the proteins, and centrifuged at 4 °C for 1 minute (13,000 *g*) to pellet any debris. Samples were loaded onto the precast gel, and run for 20 min at constant voltage of 180 V, and scanned using transilluminator.

The purest protein fractions (Figure S1) were dialysed against a storage buffer (10 mM Tris pH 7.0; 7 L) to remove the NaCl and imidazole prior to protein storage. The dialysis was performed overnight in the cold room (4 °C). The final protein concentration was determined by measuring the absorbance at 280 nm, and using the extinction coefficient of 10600 M^{-1} cm^{-1}.

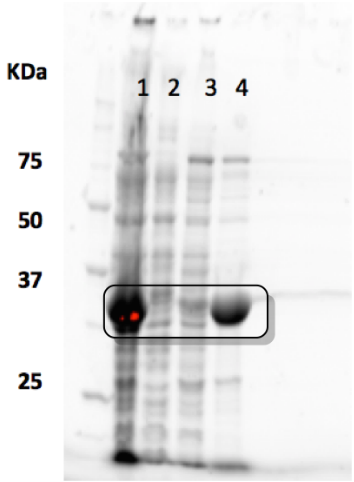

Figure S1: SDS-PAGE of the first OYE2 affinity chromatography purification with Ni^{2+} NTA Lanes 1-4 = elution fractions for first column and black box shows OYE2 bands

3.3 General enzymatic procedure for reduction by PETNR, OYE2 and OYE3

The standard enzymatic reaction (1 mL) was achieved in buffer solution of KH_2PO_4/ K_2HPO_4 (50 mM, pH 7.0), comprises [substrate] = 5 mM, [$NADP^+$] = 15 μM, GDH or G-6-PDH = 10 U, glucose or glucose-6-phosphate 15 mM, [Enzyme] = 2 or 10 μM. The reaction mixture was shaken at required temperature 30 °C at 135 rpm for desired time. The reaction was then terminated by extraction into an organic layer of 800 μL of EtOAc: internal standard (+)-limonene (99.5:0.5). The organic mixture was dried over $MgSO_4$, and analysed with GC using DB-wax column to determine % of yield, conversion and diastereomeric excess.

3.3.1 Biocatalytic reduction of *R*-(−)-carvone 1

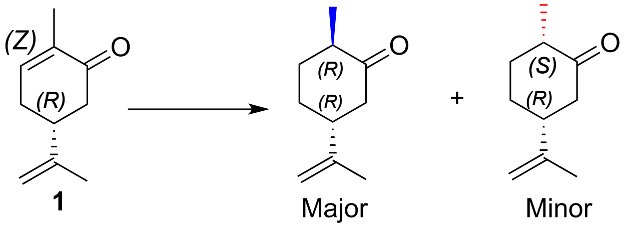

Table S3 The effect of temperature and co-factor on bioreduction of **1** by OYE2

	T (22 °C)		T (30 °C)		T (37 °C)	
**Co-factor**	**Yield%**	**d.e.%**	**Yield%**	**d.e.%**	**Yield%**	**d.e.%**
$NADP^+$/ GDH	80	86	82	85	50	86
$NADP^+$/ G-6-DH	73	85	58	79	45	93
NAD^+/ GDH	82	85	85	85	46	95

1) Conditions: Enzyme (2 μM), Substrate (5 mM) pH 7.0 0f 50 mM KP buffer solution, Co-factor (15 μM), GDH or G-6-DH (10 U), 130 rpm for 24 h. 2). No products were determined using co-factors NADPH or NADH.

Table S4: The effect of time on bioconversion of **1** via OYE2 with respect to the yield and diastereomeric excess.

Time (h)	Yield%	d.e%
1	83	95
2	84	93
4	79	90
6	77	87
24	79	80

1) Conditions: Enzyme (2 μM), Substrate (5 mM) pH 7.0 0f 50 mM KP buffer solution, $NADP^+$ (15 μM), GDH (10 U), Glucose (15 μM), 30 °C at 130 rpm.

Table S5 Biotransformation of **1** via OYE3 enzyme*

	24 h		2h	
**Enzyme**	**Yield%**	**d.e.%**	**Yield%**	**d.e.%**
OYE3	58	93	80	94

*Conditions: Enzyme (10 μM), pH 7.0 0f 50 mM KP buffer solution, $NADP^+$ (15 μM), GDH (10 U), Glucose (15 μM), 30 °C at 130 rpm

Table S6 The influence of enzyme and $NADP^+$ concentrations on bioreduction of **1** using OYE2

Entry	[Enzyme] μM	[$NADP^+$] μM	Yield%	d.e.%
**1**	2	15	79	80
**2**	2	25	57	93
**3**	2	50	57	93
**4**	2	100	54	93
**5**	10	15	82	85
**6**	10	25	65	66
**7**	10	50	65	69
**8**	10	100	55	64

1) Conditions: Substrate (5 μM) pH 7.0 0f 50 mM KP buffer solution, 30 °C at 130, 24 h**.**

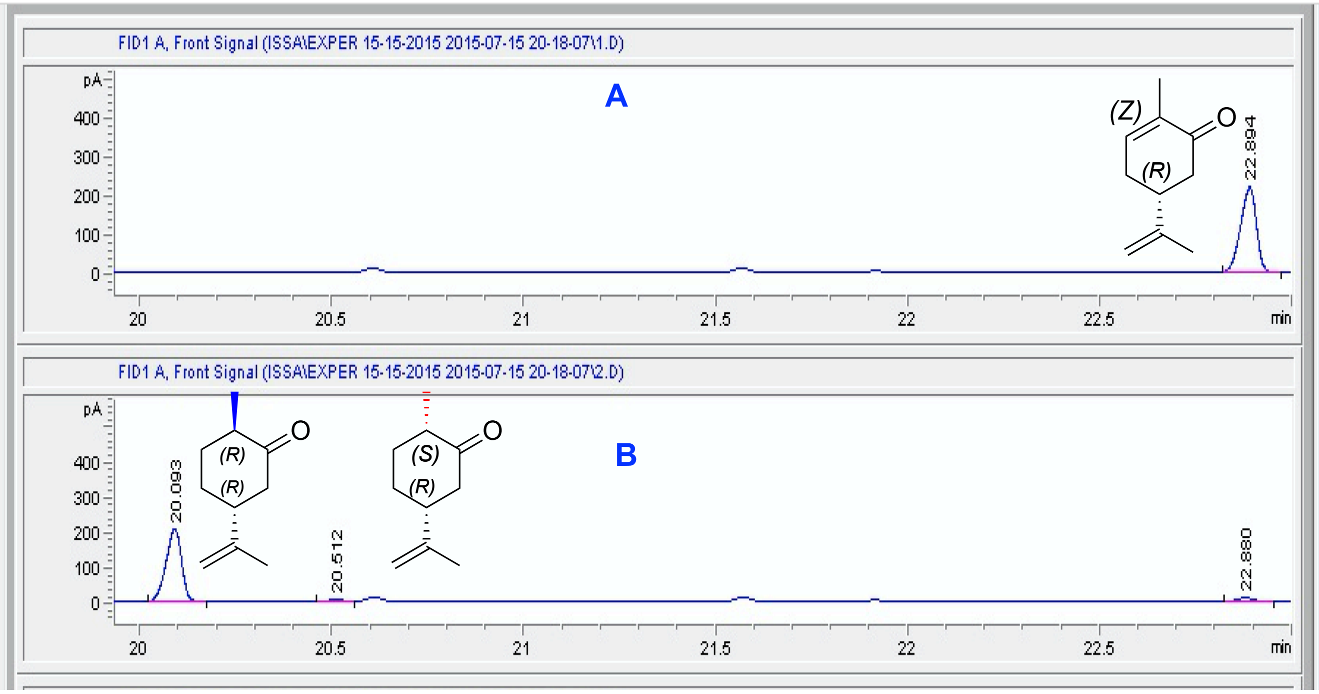

Figure S2: GC chromatographs of A) Control solution of R-(−)-carvone B) Bio-catalytic reaction mixture using OYE2. Bio-reduction conditions: Enzyme (10 μM), Substrate (5 mM) pH 7.0 0f 50 mM KP buffer solution, $NADP^+$ (15 μM), GDH (10 U), Glucose (15 μM), 30 °C at 130 rpm for 24 h.

3.3.2 Biocatalytic reduction of 6-methylcarvone diastereomers mixture

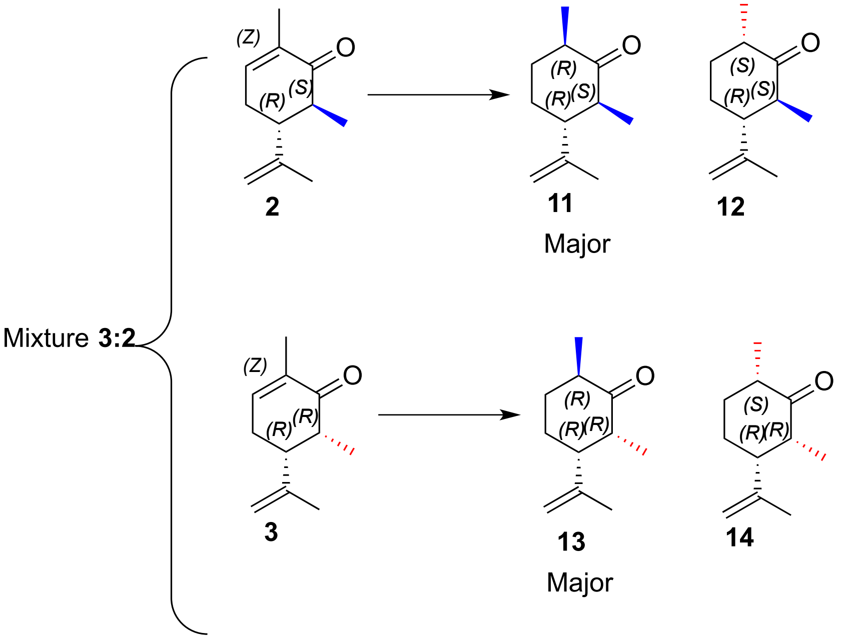

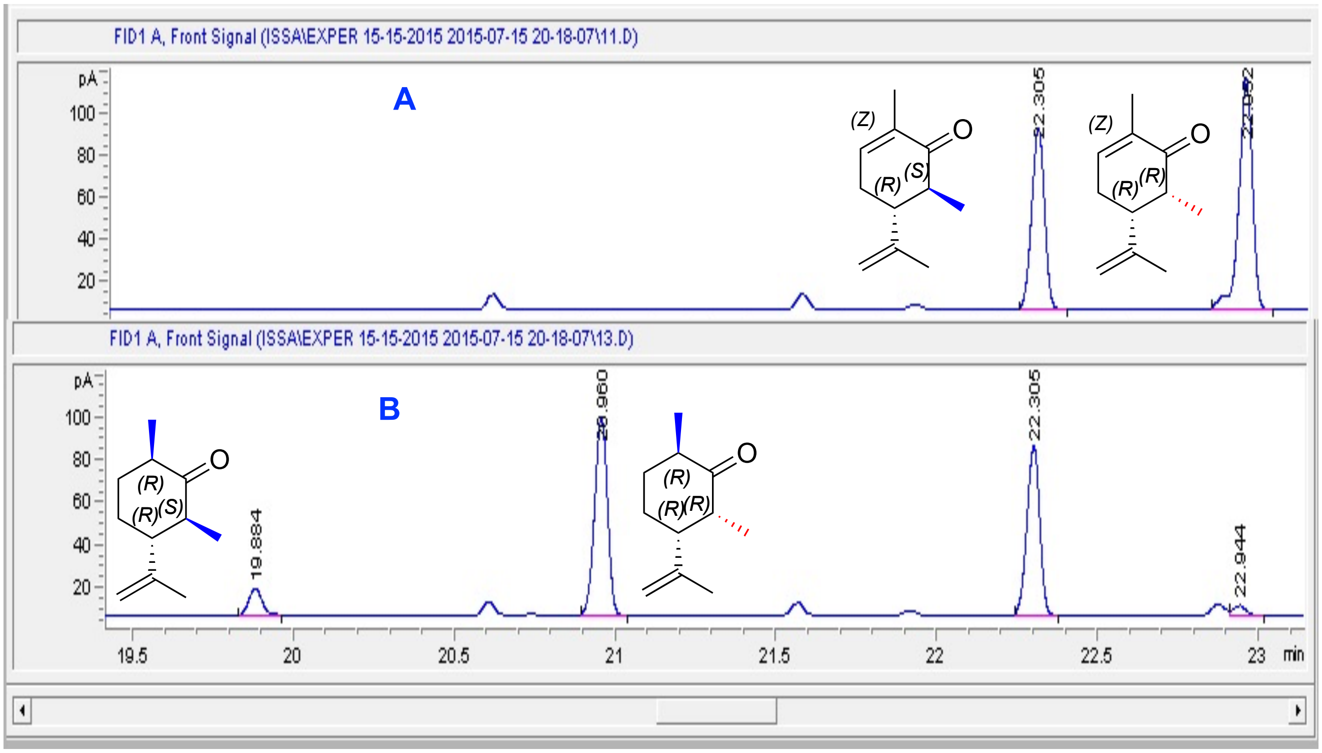

Figure S3: GC chromatographs of A) Control solution of 6-methycarvone diastereomers mixture B) Bio-catalytic reaction mixture using PETNR. Bio-reduction conditions: Enzyme (10 µM), Substrate (5 mM) pH 7.0 0f 50 mM KP buffer solution, $NADP^+$ (15 µM), GDH (10 U), Glucose (15 mM), 30 °C at 130 rpm for 24 h.

3.3.2.1 Biocatalytic reduction of 6-*S*-methylcarvone diastereomer

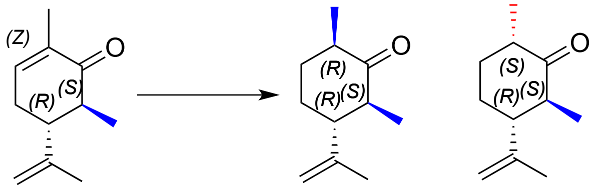

2 11 Major 12

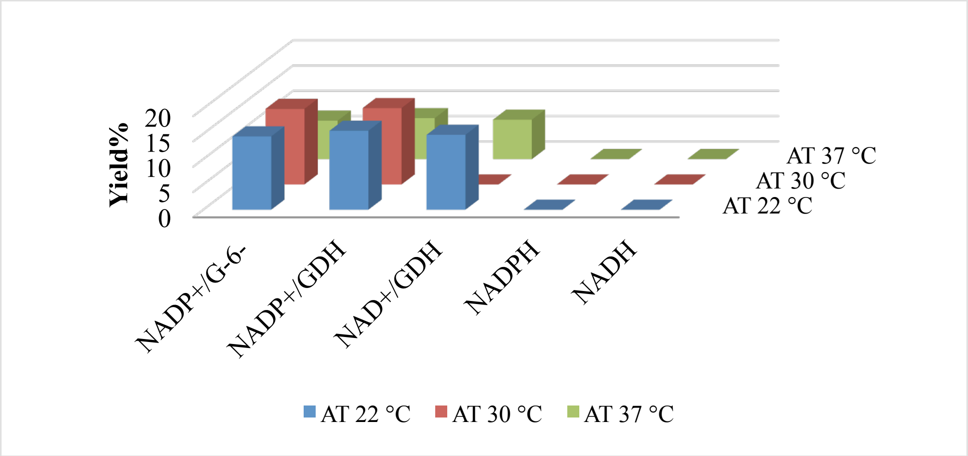

Figure S4: The effect of temperature and co-factor on bioreduction of **2** by OYE2. Reaction conditions: Enzyme (2 μM), Substrate (5 mM) pH 7.0 0f 50 mM KP buffer solution, $NADP^+$ (15 μM), GDH (10 U), Glucose (15 mM), at 130 rpm for 24 h.

Table S7: Bioconversion of **2** using OYE2 as a function of time (h). Bio-reduction conditions: Enzyme (2 μM), Substrate (5 mM) pH 7.0 0f 50 mM KP buffer solution, $NADP^+$ (15 μM), GDH (10 U), Glucose (15 mM), 30 °C at 130 rpm for 24 h.

Time	Yield%	d.e.%
**1**	12	≥99
**2**	17	≥99
**4**	15	≥99
**6**	15	≥99
**24**	15	≥99

Table S8. Bioconversion optimisation of **2** using OYE2 enzyme and $NADP^+$ concentration parameters Bio-reduction conditions: Substrate (5 mM) pH 7.0 0f 50 mM KP buffer solution, 30 °C at 130 rpm for 24 h.

[Enzyme]	[$NADP^+$]	Yield%	d.e.%
2	15	15	≥99
2	25	8	≥99
2	50	7	≥99
2	100	6	≥99
10	15	17	≥99
10	25	17	≥99

10	50	13	≥99
10	100	13	≥99

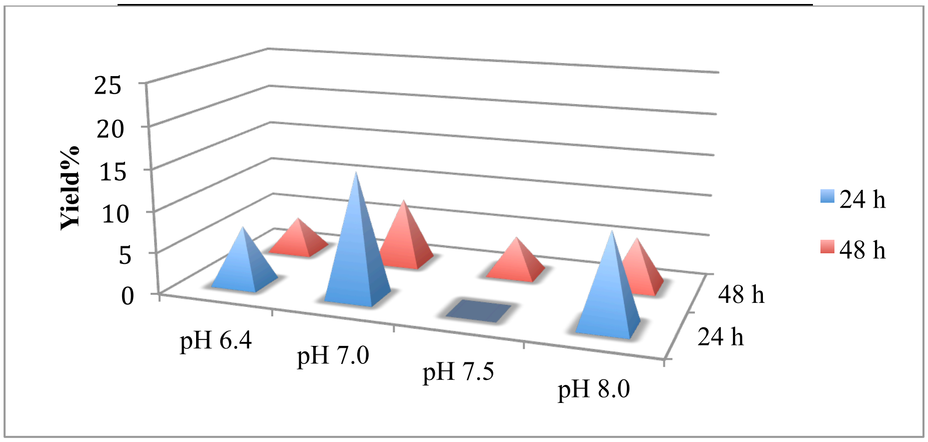

Figure S5. The effect of pH buffer solution and time parameter on biotransformation of **2** via OYE2 enzyme. Reaction conditions: Substrate (5 mM), $NADP^+$ (15 µM), GDH (10 U), Glucose (15 mM), 30 °C at 130 rpm.

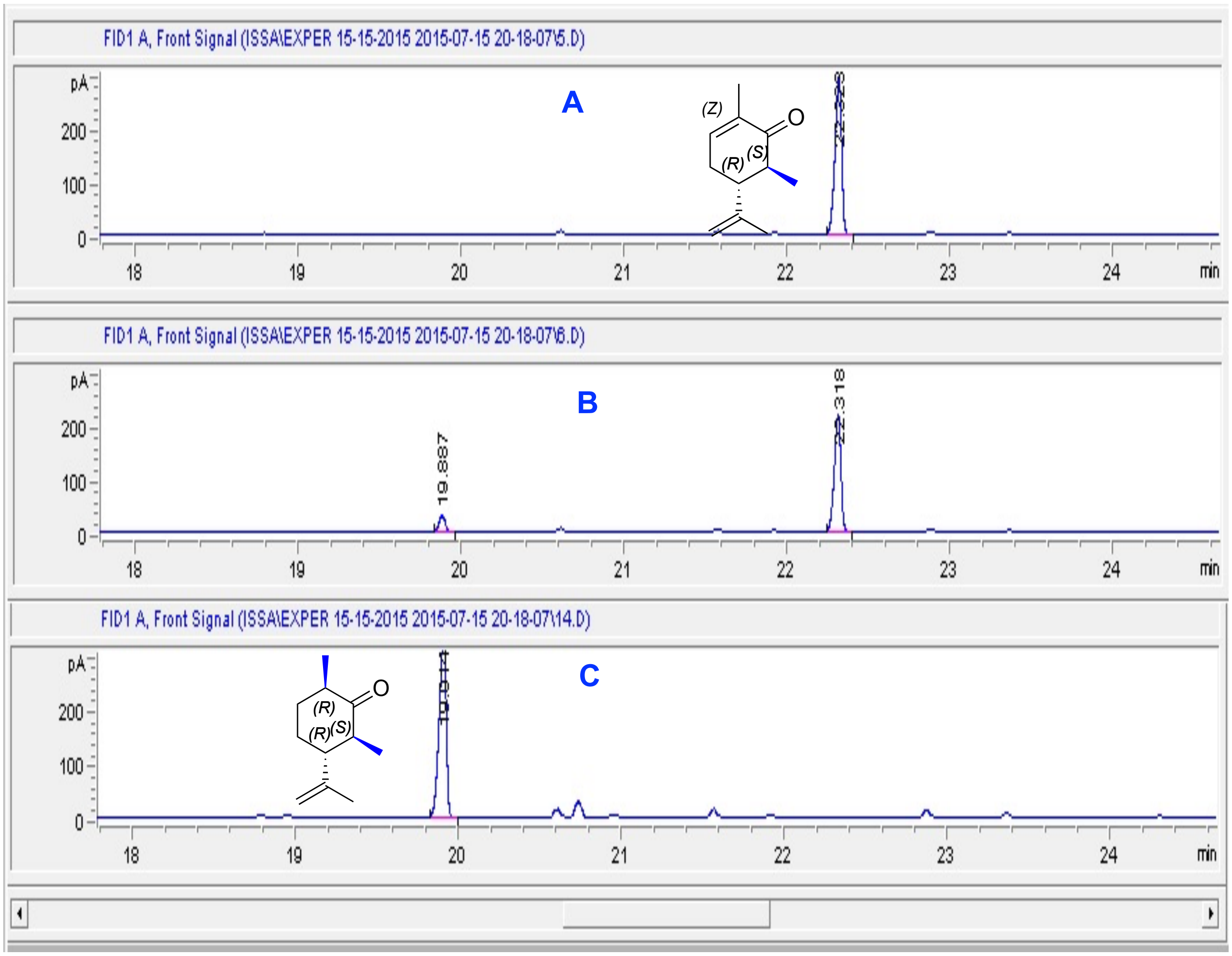

Figure S6. GC chromatographs of A) Control solution of 6-*S*-methylcarvone diastereomer **2** B) Bio-catalytic reaction mixture using OYE2 C) Standard reference of product. Bio-reduction conditions: Enzyme (2 µM), Substrate (5 mM) pH 7.0 0f 50 mM KP buffer solution, $NADP^+$ (15 µM), GDH (10 U), Glucose (15 mM), 30 °C at 130 rpm for 24 h.

3.3.2.2 Biocatalytic reduction of 6-*R*-methylcarvone diastereomer

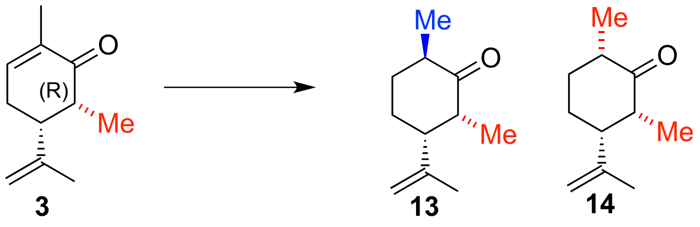

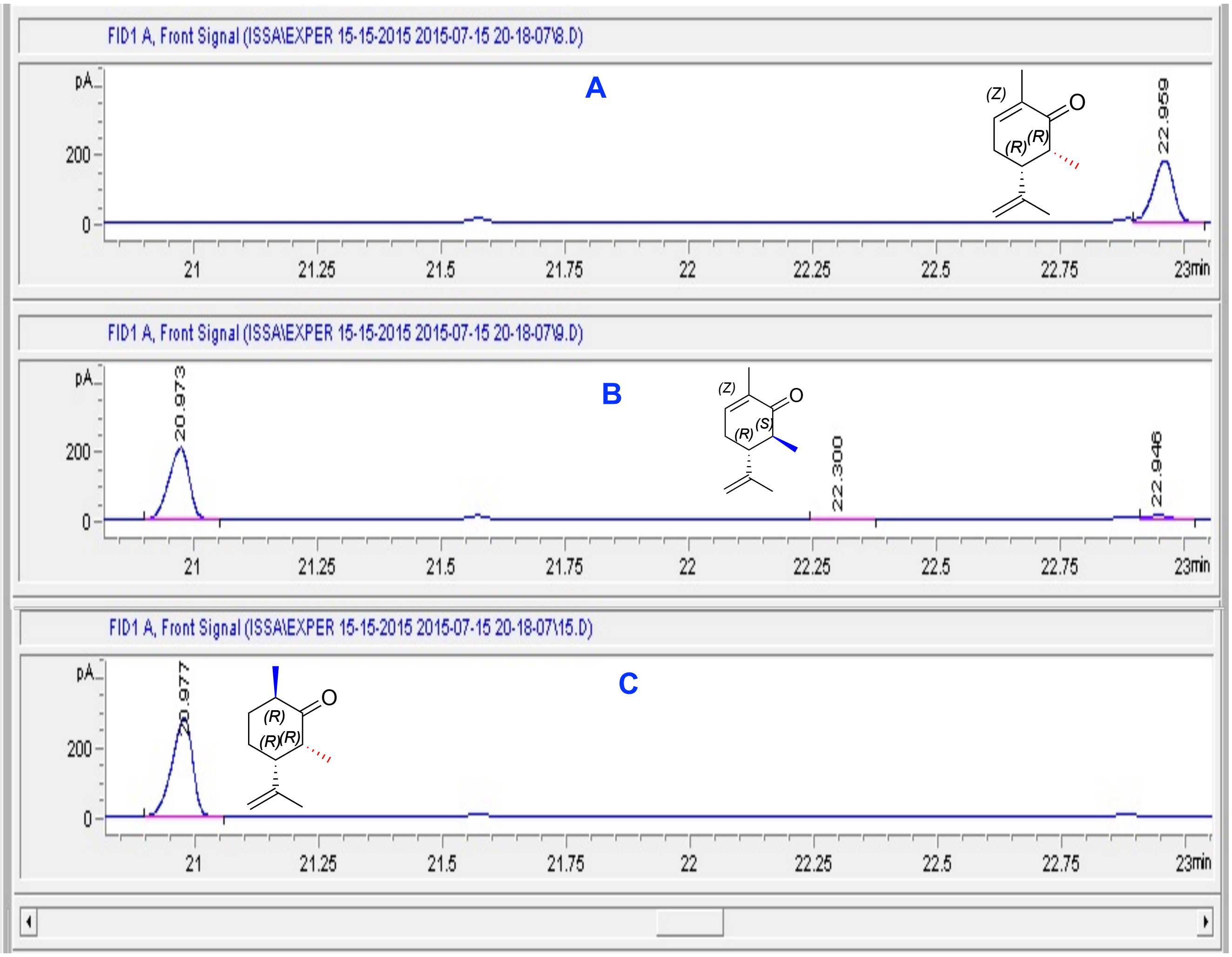

Figure S7: GC chromatographs of A) Control solution of 6-*R*-methycarvone diastereomer **3** B) Bio-catalytic reaction mixture using PETNR C) Standard reference of product. Bio-reduction conditions: Enzyme (10 µM), Substrate (5 mM) pH 7.0 of 50 mM KP buffer solution, $NADP^+$ (15 µM), GDH (10 U), Glucose (15 mM), 30 °C at 130 rpm for 2 h. A small amount of 6-*R*-methyl carvone **3** has epimerised to 6-*S*-methyl carvone **2**

3.3.3 Biocatalytic reduction of 6-hydroxycarvone diastereomers 4 and 5

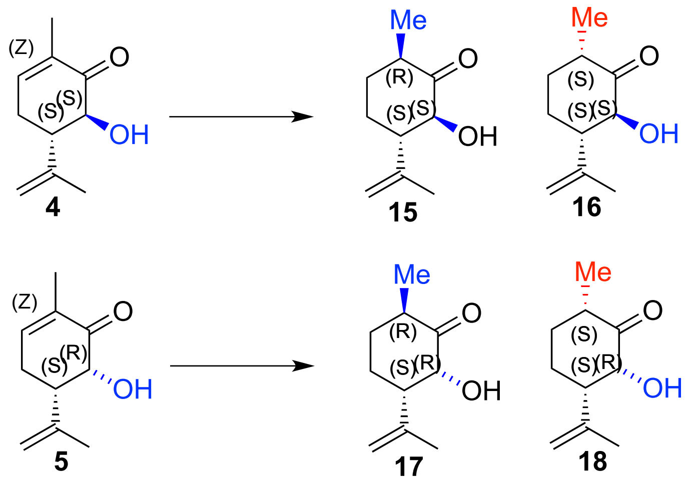

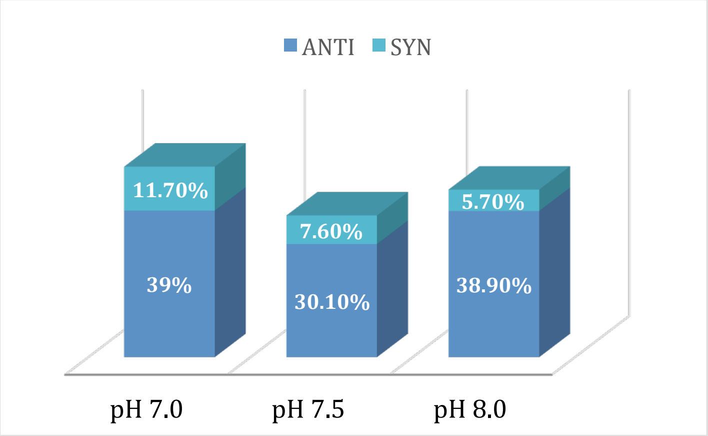

Figure S8. Optimisation pH buffer solution of KP of bioconversion of *anti* **4** and *syn* **5**. Where, 1 mL scale, substrate (5 mM), OYE2 (2 μM), $NADP^+$ (15 μM)/GDH (15 U), glucose (15 mM), pH 7.0 of KP buffer solution (50 mM), 24 h, 130 rpm. The figure showed the yield% of **15** and **17**.

Table S9. Optimisation of [NADP$^+$] and [OYE2] on outcomes of bioreduction of *anti* **4** and *syn* **5**. Where, 1 mL scale, substrate (5 mM), pH 7.0 of KP buffer solution (50 mM), 24 h, 130 rpm

Substrate	Product	[NADP$^+$] µM	[OYE2] µM	Yield %	d.e.%
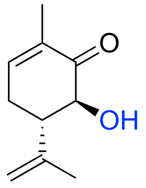	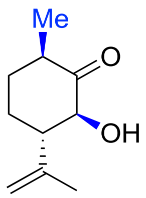	15	2	39	≥99
		25	2	30	≥99
		50	2	31	≥99
		100	2	34	≥99
		15	10	40	≥99
		25	10	49	≥99
		50	10	39	≥99
		100	10	42	≥99
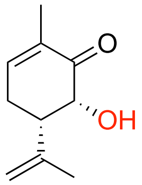	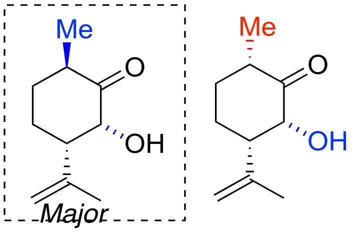	15	2	11	≥99
		25	2	8	≥99
		50	2	7	≥99
		100	2	6	≥99
		15	10	30	16
		25	10	26	16
		50	10	26	16
		100	10	12	16

Table S10. Bioconversion of *anti* **4** and *syn* **5** by OYE3 within 2 and 24 h. Reaction conditions: substrate (5 mM), OYE3 (10 μM), $NADP^+$ (15 μM)/GDH (15 U), glucose (15 mM), pH 7.0 of KP buffer solution (50 mM), 130 rpm.

**Substrate**		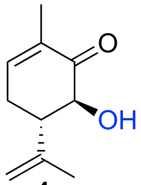 **4**		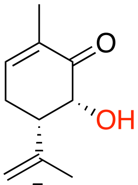 **5**	
**Product**		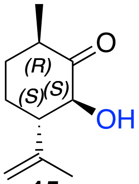 **15**		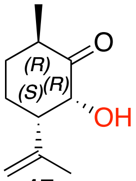 **17** 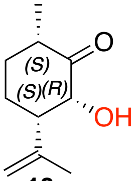 **18**	
**OYE3**	**Time (h)**	2	24	2	24
	**Yield %**	14	17	nd	nd
	**d.e.%**	≥99	≥99	nd	nd

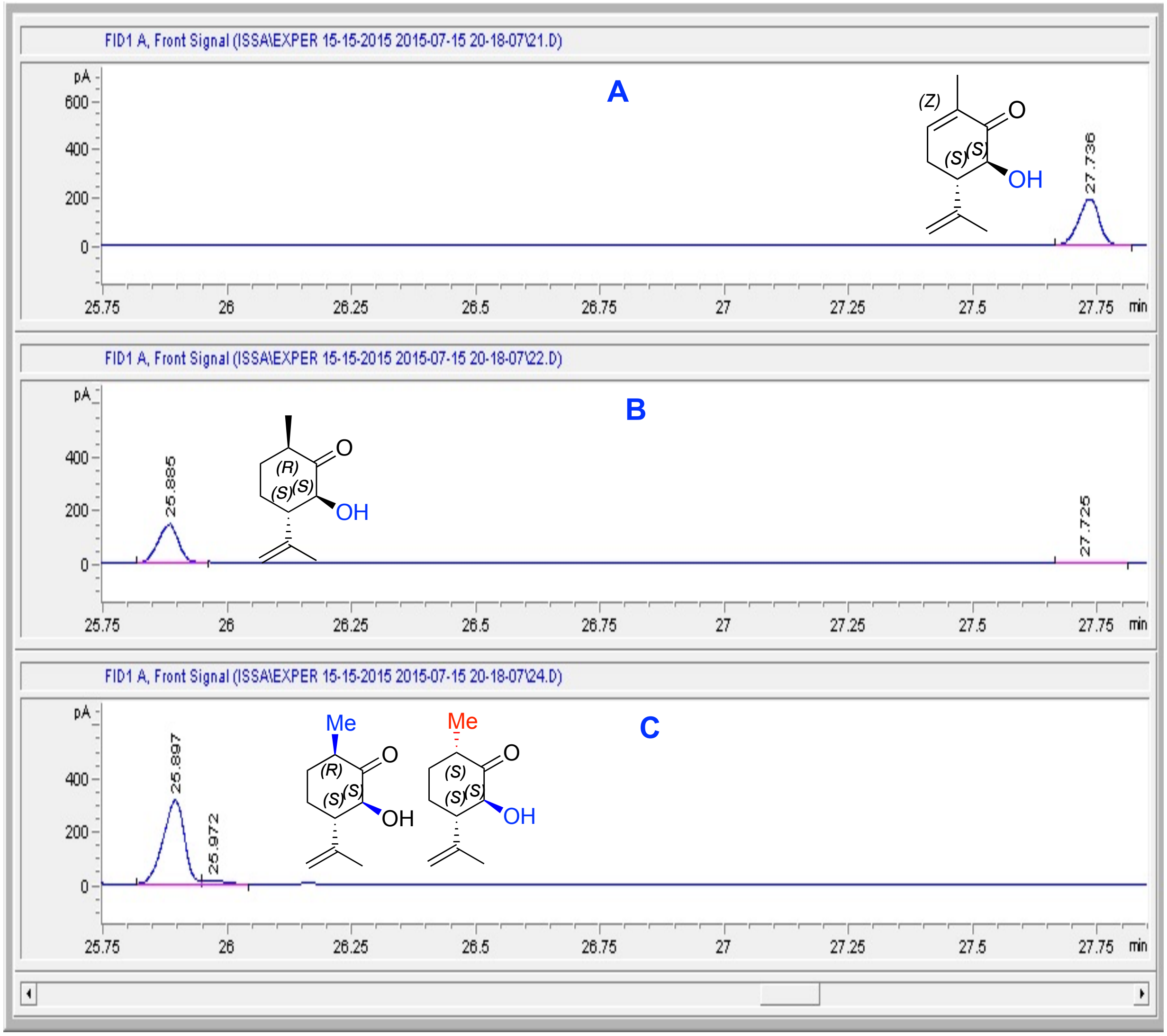

Figure S9. GC chromatographs of A) Control solution of 6-*S*-hydroxycarvone diastereomer (*anti*) **4** B) Bio-catalytic reaction mixture using PETNR C) Standard reference of diastereomeric mixture of the product. Bio-reduction conditions: PETNR (10 µM), Substrate (5 mM) pH 7.0 0f 50 mM KP buffer solution, $NADP^+$ (15 µM), GDH (10 U), Glucose (15 mM), 30 °C at 130 rpm for 24 h.

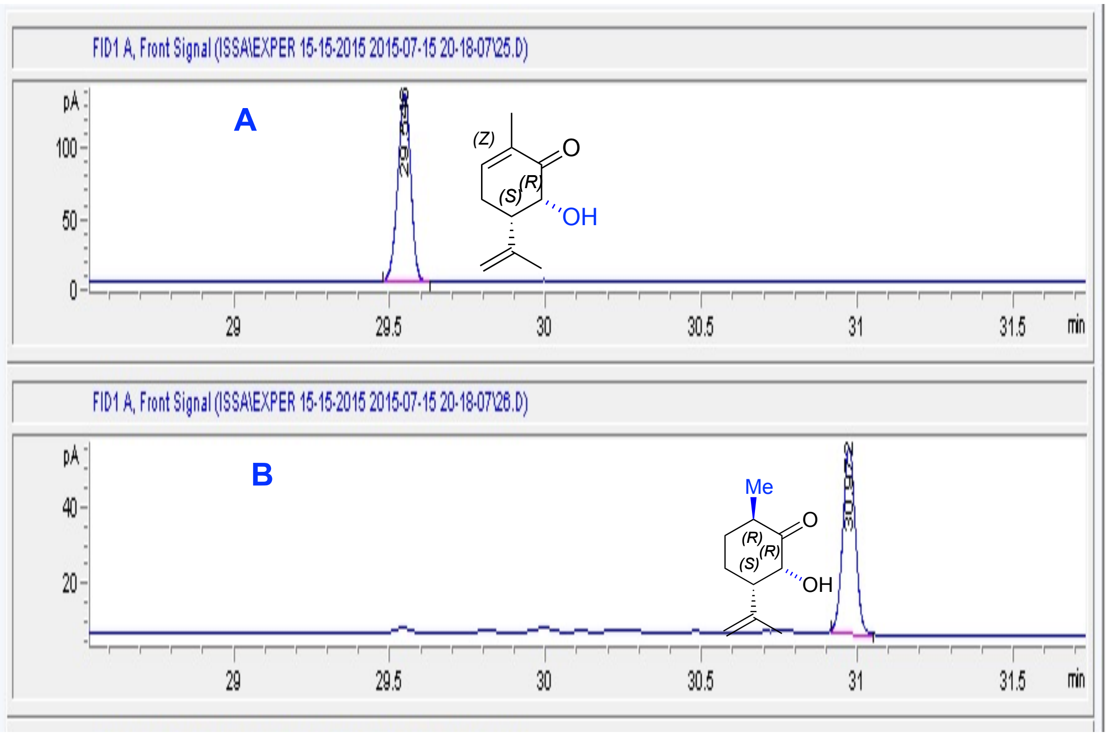

Figure S10. GC chromatographs of A) Control solution of 6-*R*-hydroxycarvone diastereomer (*syn*) **5** B) Bio-catalytic reaction mixture using PETNR Bio-reduction conditions: PETNR (10 μM), Substrate (5 mM) pH 7.0 0f 50 mM KP buffer solution, $NADP^+$ (15 μM), GDH (10 U), Glucose (15 mM), 30 °C at 130 rpm for 2 h.

3.3.4 Biocatalytic reduction of 6-*R*-hydroxycarvone diastereomer (*anti*) 8

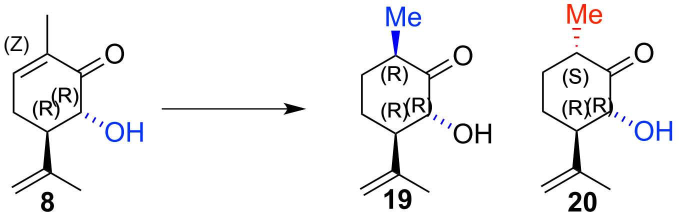

8 **19** **20**

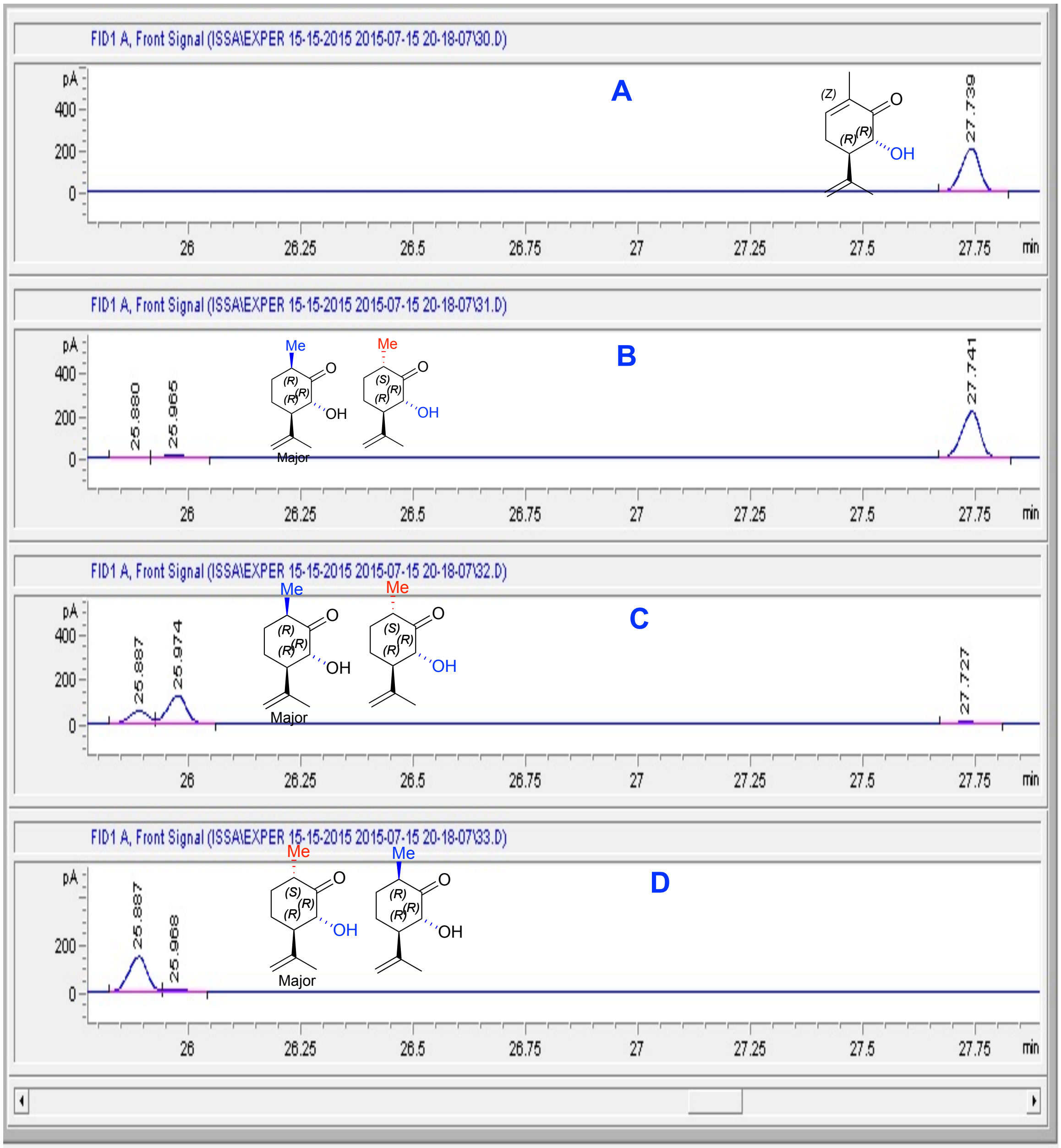

Figure S11. GC chromatographs of A) Control solution of 6-*R*-hydroxycarvone diastereomer (*anti*) **8** B) Bio-catalytic reaction mixture using OYE2 C) Bio-catalytic reaction mixture using PETNR D) Standard reference of diastereomeric mixture of the product (Chemically synthesized). Bio-reduction conditions: Enzyme (10 µM), Substrate (5 mM) pH 7.0 of 50 mM KP buffer solution, $NADP^+$ (15 µM), GDH (10 U), Glucose (15 mM), 30 °C at 130 rpm for 24 h.

3.4 Bio-oxidation of 6-methyldihydrocarvone using BVMOs

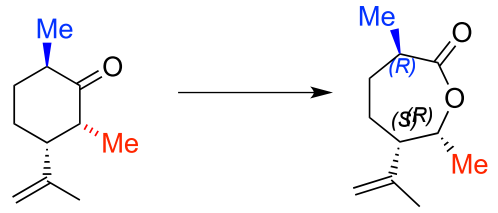

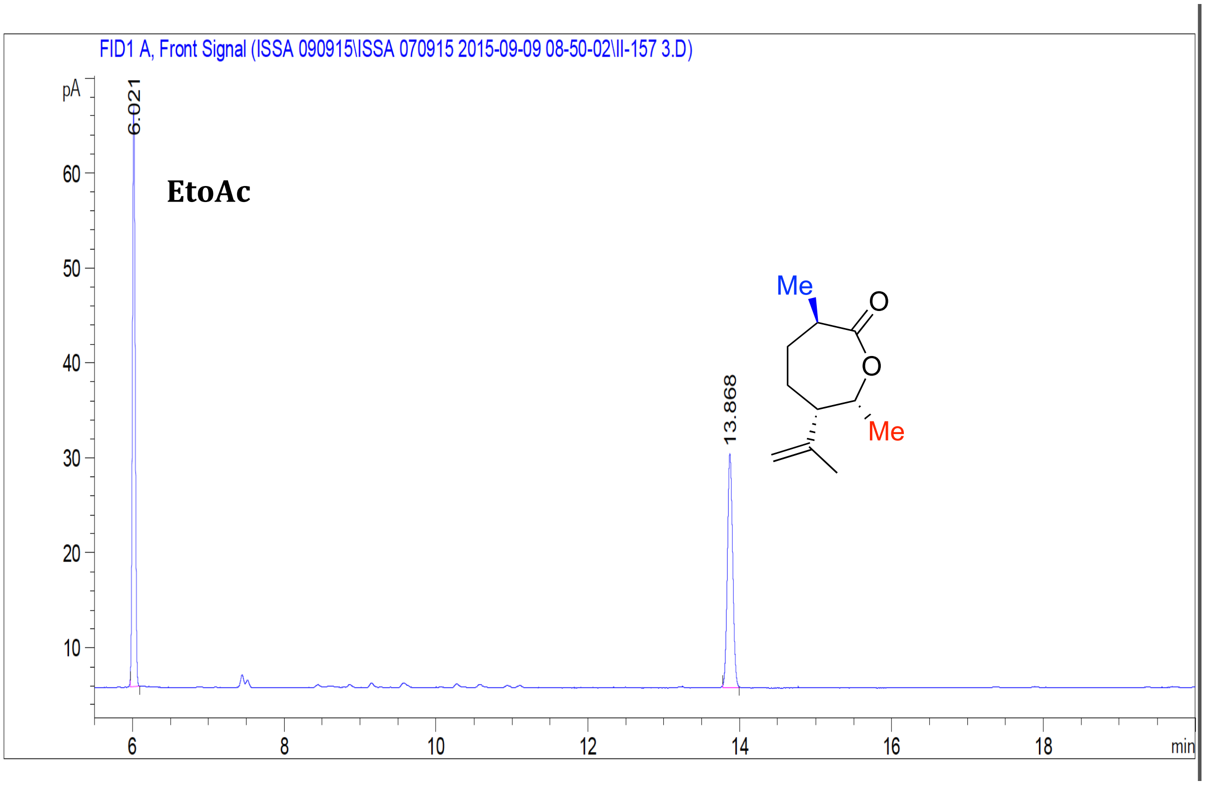

Figure S12. GC chromatograph of abnormal lactone **21** resulted from biooxidation of 6-Me-dihydrocarvone using CHMO_Phi1 from *Rhodococcus sp. Phi1*). Bio-oxidation conditions: Enzyme (2 µM), Substrate (5 mM) pH 7.0 0f 50 mM Tris.HCl buffer solution, $NADP^+$ (15 µM), GDH (10 U), Glucose (15 mM), 25 °C at 130 rpm for 24 h.

3.5 Structural analysis and Bio-oxidation of 3-methyldihydrocarvones using BVMOs

The structures of both isomers were incompletely confirmed through proton NMR but this was complicated due to overlapping of the peaks. Batey and co-workers separated two diastereomers (**23**:**22** = 7:5) of from radical opening ring of carvone-derived cyclopropyl ketone precursor, and proved the major one to be 23, and minor to be **22.**[10] The proton NMR of **23** assigned therein is consistent with assignment of **23** (minor isomer of that mixture in the current study). This supports that the mixture of two isomers, separated in this work, consists of **22** and **23**. Additionally, **22** and **23** have been reported (Siscovic and Roa[11]) in a 1:1 ratio through conjugate addition to carvone.

Our comparative NMR data of a mixture of these two isomers, versus a total mixtue of all 4 below allows a cross-analysis of the other minor components herein.

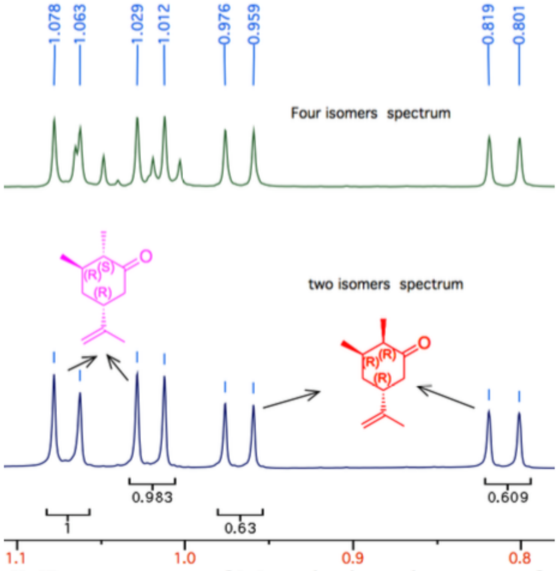

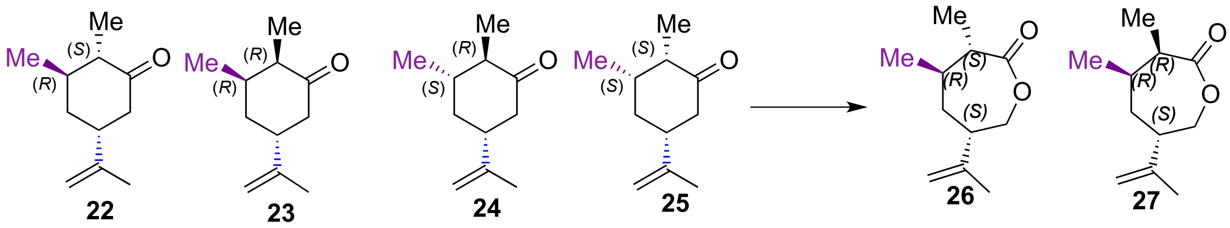

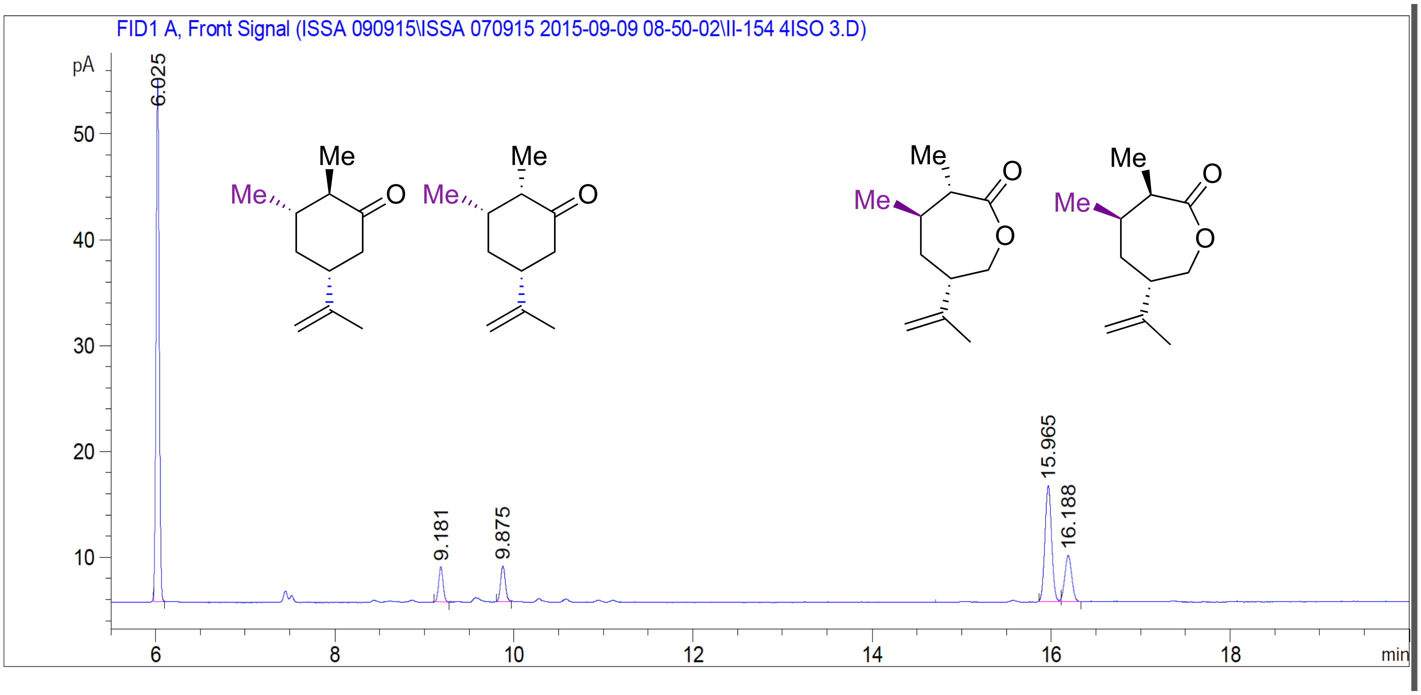

Figure S13. GC chromatographs of abnormal lactones **26 and 27** resulted from biooxidation of mixture of four isomers of 3-Me-dihydrocarvone using CHMO_Phi1 from *Rhodococcus sp. Phi1*). Bio-oxidation conditions: Enzyme (2 μM), Substrate (5 mM) pH 7.0 of 50 mM Tris.HCl buffer solution, $NADP^+$ (15 μM), GDH (10 U), Glucose (15 μM), 25 °C at 130 rpm for 24 h.

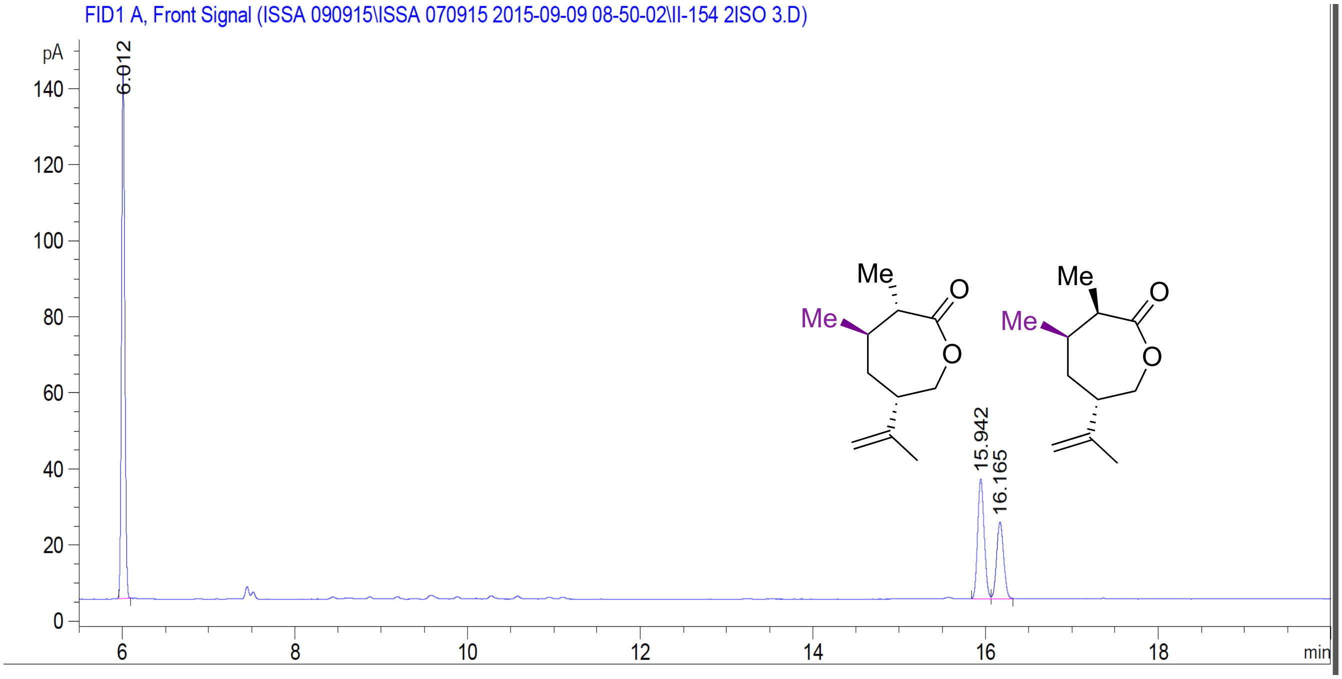

Figure S14. GC chromatographs of abnormal lactones **26 and 27** resulted from biooxidation of mixture of two isomers of 3-Me-dihydrocarvone using CHMO_Phi1 from *Rhodococcus sp. Phi1*). Bio-oxidation conditions: Enzyme (2 μM), Substrate (5 μM) pH 7.0 0f 50 mM Tris.HCl buffer solution, $NADP^+$ (15 μM), GDH (10 U), Glucose (15 μM), 25 °C at 130 rpm for 24 h.

4 Modelling

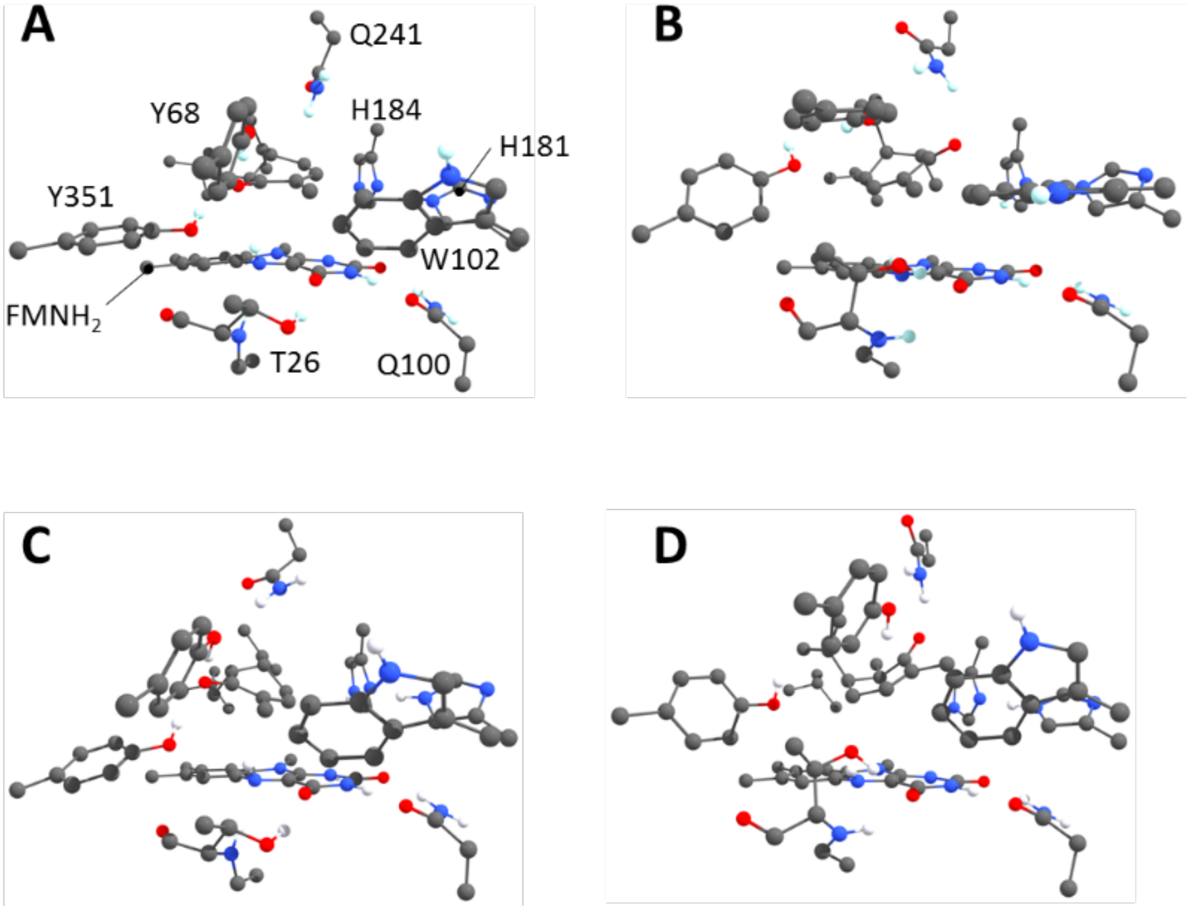

Figure S15. Optimised DFT models of PETNR active site with $FMNH_2$ and (A,B) 6*S*-Me carvone and (C,D) 6*R*-Me carvone bound in two conformations.

5 X-ray structure

Figure S16. X-ray crystal structure and stereochemistry of 6S-Methylcarvone **2**

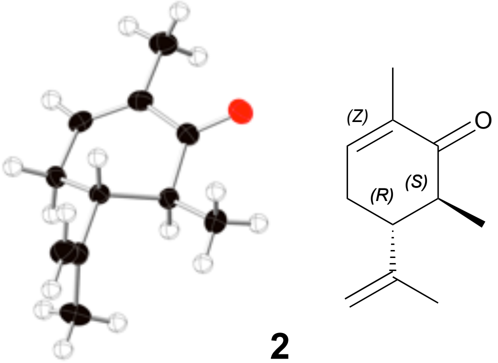

2

6. References

1. a) J. P. Gesson, J. C. Jacquesy, B. Renoux, *Tetrahedron* **1989**, *45*, 5853–5866; b) A. Srikrishna, D. Vijaykumar, *J. Chem. Soc. Perkin Trans.* **2000**, *1*, 2583–2589. C) Y. Chen, T. Ju, *Org. Lett.* **2011**, *13*, 86–89.
2. A. C. Lee, P. E. Floreancig, *Tet. Lett.* **2004**, *45*, 7193–7196.
3. R. Baudouy, C. Maliverney, *Tet.* **1988**, *44*, 471–480.
4. A. Srikrishna, G. Ragava, S. Danielooess, P. Hemamalini, *J. Chem. Soc., Perkin Trans. I*, **1996**, *48*, 1305–1311.
5. S. Gabriëls, D. V. Haver, M. Vandewalle, P. D. Clercq, A. Verstuyf, R. Bouillon, *Chem-Eur. J.* **2001**, *7*, 520–532.
6. a) J. Kabbara, S. Flemming, K. Nickisch, H. Neh, J. Westerman, *Chem. Ber*. **1994**, *127*, 1489–1493; b) S. Flemming, J. Kabbara, K. Nickisch, H. Neh, J. Westerman, *Synth. Commun*. **1994**, 317–320.
7. P. A. Karplus, K. M. Fox, V. Massey, *FASEB J.* **1995**, *9*, 1518–1526.
8. P. C. Brzostowicz, D. M. Walters, S. M. Thomas, V. Nagarajan, P. E. Rouviere, *Appl. Environ. Microbiol.* **2003**, *69*, 334–342.
9. T. Knaus, F. G. Mutti, L. Humphreys, N. Turner, N. S. Scrutton, *Org. Biomol.* Chem. 2015, 13, 223–233.
10. A. R. Batey, D. J. Harling, W. B. Motherwell, Tetrahedron **1996**, 52, 1421–1444.
11. E. Siscovic, A. S. Rao,Current Science **1968**, 37, 286.
